# Insect Resistance to *Bacillus thuringiensis* Toxin Cry2Ab Is Conferred by Mutations in an ABC Transporter Subfamily A Protein

**DOI:** 10.1371/journal.pgen.1005534

**Published:** 2015-11-19

**Authors:** Wee Tek Tay, Rod J. Mahon, David G. Heckel, Thomas K. Walsh, Sharon Downes, William J. James, Sui-Fai Lee, Annette Reineke, Adam K. Williams, Karl H. J. Gordon

**Affiliations:** 1 CSIRO, Black Mountain Laboratories, Canberra, Australian Capital Territory, Australia; 2 Department of Entomology, Max-Planck Institute for Chemical Ecology, Beutenberg Campus, Jena, Germany; 3 CSIRO, Australian Cotton Research Institute, Narrabri, New South Wales, Australia; 4 Department of Genetics, University of Melbourne, Parkville, Victoria, Australia; 5 Institute for Phytomedicine, Center of Applied Biology, Geisenheim University, Geiesenheim, Germany; Fred Hutchinson Cancer Research Center, UNITED STATES

## Abstract

The use of conventional chemical insecticides and bacterial toxins to control lepidopteran pests of global agriculture has imposed significant selection pressure leading to the rapid evolution of insecticide resistance. Transgenic crops (e.g., cotton) expressing the Bt Cry toxins are now used world wide to control these pests, including the highly polyphagous and invasive cotton bollworm *Helicoverpa armigera*. Since 2004, the Cry2Ab toxin has become widely used for controlling *H*. *armigera*, often used in combination with Cry1Ac to delay resistance evolution. Isolation of *H*. *armigera* and *H*. *punctigera* individuals heterozygous for Cry2Ab resistance in 2002 and 2004, respectively, allowed aspects of Cry2Ab resistance (level, fitness costs, genetic dominance, complementation tests) to be characterised in both species. However, the gene identity and genetic changes conferring this resistance were unknown, as was the detailed Cry2Ab mode of action. No cross-resistance to Cry1Ac was observed in mutant lines. Biphasic linkage analysis of a Cry2Ab-resistant *H*. *armigera* family followed by exon-primed intron-crossing (EPIC) marker mapping and candidate gene sequencing identified three independent resistance-associated INDEL mutations in an ATP-Binding Cassette (ABC) transporter gene we named HaABCA2. A deletion mutation was also identified in the *H*. *punctigera* homolog from the resistant line. All mutations truncate the ABCA2 protein. Isolation of further Cry2Ab resistance alleles in the same gene from field *H*. *armigera* populations indicates unequal resistance allele frequencies and the potential for Bt resistance evolution. Identification of the gene involved in resistance as an ABC transporter of the A subfamily adds to the body of evidence on the crucial role this gene family plays in the mode of action of the Bt Cry toxins. The structural differences between the ABCA2, and that of the C subfamily required for Cry1Ac toxicity, indicate differences in the detailed mode-of-action of the two Bt Cry toxins.

## Author Summary

Transgenic crops expressing the insecticidal protein Cry2Ab from *Bacillus thuringiensis* (Bt) are used worldwide to suppress damage by lepidopteran pests, often used in combination with Cry1Ac toxin to delay resistance evolution. Until now, the Cry2Ab mode of action and the mechanism of resistance were unknown, with field-isolated Cry2Ab resistant *Helicoverpa armigera* showing no cross-resistance to Cry1Ac. In this study, biphasic linkage analysis of a Cry2Ab-resistant *H*. *armigera* family followed by EPIC marker mapping and candidate gene sequencing identified three independent INDEL mutations in an ATP-Binding Cassette transporter subfamily A gene (ABCA2). A deletion mutation was identified in the same gene of resistant *H*. *punctigera*. All four mutations are predicted to truncate the ABCA2 protein. This is the first molecular genetic characterization of insect resistance to the Cry2Ab toxin, and detection of diverse Cry2Ab resistance alleles will contribute to understanding the micro-evolutionary processes that underpinned lepidopteran Bt-resistance.

## Introduction

In recent decades, agriculture has increasingly come to rely on toxins encoded by the Gram-positive bacterium *Bacillus thuringiensis* (Bt) for the production of insect–resistant transgenic crops. Narrow spectrum insecticides such as the protoxin crystals produced by Bt during sporulation are highly specific for certain insect groups including the Lepidoptera, Diptera and Coleoptera (e.g., [1]). Bt sprays have been used for many years, gaining widespread acceptance in pest management due to their relative target-specificity and their safety for humans, most other organisms, and the environment. However, the increasing cultivation of Bt transgenic crops poses a significant risk with various field populations of major lepidopteran pests reported to have developed resistance [2–4], threatening the sustainability of this strategy for crop protection. Indeed a major reason for the uptake of Bt was the evolution of resistance to chemical insecticides such as organochlorides, synthetic pyrethroids, and organophosphates in pests such as the cotton bollworm *Helicoverpa armigera*. This species is one of the most damaging and economically important lepidopteran pests known worldwide in a variety of crops, and one of four major pests in the genus *Helicoverpa*, the others being *H*. *punctigera*, *H*. *zea* and *H*. *assulta* [5–8].

For control of lepidopteran pests, genes encoding members of the Cry1A family were the first to be used in transgenic crops. However resistance alleles to the Cry1Ac toxin have been reported in field-collected *H*. *punctigera* from Australia [9,10], and *H*. *armigera* from China [11–13]. Resistance to Cry1Ac has also been reported in Indian populations of *Pectinophora gossypiella* [14] and *H*. *zea* from the New World [15–17]. Genetic studies on the field-derived strains have provided critical insight into the mode of action of this toxin, by identifying key receptors present on the surface of midgut epithelial cells (e.g., [18–23]; see also [24–26]). This binding of the activated toxins to specific receptors is crucial for formation of pores in the affected cells, leading eventually to the death of the larvae.

Resistance to the Cry1Ac toxin in the Lepidoptera was first shown to be associated with mutation of a gene encoding a 12-cadherin domain protein. Deletions of different lengths were observed in various regions of the gene in, e.g., *Heliothis virescens* [27], *P*. *gossypiella* [28] and *H*. *armigera* [11–13,24], as well as insertions by transposable elements [12,29,30]. Down regulation of *cis*-mediated transcription of the trypsin gene *HaTryR* allele due to mutations at the promoter region, mis-splicing of the ABCC2 gene, and a deletion mutation of the Aminopeptidase N (APN) gene have also been demonstrated to lead to resistance to Cry1Ac in *H*. *armigera* [31–33]. Recently, mutations in the ABCC2 gene belonging to Family C of the ATP-Binding Cassette (ABC) transporter family have also been shown to confer resistance to Cry1Ab and Cry1Ac in *H*. *virescens* [34], *Plutella xylostella* [35], *Bombyx mori* [36], *Spodoptera exigua* [37], and *H*. *armigera* [32]. Heterologous expression of ABCC2 from resistant and susceptible *B*. *mori* has shown that it aids in pore formation [38], and modification or deletion of ABCC2 is hypothesized to block the final step in the toxin's mode of action [39].

To prolong the efficacy of individual Bt toxins as transgenic control agents, multiple Bt genes, encoding different toxins with different modes of action, have been incorporated into plants. The second Bt gene adopted in many countries in transgenic plants has been a member of the Cry2A family, Cry2Ab. Cry1A resistance due to mutations in the cadherin or ABCC2 genes is not known to confer cross-resistance to Cry2Ab [22,34]. However, Cry2Ab resistance has now been reported in various lepidopteran pests (e.g., *P*. *gossypiella* [40]; *H*. *zea* [41]). In Australia, field-derived Cry2Ab resistance alleles in *H*. *armigera* and *H*. *punctigera* were first isolated in the summer of 2002/2003 and 2004/2005 respectively, and were used to establish the homozygous resistant lines SP15 [42] and Hp4-13 respectively [43]. All Cry2Ab resistant *H*. *armigera* and *H*. *punctigera* alleles isolated from Australia to-date have been shown to be recessive [9,21,42,43]. Isolates were captured using the "F_2_ screen" method [44] with a discriminating dose of Cry2Ab toxin, and confirmed as allelic by complementation tests [83]. These isolates are being used in F_1_ tests which involved crossing them to a field-collected insect (of unknown genotype), and screening the F_1_ offspring for resistance [45–47]. These F_1_ screens have estimated the frequency of Cry2Ab-resistance-conferring alleles in *H punctigera* and *H*. *armigera* field populations to range between 0.010 and 0.047, and 0.015 and 0.044, respectively, with no significant linear trend over time (from 2007 to 2014) [48–50].

The F_1_ and F_2_ screen techniques do not directly reveal the molecular identity of such resistance alleles, and the molecular basis of the Cry2Ab resistance is likely due to specific target-site alterations located within the midgut [21]. Whether the *H*. *armigera* and *H*. *punctigera* Cry2Ab resistance genes are homologous is not known, although parallel evolution in orthologous ABCC2 genes leading to Cry1Ac resistance in different species has been reported (e.g., [34,35]). A third generation of transgenic cotton (Bollgard III (BGIII)) expressing three Bt toxin genes (*Cry1Ac*, *Cry2Ab* and *Vip3A*) will soon become available in Australia. In light of these developments and whilst the Australian industry adopts a pre-emptive strategy to manage resistance to Bt, several key assumptions of this strategy are theoretically sound but empirically untested. Marker-assisted detection of the resistance alleles in insect populations will therefore not only enable a more efficient monitoring effort but will also enable assumptions about the ecology of resistance to be rigorously examined.

In this paper we report on the identification of the Cry2Ab resistance gene in *H*. *armigera* using linkage mapping and a chromosome walk with the assistance of exon-primed intron-crossing (EPIC)-PCR markers. This gene, which is expressed in the midgut, encodes an ABC transporter in the A subfamily—ABCA2—and is the likely site of mutations conferring resistance to Cry2Ab. By screening additional lab-isolated resistant lines derived from field-collected materials, we show that resistance to the Cry2Ab toxin in *H*. *armigera* occurred through independent evolutionary events involving different mutations, all of which were located in different exons of the same ABCA2 gene in both species. This work therefore provides the first insight into the detailed mode of action of a Cry2A toxin, which is conserved across different lepidopteran species, and is of considerable significance for the management of Bt resistance globally.

## Results

### Identification of a linkage group carrying the Cry2Ab resistance gene

**Fig 1:**
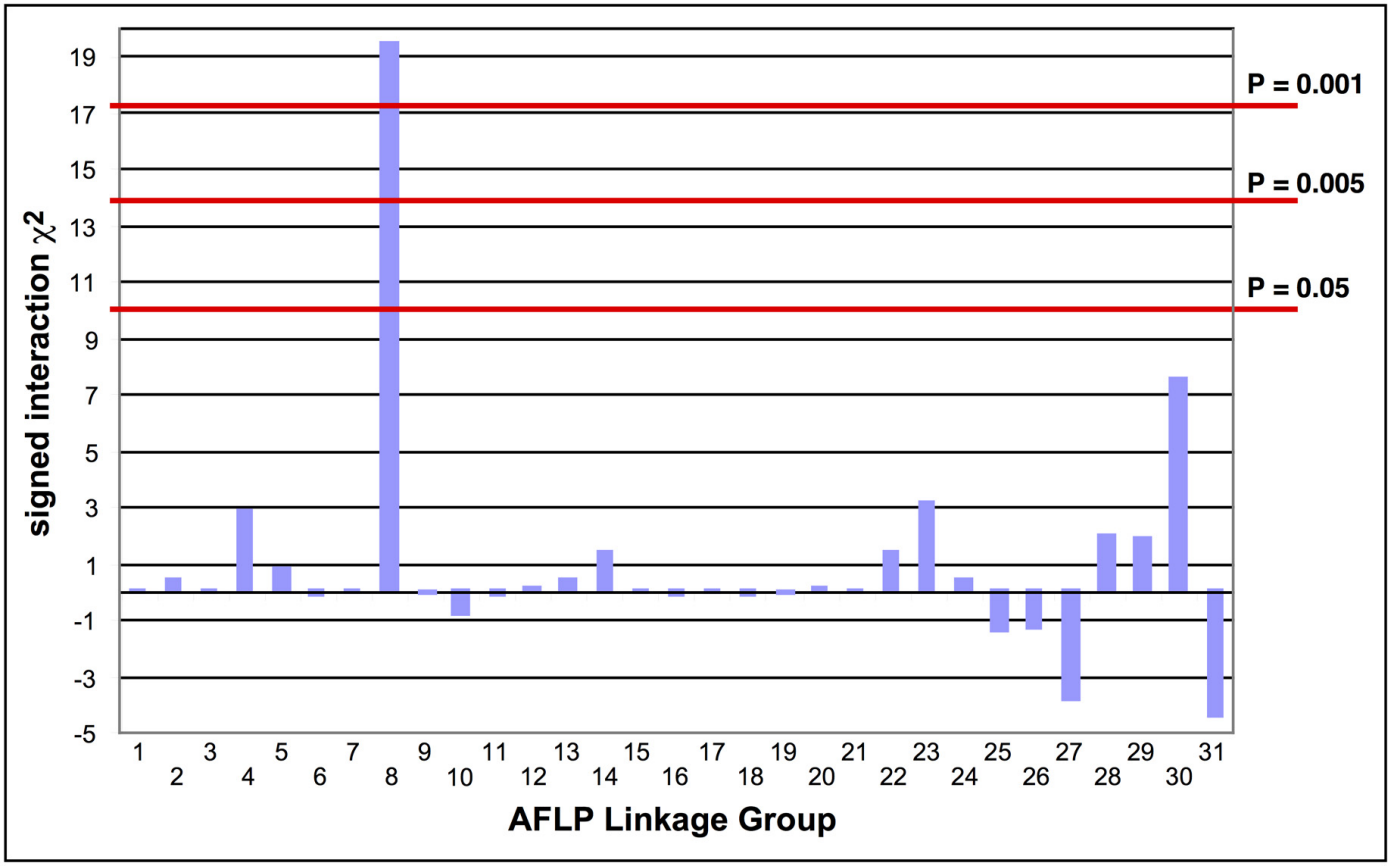
Tests of the association of AFLP linkage groups with Cry2Ab1 resistance in a bioassay of backcross progeny. The signed interaction χ^2^ value shows the positive or negative association of the AFLP linkage group from the resistant grandparent with resistance in the progeny. Horizontal lines depict the Bonferroni-corrected probability values of P = 0.05, P = 0.005, and P = 0.001. Only AFLP linkage group 8 shows a significant positive association with resistance.

The absence of crossing-over in female Lepidoptera makes it possible to map a recessive trait such as the Cry2Ab resistance in SP15 to a linkage group using biphasic linkage analysis with AFLPs as genetic markers [18]. Progeny from a female-informative backcross family were bioassayed with a discriminating dosage of Cry2Ab; 161 AFLPs segregating in this family were grouped into 31 independently assorting linkage groups. Linkage to Cry2Ab resistance was tested by comparing bioassayed survivors with untreated control progeny. Only AFLP linkage group (LG) 8 showed a significant association with resistance (χ^2^ = 19.44, P < 0.001; Fig 1); all 29 treated survivors were homozygous for the SP15 homolog of this linkage group.

**Fig 2:**
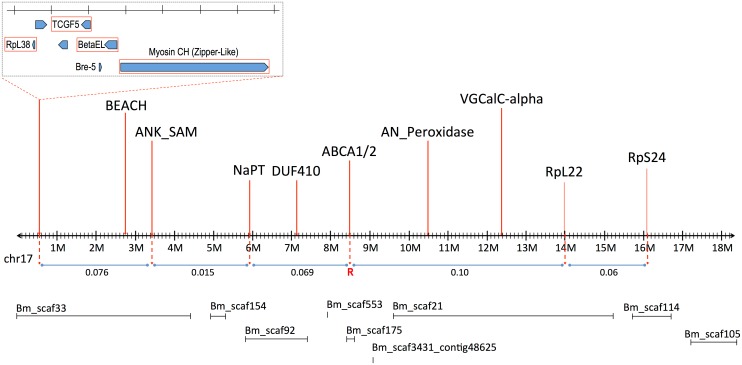
Linkage map of *B*. *mori* Chromosome 17 (BmChr17), showing syntenic genes used in mapping of *H*. *armigera*. Approximate recombination rates between genes are provided in relation to the putative Cry2Ab resistance gene, indicated by ‘R’. The *B*. *mori* scaffolds on BmChr17 are also provided as obtained from KAIKObase <http://sgp.dna.affrc.go.jp/KAIKObase/>.

Southern blot analysis of RFLPs in an unrelated Cry2Ab-susceptible *H*. *armigera* family showed that one AFLP from LG8 was linked to ribosomal protein gene *RpL22*. *RpL22* in *B*. *mori* is located on chromosome 17 (BmChr17, KAIKObase [51]). Using specific probes for additional ribosomal protein genes, the Cry2Ab-resistance-associated linkage group in *H*. *armigera* was also shown to carry genes for *RpL38* and *RpS24*, confirming homology with BmChr17 [52]. This assignment excludes a number of previously-identified genes as candidates for Cry2Ab resistance. Chromosomes harbouring homologs of previously-identified Cry1Ac resistance mutations in *H*. *virescens* include BmChr06 with the 12-cadherin-domain protein [27], BmChr15 with the ABCC2 protein [34], and BmChr21 with the *BtR-5* gene [53]. Moreover, genes for previously identified Cry1Ac binding proteins map to chromosomes other than BmChr17: several aminopeptidase genes are located on BmChr09 [54], a membrane-bound alkaline phosphatase gene maps to BmChr03 [23], and the P252 glycoprotein gene is on BmChr25 [55,56]. Although different levels of cross-resistance between Cry1A and Cry2A toxins have been reported in *H*. *armigera* from China [57,58], in *H*. *virescens* [59], *H*. *zea* and *P*. *gossypiella* ([40], see also [60]); independent segregation of BmChr17 relative to all of these other chromosomes is nevertheless consistent with the absence of cross-resistance between Cry1Ac and Cry2Ab in both the SP15 *H*. *armigera* [42] and Hp4-13 *H*. *punctigera* [9,10] lines, thereby supporting the notion that the two toxins have different modes of action. However, we found that the ortholog of the *bre-5* glycosyltransferase gene in a mutant of the nematode *C*. *elegans* resistant to the Cry4B toxin [19] is located on BmChr17 (Fig 2). This gene was therefore further investigated as a candidate gene for Cry2Ab resistance.

Additional linkage mapping in a male-informative backcross and two F_2_ families was performed to further localize the resistance locus. A preliminary map based on 72 progeny from these families gave the gene order and recombination values as follows:

Bre−5–(0.16)–Cry2Ab resistance locus–(0.10)–RpL22–(0.06)–RpS24

The order and spacing of the three marker loci was similar to that in *B*. *mori* on BmChr17. However, the large fraction of recombinants between the resistance locus and *bre-5* ruled out the latter as a candidate (Fig 2).

### Linkage mapping

The Cry2Ab resistance gene was further localised within BmChr17 using recombinational mapping in backcrosses with F_1_ males. For this work, markers were developed from *H*. *armigera* orthologs for genes mapped along BmChr17. Sequences allowing design of EPIC-PCR primers for the *H*. *armigera* orthologs for these genes were obtained from transcriptome sequencing of midgut RNA extracted from larvae of the GR susceptible colony.

Recombinational analysis of selected markers in *H*. *armigera* showed the linkage order of these markers to be the same as in *B*. *mori* (Fig 2), greatly assisting the subsequent analysis which employed the *B*. *mori* genome as a reference framework. Analysis of recombination rates between 3 of these markers (*RpL38*, *Zip2*, *VGCal-A*) and the Cry2Ab resistance allele placed it between *Bre-5* (BGIBMGA005534) and *RpL22* (BGIBMGA006986, at nt 14152986). The markers for the voltage-gated channel protein gene (orthologous to BGIBMGA007009, starting at 12341859 on BmChr17 –see S1 Table) further restricted the area containing the resistance gene. The target area could however be more narrowly defined, since the gene is ~10cM from BGIBMGA005534 and ~16cM from BGIBMGA006986; these genes are located at ~3Mbp and ~10Mbp respectively on the BmChr17 sequence (see S1 Table). In fine scale analysis of this region, markers for the genes BEACH, ANK_SAM, NaPT, DUF410, and AN_Peroxidase all showed recombination with the resistance trait. Of these, the marker for DUF410 (BGIBMGA007299, located at 7124451 on BmChr17) most closely restricted the target region on the proximal side, corresponding to less than 3Mbp of BmChr17, and containing fewer than 30 genes (S1 Table).

Two ABC transporter A subfamily genes are located adjacently between nts 8466000–8564000 on BmChr17. The first of these, termed BmABCA1, is well-predicted as BGIBMGA007221, while the other, BmABCA2, includes the partial predictions BGIBMGA007218 and BGIBMGA007217 (see [61,62] for analysis of the original uncorrected gene models). The sequence of HaABCA1, the *H*. *armigera* ortholog to the BGIBMGA007221 BmABCA1 gene, was obtained from RNAseq libraries, from total larvae of the susceptible GR colony. The EPIC-PCR marker for HaABCA1 (ABCA1; S2 Table) gave a genotype profile consistent with tight linkage to the Bt Cry2Ab resistance allele; the F_2_ bioassayed offspring (homozygous allele size of 272bp / 272bp) was identical to the SP15 grandmother (272bp / 272bp) in 100% of all samples tested (final n = 72). The GR grandfather was heterozygous with alleles 264bp / 282bp, leading to the F_1_ male being heterozygous with allele sizes 272bp / 282bp. The F_2_ control (n = 20) gave the expected 50:50 ratio with n = 11 being 272bp / 272bp homozygous and n = 9 heterozygous (272bp / 282bp). This tight linkage between the HaABCA1 gene and resistance made it a candidate for being the target of the resistance mutation.

### Identification of resistant alleles

**Fig 3:**
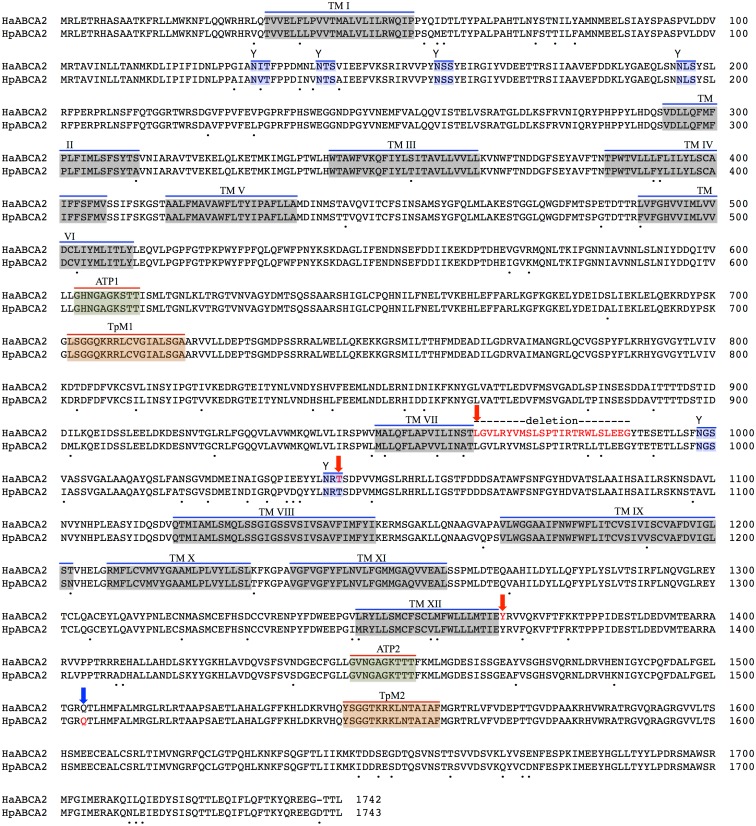
Predicted protein sequence of HaABC2. Feature predictions include 12 transmembrane helices (TM I to XII), two ATP-binding domains ATP1 and ATP2, two transporter motifs TpM1 and TpM2, and predicted glycosylation sites (highlighted in blue, and indicated by ‘Y’). Amino acid differences between *H*. *armigera* and *H*. *punctigera* are indicated by ‘.’ mutation sites are indicated by red and blue arrows for *H*. *armigera* and *H*. *punctigera* respectively—Ha2Ab-R01 (amino acid position 964); Ha2Ab-R02 (amino acid position 1,043); Ha2Ab-R03 (amino acid position 1,368); and Hp2Ab-R04 (amino acid position 1,504).

To assess whether HaABCA1 was the real location of resistance mutations, we checked whether this gene is expressed in the midgut. No evidence for significant midgut expression of ABCA1 was found in either *B*. *mori* (Bm-MDB, *B*. *mori* Microarray Database [63]; see S1 Table) or *H*. *armigera*, making it unlikely that this gene is actually involved in resistance. Initially detected in total larval and pupal transcripts, its expression was more specifically evident in larval foregut, hindgut, trachea and haemocytes. However the adjacent ABCA2 gene was significantly expressed in the midgut of both *Bombyx* and *H*. *armigera*; of the genes in this region of BmChr17, it is among the most highly expressed in the midgut [63] (see S1 Table). The full-length transcript of the *H*. *armigera* ortholog HaABCA2 (Fig 3) encodes a protein of 1,742 amino acids with 67.28% identity to the BmABCA2 gene in *B*. *mori* (S1 Fig).

**Fig 4:**
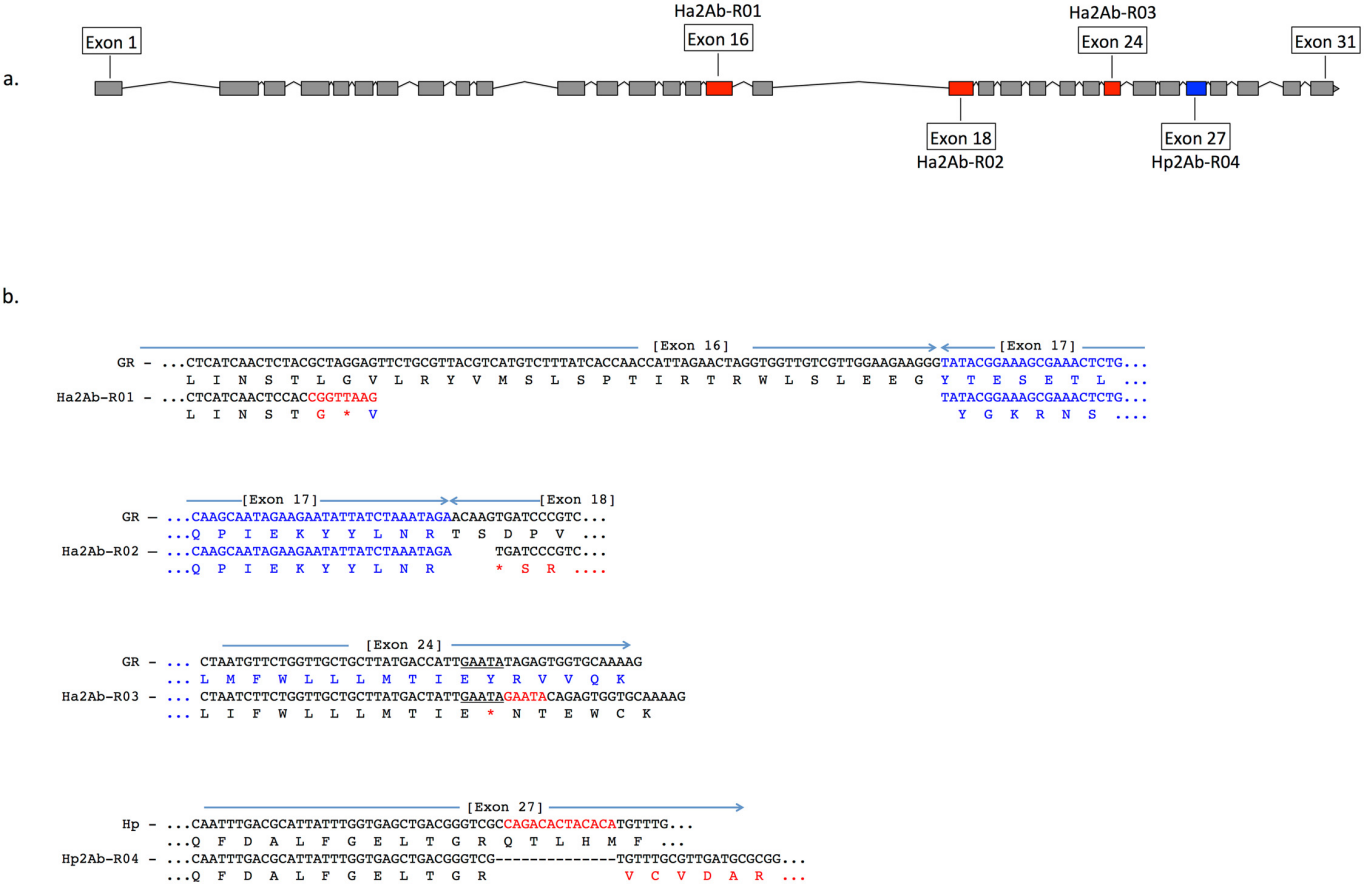
A summary of the three Cry2Ab resistance alleles (Ha2Ab-R01, R02, R03) in the ABCA2 gene in *Helicoverpa armigera*, and one *H*. *punctigera* Cry2Ab resistance allele (Hp2Ab-R04). The ABCA2 gene consists of 31 exons (Fig 4a), with mutations at exons 16, 18, 24 and 27 indicated by red (for *H*. *armigera*) and blue (for *H*. *punctigera*) boxes. Fig 4b: The Ha2Ab-R01 allele was the result of a 73 base pair (bp) deletion at the c-terminus of exon 16, and the insertion of a 8bp ‘CGGTTAAG’ sequence, and resulted in a glycine (G) replacing the leucine (L) amino acid followed by a premature stop codon (*) (in red). The Ha2Ab-R02 allele was the result of a 5bp ‘ACAAG’ deletion at the start of exon 18 that resulted in a premature stop codon. The Ha2Ab-R03 allele was the result of a 5bp ‘GAATA’ target site duplication signature reminiscent of past insertions by transposable elements. This duplication mutation also resulted in a premature stop codon in exon 24. The *H*. *punctigera* Hp2Ab-R04 allele was the result of a 14bp deletion, and resulted in missense mutations.

We therefore further explored whether any changes were evident in the transcripts of the ABCA2 gene in midgut RNA from larvae of the resistant line. The ABCA2 candidate gene cDNA was fully sequenced from a SP15 resistant individual that identified a 73bp deletion at exon 16 (‘ G CTA GGA GTT CTG CGT TAC GTC ATG TCT TTA TCA CCA ACC ATT AGA ACT AGG TGG TTG TCG TTG GAA GAA GGG’ from nucleotides 2,889–2,961) / 8bp (‘C GGT TAA G’) insertion mutation (= allele 1, Ha2Ab-R01) (Fig 4a and 4b) of the coding sequence which resulted in the replacement of leucine (L) by glycine (G) at position 964, followed by a stop codon downstream from the mutation site at position 965. The 8bp ‘C GGT TAA G’ nucleotides matched completely to part of intron 16 (intron 16 nucleotide positions 56 to 62) of the SP15 *H*. *armigera* line. From the sequence of this mutated cDNA, we designed primers to screen additional independently isolated field resistant lines from 2005 (line 5–405); 2006 (lines 6–364 and 6–798), 2009 (line 9–4802), 2010 (line 10–485), and 2012 (line 12–2169), four of which (i.e., 5–405, 9–4802, 10–485, 12–2169) showed the same 73bp deletion/8bp insertion mutation as identified in the SP15 individual. A second resistance allele (= Ha2Ab-R02) with a 5bp (‘ACA AG’) deletion mutation at nucleotides 3,127–3,131 of the coding sequence was identified in a homozygous individual from the Cry2Ab resistant lines 6–364. A heterozygous resistant individual from line 6–798 was identified to possess one Ha2Ab-R02 allele, and a third resistance allele (= Ha2Ab-R03) at nucleotides 4,104–4,108 that represented a 5bp (GAATA) nucleotide duplication (Fig 4b), similar to the target site duplication (TSD) signature that is widespread in the *H*. *armigera* genome due to transposable element transposition activities (e.g., see [64]). cDNA sequencing of both the 6–364 and 6–798 lines identified the presence of the Ha2Ab-R02 allele as homozygous in the 6–364 line, and also both Ha2Ab-R02 and Ha2AB-R03 alleles at exons 18 and 24 respectively in line 6–798, thereby confirming that this resistant line was heterozygous for the ABCA2 gene (see S2 Fig). All three mutations identified to date in the Cry2Ab resistant lines are located at the 3’ region of the 5.1Kb coding sequence, and result in truncation of the protein. A summary of the three Cry2Ab resistance alleles identified in *H*. *armigera* is presented in Fig 4a and 4b. Sequence analyses of the SP15, 6–364 and 6–798 resistant individuals confirmed that no other INDELs or nonsense mutations were present in coding regions of the ABCA2 gene. Similarly, additional sequence analyses from multiple susceptible *H*. *armigera* individuals showed the predicted fully functional (non-truncated) ABCA2 gene, while nucleotide variation (<1%) between Cry2Ab susceptible *H*. *armigera* ‘GR’ lines resulted in nine amino acid changes, six of which involved nonsynonymous substitutions between amino acids with hydrophobic side-chains (e.g., valine (V), isoleucine (I), tyrosine (Y), phenylalanine (F), methionine (M), and leucine (L)), and one amino acid substitutions of each between lysine (K) and threonine (T), alanine (A) and proline (P), and glycine (G) and glutamic acid (E) (S2 Fig).

### Absence of HaABCA2 mutations in susceptible field-collected insects

To confirm the significance of the HaABCA2 mutations in resistant lines, we asked whether susceptible individuals collected from the field carried the same INDEL mutations or other inactivating mutations in the *H*. *armigera* ABCA2 gene. Starting with single pairs of field-collected insects, F_1_ pools from each mating pair generated F_2_ progenies (n = 90) that were screened against a discriminating dose of Cry2Ab and identified those pairs whose F_2_ progeny all died as carrying only susceptible alleles. This test is enough to exclude any resistance-conferring alleles (occurring as heterozygotes) amongst the grandparents. Ten individual 3^rd^ instar larvae representing 10 different field-collected susceptible populations were RNA extracted and RT-PCR used to generate cDNA. To screen for any evidence of mutations in the ABCA2 gene, PCR of the cDNA and sequencing using appropriate primer pairs (see S1 Table) were performed. No evidence for any inactivating mutations was found in any of these 10 individuals, i.e. all contained untruncated transcripts at exons 16, 18 and 24 where Ha2Ab-R01, R02 and R03 alleles were detected, respectively.

### Protein domain prediction

**Fig 5:**
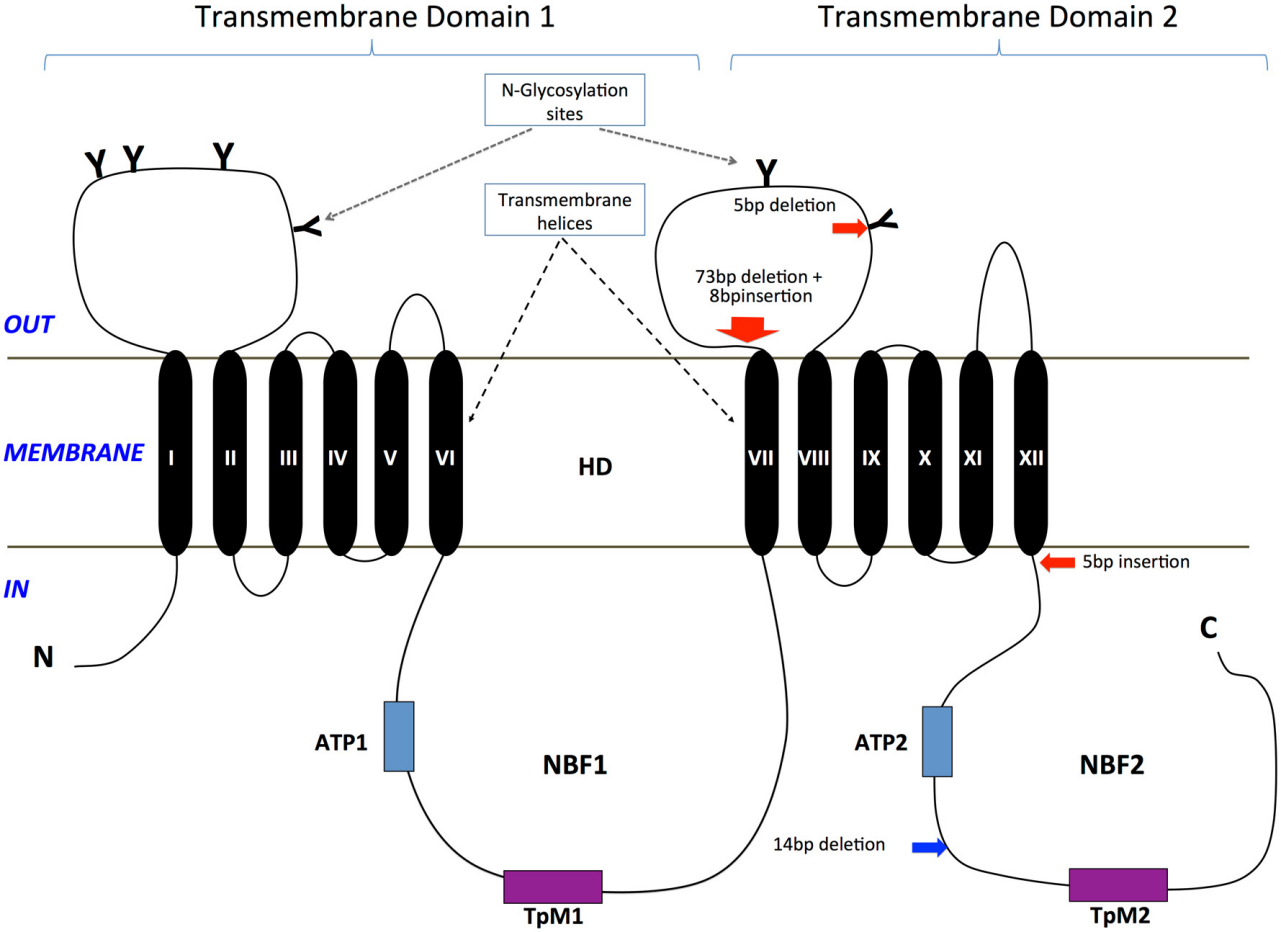
Diagram of the ABCA2 protein structure and location of mutations in *H*. *armigera* and *H*. *punctigera*. Glycosylation sites on the two large extracellular loops are represented by ‘Y’. Two highly conserved ATP Nucleotide Binding Folds (NBF1, NBF2) that included the Transporter signature motifs 1 and 2 (TpM1, TpM2) are present in the intracellular environment. The *Helicoverpa* ABCA2 protein structure consists of two transmembrane domains (TMD 1, TMD 2), each with six transmembrane helices (TM I-VI in TMD 1; TM VII-XII in TMD 2). The approximate positions of the mutations in *H*. *armigera* and *H*. *punctigera* are indicated by red and blue arrows, respectively.

A total of two transmembrane domains (TMDs; i.e., TMD 1, TMD 2) with each consisting of six transmembrane helices (TM I-VI in TMD 1; TM VII-XII in TMD 2, Fig 5) were predicted from the HaABC2 sequence that corresponded to those characteristic of the ABC transporter subfamily A. As for other members of this subfamily, N-glycosylation sites were also predicted for both of the extracellular domain (ECD) loops between TM I and TM II, and between TM VII and TM VIII (Figs 3 and 5). The intracellular loop between TMD 1 and TMD 2 (i.e., between TM VI and TM VII) and after TM XII contained the highly conserved regions for ATP Nucleotide Binding Fold 1 (NBF1) (including the Transporter signature Motif 1; TpM1), and NBF2 (and TpM2), respectively.

Each of the mutant alleles Ha2Ab-R01, Ha2Ab-R02 and Ha2Ab-R03 introduced stop codons into the reading frame, causing significant truncations of the HaABCA2 protein (Fig 4). The first two mutations introduced stop codons in the extracellular loop between TM VII and TM VIII of TMD 2, whereas the 5bp insertion that resulted in the Ha2Ab-R03 allele occurred just one amino acid after TM XII of TMD 2. All three mutations therefore truncated the protein before the second nucleotide-binding domain NBF2 (Fig 5), which would render the ABC transporter completely inactive, even if the protein were expressed and integrated into the cell membrane.

### Analysis of a mutation in *H*. *punctigera*

In the Cry2Ab-resistant *H*. *punctigera* Hp4-13 strain, the allele (Hp2Ab-R04) encoding the homolog of HaABCA2 was found to contain a 14bp deletion (Fig 4). This deletion disrupts the coding region of the transcript by introducing frame shifts that lead to a missense mutation and to the loss of the TpM2 transporter motif at the NBF2 (Figs 4 and 5). Although linkage mapping was not performed to conclusively associate this mutation with the resistant phenotype in *H*. *punctigera*, the fact that the same gene is mutated in a resistant strain in this species strongly supports the role of this gene as the target of mutations conferring resistance to Cry2Ab.

### Identification of ABCA1 and ABCA2 orthologs in other lepidopteran genomes

**Fig 6:**
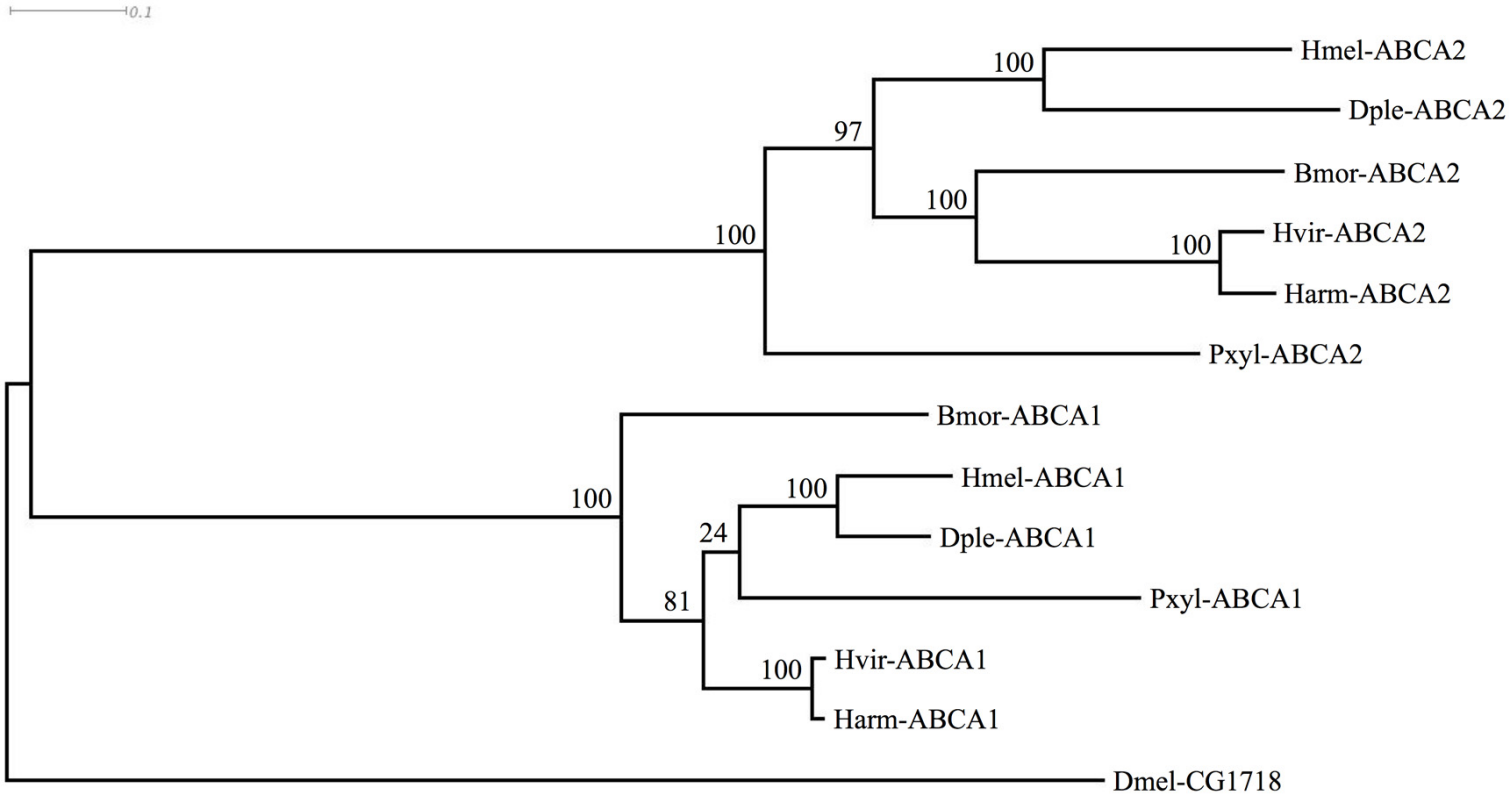
Phylogenetic tree showing clustering of ABCA1 and ABCA2 proteins of various Lepidoptera, with *Drosophila* homolog. Species abbreviations are: Bmor, *Bombyx mori*; Harm, *Helicoverpa armigera*; Hvir, *Heliothis virescens*; Pxyl, *Plutella xylostella*; Dple, *Danaus plexippus*; Hmel, *Heliconius melpomene*; Dmel, *Drosophila melanogaster*. The tree is based on the alignment shown in S1 Fig

We next asked whether sequences for these ABCA proteins existed in genomes of other Lepidoptera, including some (*H*. *virescens* [65], *Plutella*. *xylostella* [66]; *B*. *mori* [67]) known to be susceptible to Cry2Ab. We examined the published genome sequences of *Danaus plexippus* [68], *Heliconius melpomene* [69], and *P*. *xylostella* [70,71]. Existing predictions of the genes were often inaccurate, e.g., as with the two partial predictions for BmABCA2, and ABCA1 was predicted as two separate partial proteins for the *D*. *plexippus* genome [68]. We used Scipio [72] and FGENESH [73] as well as additional transcriptomic data to generate complete predictions (GenBank Accession numbers KP219762-KP219770). In each species, the ABCA1 and ABCA2 genes were present, and situated adjacently in a tail-to-tail orientation just as in *B*. *mori*. Orthology was further confirmed by conserved flanking genes; in each species the ortholog of *B*. *mori* mitochondrial ribosomal protein S7 (GenBank Accession XP_004927481) occurred upstream of ABCA2, and the ortholog of *B*. *mori* ubiquitin carboxyl-terminal hydrolase (GenBank Accession XP_004927480) occurred upstream of ABCA1. An alignment of the predicted ABCA1 and ABCA2 proteins along with the respective *Drosophila* protein showing greatest similarity (S1 Fig) was used to construct a phylogenetic (ML) tree. This tree indicated that the gene duplication creating ABCA1 and ABCA2 likely occurred in the common ancestor of the Lepidoptera shown (Fig 6).

## Discussion

The importance of Bt toxins for insect pest and disease control has stimulated enormous interest in the study of their mode of action. For Cry1A toxins, there is specific and saturable binding to membrane targets, and a sequential mode of action has been proposed [25,39]. The toxin first binds to the 12-cadherin domain protein, resulting in processing and accelerated oligomerization before binding to membrane-bound glycosylated proteins such as aminopeptidases, alkaline phosphatase and other glycoproteins [25,26]; the integral membrane ABCC2 protein then facilitates pore insertion. Cry1A and Cry2A proteins have comparable three-domain structures [74,75], making them likely to act in similar ways as pore-forming toxins. Specific and saturable binding to membranes was also recently shown for Cry2Ab [22,75], and resistance is associated with a loss of binding [21]. Despite these similarities, toxicity of Cry2Ab in general is unaffected by mutations conferring Cry1Ac resistance. Specifically, mutations in APN or cadherin or ABCC2 do not render insects resistant to Cry2Ab, so that this toxin must be binding to one or more different targets.

The identification of ABCA2 suggests a mode of action of Cry2Ab differing slightly from that proposed for Cry1A toxins. ABCA2 carries two extracellular domains that are present as long loops between helices TM I and TM II, and between helices TM VII and TM VIII (Figs 3 and 5). Both of these loops are glycosylated in mammals [76], and six glycosylation sites are predicted for HaABCA2 (Fig 3). In contrast, for the lepidopteran ABCC2, the corresponding loops are very short and contain no glycosylation signals [34]. We hypothesize that Cry2Ab also has a sequential mode of action in which the ABCA2 protein itself is able to provide both binding and pore insertion functions. Specifically, Cry2A toxins would, upon activation [75] bind to the glycosylated ECD loops in TMD 1 and/or TMD 2. This binding could form the basis of oligomerization and bring the pre-pore structure close to the TMDs for pore insertion, as proposed for ABCC2 [34]. It is possible that other proteins may also be involved in Cry2Ab binding and pore formation, particularly since mammalian ABCAs have been suggested to occur in multi-protein complexes in the membrane [77]. Interestingly, the ABCA2 mutations confer resistance to very high concentrations of Cry2A [42], as would be expected if both receptor and pore insertion functions are simultaneously blocked. Similarly, in *H*. *virescens* the ABCC2 mutation results in higher levels of resistance to Cry1Ac than does the mutation in cadherin, but when both are homozygous in the same strain and both receptor and pore insertion functions are blocked, extremely high resistance levels result [34].

To what extent are these findings likely to apply to other Bt toxins of the Cry2A family? Phylogenetic analyses based on the shared common three-domain structure [78] showed that Cry2Aa and Cry2Ai are sister toxin groups and occupy a basal position to both Cry2Ab/Cry2Ag and Cry2Ae/Cry2Ah clades. Cross-resistance between Cry2Ab and Cry2Aa has been demonstrated in the SP15 strain of *H*. *armigera* [42], and between Cry2Ab and Cry2Ae in both *H*. *armigera* and *H*. *punctigera* [21]. Resistance to Cry2Aa has also been identified in *H*. *virescens* [53,79], in *H*. *zea* [15], in *P*. *gossypiella* [40], and in *Ostrinia nubilalis* [80]; ABCA2 remains to be investigated in these species. Cry2A toxins are also toxic to some Diptera [81,82] (but see [83]), with Cry2Ab recently shown to be effective against the malaria mosquito vector *Anopheles gambiae* [82]. Cry2Ab was ineffective against *Aedes aegypti* [82] but the similar toxin Cry2Ag was highly effective [84].

Seven ABC transporters of the A subfamily are present in the *B*. *mori* genome (three on BmChr17, and one each on chromosomes 5, 14, 16, and 19; see [61,62] for analysis of the initial gene models), and similar numbers have been found in other Lepidoptera with sequenced genomes. This subfamily has been well characterised in vertebrates; there are 12 members known in humans [85] and a similar number in the mouse; the nomenclature for these differs from that of insects. The HaABCA2 gene and the other insect genes shown in Fig 6 belong to an insect-specific clade within this subfamily, with no direct orthologs among the vertebrate genes [86]. The human ABCA genes have been extensively analysed and are expressed in a variety of tissues, with most being involved in lipid transport and trafficking. Mutations in human ABCA2 (not orthologous to lepidopteran ABCA2) are associated with early-onset of Alzheimer’s disease [87,88]. The mouse ABCA2 (an ortholog of the human ABCA2) has a possible role in regulating cholesterol homeostasis and low-density lipoprotein receptor metabolism in N2 neuroblastoma cells [89], with knock-out causing a ‘shaky’ (tremor) phenotype [90]. Mutations in the mouse ABCA3 gene, which is expressed in lung tissues, are associated with a foetal surfactant deficiency that is fatal. However, in *H*. *armigera* (but not *H*. *punctigera*), the Cry2Ab resistant line homozygous for ABCA2-inactivating mutation has no demonstrated substantial fitness costs compared to Cry2Ab susceptible insects [91]. Whether this is due to functional redundancy with another of the midgut-expressed ABCAs remains to be determined.

The frequency distribution of resistant ABCA2 alleles identified to-date is non-uniform. Seven resistant *H*. *armigera* lines, isolated independently from the field between 2002 and 2012, produced three resistant alleles. The Ha2Ab-R01 allele was present in five lines: SP15, 5–405, 9–4802, 10–485 and 12–2169; the Ha2Ab-R02 allele was homozygous in 6–364 and present in heterozygotes in 6–798; the Ha2Ab-R03 allele was the alternative allele in the heterozygous 6–798 line. A single mutant allele, Hp2Ab-R04, was found in the one *H*. *punctigera* resistant line HP4-13. Thus some alleles in *H*. *armigera* were common enough to be recovered several times from the field. Whether this is due to some selection by an unknown agent in the Australian environment as proposed [92] remains to be tested.

The still-rare resistance-conferring alleles identified in field populations occur at a limited number of locations in the gene. If confirmed by studies of further alleles, this raises the possibility that DNA-based screens will allow monitoring of the spread of Bt resistance in *H*. *armigera* and *H*. *punctigera*. Although PCR-based screens [29,93] for mutations in the 12-cadherin domain protein of *H*. *armigera* that confers resistance to Cry1Ab and Cry1Ac identified the same allele (r1) in field material from northern China as that originally identified as conferring resistance [12], this has not always been the case. For *H*. *virescens* [94], for example, different mutations in the same gene were identified by the F_1_ screen (i.e., mating field-caught individuals with an existing homozygous resistant strain and testing the F_1_ offspring). For *P*. *gossypiella*, screening in India [95] found different cadherin gene mutations to those originally identified in Arizona [96].

Every Cry2Ab-resistant line from an F_2_ screen that has been molecularly characterized has shown mutations in the ABCA2 gene. This confirms the value of using the less expensive F_1_ screen with ABCA2-mutant lines to extend the estimation of Cry2Ab resistance allele frequencies in Australia. Incorporating PCR-based screening will further improve detection efficacies of ABCA2-based resistance in the field, enabling more accurate and faster estimates of resistance allele frequencies, and is especially relevant for the analysis of historical field material generated through F_1_/F_2_ screening methods [43,92], and for tracking spatial and temporal movement patterns of resistance alleles across the landscape. Further characterisation of resistance-conferring ABCA2 alleles will also help to resolve the current discrepancy between the F_2_ screen and the F_1_ screen in estimating allele frequencies [91]. It will be important to determine whether Cry2Ab-resistance-conferring ABCA2 mutations occur in *H*. *armigera* elsewhere in its geographic range, including its recent incursions into the Americas [97,98]. Finally, examination of ABCA2 may provide insight in several species where the Cry2A resistance mechanism is still unknown, including *H*. *virescens* [53], *P*. *gossypiella* [40], *H*. *zea* [41], and *Trichoplusia ni* [99].

## Materials and Methods

### Linkage mapping—Strains and families

As a result of an F_2_ screen in 2002, the first *H*. *armigera* Cry2Ab resistant strain (Sp15) was established from a single pair of moths collected as eggs on corn near Griffith, New South Wales (NSW), Australia [42]. Detailed descriptions of the techniques employed have been provided [42,92]. F_1_ progeny from that pair were intercrossed and the resultant F_2_ larvae exposed to a screening concentration of Cry2Ab in ground leaf material of the cotton variety Sicala V-2 transformed with the *B*. *thuringiensis* variety *kurstaki cry2Ab* gene construct. Survivors among the F_2_ formed the basis of the resistant colony Sp15. Since 2003, F_2_ screens with *H*. *armigera* and *H*. *punctigera* performed as part of a resistance monitoring program have isolated additional lines [10]. The isolated *H*. *punctigera* Cry2Ab resistant line (Hp4-13) was established from eggs collected at St George, Queensland, Australia in 2004 [43]. Complementation tests for allelism established that one or more alleles at the same locus conferred resistance to Cry2Ab in five lines of *H*. *armigera* derived from the field in Australia from 2002 to 2006, including SP15 and 5–405 (previously named NA405) [100].

### Identification of candidate Cry2Ab resistance AFLP linkage group

Assignment of the Cry2Ab resistance locus to an AFLP linkage group was carried out using the resistant line SP15 and the susceptible GR line. The initial cross was a SP15 Cry2Ab r/r ♂ x GR Cry2Ab s/s^-^♀, yielding family G. An F_1_ female from family G was crossed to an SP15 male to produce the female informative backcross family F2031. Backcross progeny were bioassayed using the discriminatory Bt Cry2Ab concentration [92] to select for homozygous resistant (Cry2Ab r/r) individuals. Additional backcross progeny were not exposed to Cry2Ab, to serve as controls. AFLPs [101] from genomic DNA of grandparents, parents and 59 progeny of family F2301 were analysed for linkage using the method of Heckel *et al*. [18]. Twenty-nine progeny were survivors of exposure to Cry2Ab and 30 were untreated controls. AFLPs were grouped using the program DBM3Lnk.p as in Heckel *et al*. [18]. As expected from achiasmatic oogenesis in female Lepidoptera, no recombinants were found within AFLP linkage groups. Linkage to resistance was tested for each linkage group using signed interaction chi-squared tests with one degree of freedom [102], with a Bonferroni correction for 31 linkage groups.

One AFLP band from the only linkage group with a significant association with resistance was cut out of the gel, reamplified, cloned and sequenced (GenBank Accession No. KJ419919). The insert was hybridized to a Southern blot made from an unrelated Bt-susceptible *H*. *armigera* family in which several ribosomal protein genes had previously been mapped, enabling comparison to the homologous ribosomal protein genes of *Bombyx mori*. This showed AFLP group 8 in family F2301 to correspond to *B*. *mori* chromosome 17 (BmChr17).

Additional linkage mapping in a male-informative backcross (G2016) and two F_2_ families (G2020, G2029) was performed to further localize the resistance locus. Offspring from these families that had survived the discriminating concentration and were presumably homozygous for the SP15-derived resistance allele were examined for recombinants at marker loci. *H*. *armigera* homologs of ribosomal protein genes *RpL22* and *RpS24* on *B*. *mori* BmChr17 were sequenced to identify polymorphisms to be used in mapping. The gene *bre-5* on BmChr17 was considered a candidate for the resistance gene because of its role in Cry4B resistance in the nematode *Caenorhabditis elegans* [19], and was also mapped using sequence variation in the coding region and a PCR-RFLP using a polymorphic *Pst*I restriction site.

### Mapping of the Cry2Ab candidate resistance locus using male-informative cross

To establish an appropriate mapping family, a GR Cry2Ab susceptible homozygous male (Cry2Ab s/s ♂) was mated with a SP15 Cry2Ab resistant homozygous female (Cry2Ab r/r ♀). The F_1_ susceptible heterozygous male (Cry2Ab r/s ♂) was back-crossed to a SP15 female to obtain F_2_ offspring of either homozygous resistant (Cry2Ab r/r) or heterozygous susceptible (Cry2Ab r/s) genotypes in equal proportions. Approximately 300 F_2_ offspring were bioassayed using the discriminatory Bt Cry2Ab concentration [92] to select for homozygous resistant (Cry2Ab r/r) individuals. Control (n = 100) F_2_ offspring were not bioassayed and were included in subsequent genotyping experiments using EPIC-PCR markers as described below.

### Chromosome walk by EPIC markers to ascertain recombination rates

EPIC PCR markers used in this study were designed using the primer designing criteria previously reported by [103] for *H*. *armigera*. Briefly EPIC-PCR primers were designed using the primer analysis software Oligo Version: 7.17 (Molecular Biology Insights, Inc., Cascade, CO 80809, USA) and avoiding false primer annealing sites for both forward and reverse primer, with no or minimal hairpin structures and primer dimmer formation. We also designed the EPIC-PCR primers with intron amplicon of typically less than 500bp such that polymorphisms in F_2_ cross can be easily scored. Intron sizes were estimated based on *B*. *mori* gene annotation. EPIC-PCR primers were optimised prior to having a fluorescent tag (FAM, HEX or TET) attached to the 5’ end of the forward primer. Amplicons of the mapping family from individual EPIC-PCR primer pairs were visualised on 1–1.5% agarose gels prior to being purified by acetic acid/ethanol precipitation and sent to Genetic Analysis Facility (GAF) at James Cook University (JCU) for genotyping. PCR conditions, and genotyping procedures were previously described [103,104]. A list of all EPIC-PCR primers used in this study can be found in S2 Table.

Genomic DNA was extracted using the Qiagen Blood and Tissue extraction kit (Qiagen Cat. #69506). For the founding grandparents (i.e., F_0_) and parents (i.e., F_1_) one leg each was used in gDNA extraction, with gDNA eluted in 200°L of the AE buffer. Bioassayed and control F_2_ samples were collected as 3^rd^ instar larvae and gDNA was extracted as for the parents and grandparents. All genotyping with EPIC-PCR markers involved screening of grandparents, F_1_ parents, 72 bioassayed (Cry2Ab r/r) offspring and 20 control F_2_ offspring (i.e., either Cry2Ab r/r or Cry2Ab r/s). Under the linkage mapping pattern, genome/chromosome walking towards the resistance gene should generate reduced recombination rates in the resistant F_2_ as one approaches the genomic region of interest.

### Identification of candidate genes

Messenger RNA sequencing was done in order to generate full-length transcripts in *H*. *armigera* for candidate genes, identify resistant alleles where cDNA amplification failed and to identify the homologous candidate genes in *H*. *punctigera*. Total RNA was extracted from the midgut of third-instar larvae or whole larvae using the TRIzo Plus RNA purification kit (Life Technologies, Cat # 12183555) and dried down for shipping with RNAstable Tube Kit (Biometrica Cat. # 93221–001). RNAseq library preparation, sequencing and bioinformatic analysis was done according to standard Illumina protocols by the Beijing Genomics Institute (BGI) in Shenzen, China.

Except for the resistant line 7–183 which used gDNA as a template for sequencing, candidate genes from the remaining resistant lines were completely sequenced using a cDNA template from 3^rd^ instar larvae prepared using an RNA extraction kit (Qiagen RNeasy mini kit, Cat. # 74106), and trace genomic DNA contaminants removed using the Qiagen RNase-Free DNase set (Cat. # 79254). First strand cDNA was synthesised using the Invitrogen SuperScript III RT First Strand Synthesis System for RT PCR (Cat. # 18080–051), in the presence of RNase H. All sequencing was performed at the John Curtin School of Medical Research, Australian National University (ANU), and used the ABI BigDye v3 chemistry. Contig assembly used the Staden pregap4 and Gap4 software [105] and was visualised using Artemis (Release 12.0) [106]. Sequences generated and used in this study have been deposited in GenBank (Accession numbers KP259910, KP259911, KP259912).

### Prediction of ABCA2 transmembrane domains

The amino acid sequence predicted from a complete mRNA sequence from a Cry2Ab susceptible individual belonging to the GR-line was used to predict the domain structure of the *H*. *armigera* ABCA2 protein. The protein prediction software Split V3.5 <http://split4.pmfst.hr/split/4/> [107] was used to search for transmembrane protein secondary structure (i.e., transmembrane helices). In the mouse RmP ABC transporter, several N-glycosylation sites were predicted on the protein’s extracellular domains [76,108]. We used the NetNGlyc 1.0 Server <http://www.cbs.dtu.dk/services/NetNGlyc/> developed to predict N-Glycosylation sites in human proteins for the purpose of predicting N-Glycosylation sites in the protein sequences. The software uses artificial neural networks to examine for Asn-Xaa-Ser/Thr sequence context.

### Identification of sequences and phylogenetic analyses

Sequences in the transcriptome databases corresponding to candidate genes were identified by standalone BLAST. Homologs in GenBank were identified using BLAST and homologous gene clusters identified in NCBI and in Ensembl. Orthologous genes from other lepidopteran genomes were retrieved using their online databases from public domains as cited in the appropriate sections below. Protein sequences were aligned using Multiple Alignment using Fast Fourier Transform (MAFFT) [109] <http://www.ebi.ac.uk/Tools/msa/mafft/> and phylogenetic tree (maximum likelihood (ML) with rapid bootstrapping) inference using RAxML-HPC2 on XSEDE (8.0.24) (available at the CIPRES Science Gateway V3.3) <http://www.phylo.org/sub_sections/portal/> [110–112], and redrawn using Dendroscope version 2.4 [113].

## Supporting Information

S1 Table Table of genes on *Bombyx mori* chromosome 17 analysed during mapping the resistance locus. Column 1 lists the markers used, column 2 the identifiers for the NCBI-predicted gene models, column 3 the BGI gene models, column 4 and 5 the start and end of the genes on the Chr17 sequence, column 6 the Microarray Database gene identifier and column 7 the highest expression level observed in the midgut [114]; see <http://www.silkdb.org/microarray/>. For columns 4 and 5, the coordinates were determined by using Scipio [72] to map proteins against the chromosome sequence.
(XLS)

S2 Table EPIC-PCR markers used in chromosome walk to estimate recombination rates in *Helicoverpa armigera* F_0_ (grandparents), F_1_ (parents) and F_2_ (bioassayed progenies) using the Bt toxin Cry2Ab. Gene names, amplicon size (range in base pairs, bp) and PCR annealing temperatures are also provided. Estimated chromosome positions of markers based on *Bombyx mori* chromosome 17 (BmChr17) are provided in S1 Table. IUPAC codes for ambiguous base pairs are: Y (T/C), W (T/A), K (G/T).
(DOCX)

S1 Fig MAFFT alignment of ABCA1 and ABCA2 protein sequences from Lepidoptera, with *Drosophila* homolog. GenBank Accession Numbers are as follows: *Helicoverpa armigera* HarmABCA1 (GenBank: KP259910), HarmABCA2 (GenBank: KP259911); *Heliothis virescens* HvirABCA1 (KP219764), HvirABCA2 (KP219765); *Bombyx mori* BmorABCA1 (KP219766), BmorABCA2 (KP219767); *Plutella xylostella* PxylABCA1 (KP219762), PxylABCA2 (KP219763); *Danaus plexippus* DpleABCA1 (KP219768 and KP219769), DpleABCA2 (EHJ70360); *Heliconius melpomene* HmelABCA1 (HMEL005382-PA, http://www.butterflygenome.org/), HmelABCA2 (KP219770); *Drosophila melanogaster* Dm_CG1718 (NP_001259765). Sequences are complete except for Dplex ABCA1, due to a gap in the genome assembly for which no transcriptomic data were available.
(DOCX)

S2 Fig Sequence alignment of the *Helicoverpa armigera* ABCA2 gene exons from Bt Cry2Ab toxin susceptible individuals (GR), resistant alleles Ha2Ab-R01 (SP15), Ha2Ab-R02 (6–364) and Ha2Ab-R03 (6–798). Nucleotide variation (highlighted in orange) and deletions (‘-’ highlighted in green) are shown. The 8bp partial intron sequence (GGTTAAG) detected in the Ha2Ab-R01 allele is shown within the 72bp deletion (nt2892-2962) on exon 16. The 5bp deletion (ACAAG) of Ha2Ab-R02 is at nt3127-3131 on exon 18. The 5p insertion (GAATA) of Ha2Ab-R03 allele is between nt410-4104 (exon 24). Amino acid substitutions involving molecules with similar (hydrophobic) side chain are in red boxes, while substitutions between different category amino acids are in blue boxes. Amino acid symbols are: L (leucine), M (methionine), V (valine), I (isoleucine), Y (tyrosine), F (phenylalanine), A (alanine), P (proline), G (glycine), E (glutamic acid), K (lysine), and T (threonine).
(PDF)
