# Supplementary material for: Insect Resistance to Bacillus thuringiensis Toxin Cry2Ab Is Conferred by Mutations in an ABC Transporter Subfamily A Protein
Source: PLoS Genet. 2015 Nov 19;11(11):e1005534. doi: 10.1371/journal.pgen.1005534 (PMC4652872; doi:10.1371/journal.pgen.1005534)
Supplement: S2 Table — Gene names, amplicon size (range in base pairs, bp) and PCR annealing temperatures are also provided. Estimated chromosome positions of markers based on Bombyx mori chromosome 17 (BmChr17) are provided in S1 Table. IUPAC codes for ambiguous base pairs are: Y (T/C), W (T/A), K (G/T). (DOCX) [file pgen.1005534.s002.docx]

**S2 Table:** EPIC-PCR markers used in chromosome walk to estimate recombination rates in *Helicoverpa* *armigera* F_0_ (grandparents), F_1_ (parents) and F_2_ (bioassayed progenies) using the Bt toxin Cry2Ab. Gene names, amplicon size (range in base pairs, bp) and PCR annealing temperatures are also provided. Estimated chromosome positions of markers based on *Bombyx mori* chromosome 17 (BmChr17) are provided in S2 Table. IUPAC codes for ambiguous base pairs are: Y (T/C), W (T/A), K (G/T).

| **Gene Name** | **Gene/Primer name** | **Forward** | **Reverse** | **Amplicon (bp)** | **Annealing Temp (˚C)** |
| --- | --- | --- | --- | --- | --- |
| Ribosomal Protein gene Large subunit 38 | RpL38 | CTAAAGGCACGCAGGAAAGATG | TGAGCATCGCACTTTGAACTTC | 145-183 | 52 |
|  |  |  |  |  |  |
| Transmembrane channel-like protein 3-like | TCGF5 | GACCTATCTCGATGTGGAAACG | CTCGAAGGAGAAGACCACGTC | 394-607 | 56 |
|  |  |  |  |  |  |
| Beta-eliminating lyase | BetaEL | GTCCACGAACCTATCACCTCAT | ACCTTGCCCCCACATACATTG | 426-470 | 54 |
|  |  |  |  |  |  |
| Myosin heavy chain, non-muscle-like | Zip2 | CACGCTGCCTGATAACACTGTC | CTACTTGCTCCTTGGTCTGTGC | 210-230 | 52 |
|  |  |  |  |  |  |
| WD repeat-containing protein 81-like | BEACH | CGAAATAGATACCTCGCTAAACA | TGCCTTCATTATTCTGACAGTAC | 316-367 | 52 |
|  |  |  |  |  |  |
| Usher syndrome type-1G protein homolog | ANK_SAM | CGAACGACCTGGCTTTGGTAG | GCTGTTATTGAACGTCTGGAAAT | 407-453 | 52 |
|  |  |  |  |  |  |
| Putative inorganic phosphate cotransporter-like | NaPT | GACTGTCCTAACGCCTTTTGCT | CTCCTCCAAATATCATGGCACC | 425-545 | 52 |
|  |  |  |  |  |  |
| Golgi to ER traffic protein 4 homolog | DUF410 | GATCCCTTTTAATACTTATTGCAT | GATCCTTTGCCTGCGTAATATA | 285-315 | 50 |
|  |  |  |  |  |  |
| ABC transporter sub-family A member 1 | ABCA1 | GTGAATACGGTCAGGGTTGTAA | TGATAAACCATGCCAGCCAGTG | 273-282 | 52 |
|  |  |  |  |  |  |
| Chorion peroxidase-like | AN_Peroxidase | CTCGGCTACCAAGTCTGATAAG | GGACGACGTTGTTGCATTCTG | 312-381 | 54 |
|  |  |  |  |  |  |
| Voltage-Gated Calcium Channel alpha subunit cav 3.3 | VGCal-A | ACGACGGTACAGATTCAAAGACC | CGAACCAGCCCCGCGTCACC | 515-522 | 50 |
|  |  |  |  |  |  |
| Ribosomal Protein gene Large subunit 22 | RpL22 | AAAWWTTGAYACAACAAAATGTCT | TGTGTTGTCKGCGCAGTTGA | 275-330 | 52 |
|  |  |  |  |  |  |
| ABC transporter sub-family A member 2 | ABCA2-F6/R5 | TCCTTCTTCTCTTTGGTATTTGG | GCTCCACATTTGACGACGACTC | ~2,100  (cDNA) | 50 |
|  | ABCA2-F4/R4 | GTACTGCATCCGAGTTTTTAGATC | CTCAGCTTCAACGGCAGTGTAG | 311, 316 (cDNA) | 50 |
|  |  |  |  |  |  |
|  | ABCA2-F3/R3a | CAGAATTGGCGAACAAGCTTTGG | CAAGTGCTGGCTGTATGGATGAA | 209, 275 (cDNA) | 50 |
|  |  |  |  |  |  |
|  | ABCA2-F3/R3 | CAGAATTGGCGAACAAGCTTTGG | TAACTCGCGGGACAGTGAACGT | ~1,300  (cDNA) | 52 |
|  |  |  |  |  |  |
|  | ABCA2-F2/R2 | TCAATAAGTGAGTCTATCTCGTCAT | GAAACTATGAAAATTATGGGTCTCC | ~1,100  (cDNA) | 54 |
|  |  |  |  |  |  |
|  | ABCA2-F1/R1 | GTGATTGATAGGTAGATAAACTG | ATGACGCAGGAGGAGGACGAT | ~1,100 | 50 |

**Note:** The primer ABCA2-F6 was used to generate single strand cDNA in RT-PCR from RNA (expected amplicon size >3,000bp); ABCA2-F6/R5 amplified exons 18-31 (~1,100bp) from cDNA template, ABCA2-F3/R3a and ABCA2-F4/R4 are primers used to characterise the *H. armigera* Bt Cry2Ab resistance allele 1 (Ha2Ab-R01) and allele 2 (Ha2Ab-R02) (between exons 16-18); primers ABCA2-F3/R3 amplified exons 11-17; primers ABCA2-F2/R2 amplified exons 5-11; primers ABCA2-F1/R1 amplifies exons 1-5.
