# Supplementary material for: Insect Resistance to Bacillus thuringiensis Toxin Cry2Ab Is Conferred by Mutations in an ABC Transporter Subfamily A Protein
Source: PLoS Genet. 2015 Nov 19;11(11):e1005534. doi: 10.1371/journal.pgen.1005534 (PMC4652872; doi:10.1371/journal.pgen.1005534)
Supplement: S1 Fig — GenBank Accession Numbers are as follows: Helicoverpa armigera HarmABCA1 (GenBank: KP259910), HarmABCA2 (GenBank: KP259911); Heliothis virescens HvirABCA1 (KP219764), HvirABCA2 (KP219765); Bombyx mori BmorABCA1 (KP219766), BmorABCA2 (KP219767); Plutella xylostella PxylABCA1 (KP219762), PxylABCA2 (KP219763); Danaus plexippus DpleABCA1 (KP219768 and KP219769), DpleABCA2 (EHJ70360); Heliconius melpomene HmelABCA1 (HMEL005382-PA, http://www.butterflygenome.org/), HmelABCA2 (KP219770); Drosophila melanogaster Dm_CG1718 (NP_001259765). Sequences are complete except for Dplex ABCA1, due to a gap in the genome assembly for which no transcriptomic data were available. (DOCX) [file pgen.1005534.s003.docx]

**S1 Fig:** MAFFT alignment of ABCA1 and ABCA2 protein sequences from Lepidoptera, with *Drosophila* homolog. GenBank Accession Numbers are as follows: *Helicoverpa* *armigera* HarmABCA1 (GenBank: KP259910), HarmABCA2 (GenBank: KP259911); *Heliothis* *virescens* HvirABCA1 (KP219764), HvirABCA2 (KP219765); *Bombyx* *mori* BmorABCA1 (KP219766), BmorABCA2 (KP219767); *Plutella* *xylostella* PxylABCA1 (KP219762), PxylABCA2 (KP219763); *Danaus* *plexippus* DpleABCA1 (KP219768 and KP219769), DpleABCA2 (EHJ70360); *Heliconius* *melpomene* HmelABCA1 (HMEL005382-PA, http://www.butterflygenome.org/), HmelABCA2 (KP219770); *Drosophila* *melanogaster* Dm_CG1718 (NP_001259765). Sequences are complete except for Dplex ABCA1, due to a gap in the genome assembly for which no transcriptomic data were available.

BmorABCA1 M---------SAVAKLKLLIWKNVLLQKRHKWQTIFEIASPVIFSLFLILTRCLVDPKSK

HarmABCA1 M---------SAFEKLKLLIWKNFLLQRRHKWQTIFEIASPVIFSLFLILTRCLVDPKSK

HvirABCA1 M---------SAYEKLKLLIWKNFLLQRRHKWQTIFEIASPVIFSLFLILTRCLVDPKSK

HmelABCA1 M---------SSFDKLKLLIWKNFLLQRRHKWQTLFEIASPVIFSLFLILIRCLVDPQSK

DpleABCA1 M---------SAFEKLKLLIWKNFLLQKRHKYQTLFEIASPVIFSLFLILIRCLVDPQSK

PxylABCA1 M----------YWKKLKLLVWKNFLLQKRHKWQTIFEIASPVIFAMFLILIRSLVAPKSK

BmorABCA2 MRP--QRKEAGAFTKFRLLIWKNLIQQWRHRLQTVVELLLPVITMTLILILRWQVEPTNR

HarmABCA2 MRL--ETRHASAATKFRLLMWKNFLQQWRHRLQTVVELFLPVVTMALVLILRWQIPPYQI

HvirABCA2 MRL--EARHASAATKFRLLMWKNFLQQWRHRIQTVVELLLPVVTMTLVLILRWQIPPSQI

HmelABCA2 MKVTKEFKEASSWMKFRLLMWKNFLQQWRHRKQTLIELLLPVVTMTLVLILRQQIEPVKQ

DpleABCA2 MRNY----DASWWTKFRLLMWKNFLQQWRHRIQTVVEMLLPVATMALILILRHQIEPIRQ

PxylABCA2 MTS--AQREASAFTKFRLLMWKNFLIQWRHPAQTVAELLVPVLAMSLVMVLRSQIMPSME

Dm_CG1718 M------AKVTNWDKFVLLLWKNWTLQWNHKWQMVIELVLPAIFSLLLVLVRTLVDTEQK

* *: **:*** * .* * : *: *. :::: * : .

BmorABCA1 PAITYSPFQPTYLNITGRNLGNLTAAKT-GTLAFSPENPLTRNVVRDAIAMVANANLSFL

HarmABCA1 PDISYPPFLPTYFNISGRNLGNLTTAKT-GTLAFSPENPLTRNVTRDAIAMVADDNFSIL

HvirABCA1 PDLSYPPFLPTYFNISGRNLGNLTTAKT-GTLAFSPENPLTRNVTRDAIAMVADDNFSIL

HmelABCA1 PDVSYPPFLPTYFNISGRQLGNLTSAKREGTLAFSPENPLTRKVTEDAMAKVALDNLNGF

DpleABCA1 PDTSYQPFLPTYFNMSGRQLGNLTTAKSDGTLAFSPENALTRKVTKDAMAKVALDNLNGF

PxylABCA1 PGMNYESFQPTYFNISGRSFGNLTTARREGTLAFSPESPLTRTLTKKAIETVVLDNVNGF

BmorABCA2 ETIRYPEISSHSLQYSSMIIAGLNTTRM--SIAYSPTSPELEDVVRSSVANLLLLNIKHI

HarmABCA2 DTLTYPALPAHTLNYSTNILYAMNMEEL--SIAYSPASPVLDDVMRTAVINLLTANMKDL

HvirABCA2 ETLTYPSLPAHTLNFSTTILFAMNMEEL--AIAYSPASPVLDDVMRTAVVNLLVANMKDL

HmelABCA2 GTITYPPIPAYSLNFSREVIATLDFREL--SLAYSPQSPILDDVVRSAIVNLFALNLRDL

DpleABCA2 DTVVYPPVPAYSLKFSTTVLAGLNISKL--SVAYSPESPVLETVIQNALVNLFTPNIKDL

PxylABCA2 DRVDYPPFPAHTLQYSSVVLAGMNVTRM--SIAYSPTSPVLQDVVQMASLKLLVNNYKVL

Dm_CG1718 GVRYYNEQNLTDLNLLQKNGG---FSKFEFILCYSPVNPVLKKLVEEAWQSLGKNKIC--

* :: . :.:** .. : . : : :

BmorABCA1 FTFIFDSN-----------------------------------ILPQPKGYKNAKDMELA

HarmABCA1 FALLFDSN-----------------------------------FLPQPKGYKNAQEMELA

HvirABCA1 FALLFDSN-----------------------------------FLPQPKGYKNAQEMELA

HmelABCA1 IALFFDTA-----------------------------------MLPTPEGYNNSTALEAA

DpleABCA1 IALLFDPR-----------------------------------VLPEPKGFNDSSELEAA

PxylABCA1 LALLFDVS-----------------------------------MLPEPKGYASASDMEMA

BmorABCA2 ----INDT--ETIMPEV--ELPPDINWN------DSAIYEIIKRILRVDAYDNSNALRGI

HarmABCA2 IPIFIDNL--PPGIANI--TFPPDMNLN------TSVIEEFVKSRIRVVPYNSSYEIRGI

HvirABCA2 IPIFIDNL--PPGMPDI--TFPPDIDFN------ATAITDFVKSRIRVVPYNSSYEIRGI

HmelABCA2 ISI-IAEQ--DINIPDISIEIPEDL--N------TTIIYEIAKRLVRITPYNSSSQLRSI

DpleABCA2 IQI-IKDNWGEGGLPS---PIPPNVINN------PAIIGDIVKLLIRIQPYNNSRALETV

PxylABCA2 LPLLIEQL--ENFIP----GFDPGILENLDPTQLPDDVLDLLKILVNSVGYENSAELKGI

Dm_CG1718 -------------------------------------------------ESENATQLEL-

.: :.

BmorABCA1 LTQPNAMNQILVGIQFDDVMAN---ATE---WPENITVRFRFPAVMRTPMIEHPLRASWR

HarmABCA1 LTQPNAMNQILVGIQFEDSMAN---ATE---WPDDVTLTLRFPAVMRTPMVEHPLRASWR

HvirABCA1 LTQPNAMNQILVGIQFEDSMAN---ATE---WPDDVTLTLRFPAVMRTPMVEHPLRASWR

HmelABCA1 LTQPNVMNHILVG-YVSNYIID---ATE---WPDDIKVTLRFPAVMRTPMLEHPLRISWR

DpleABCA1 LSKPNVMNHILVGIQFDDSMAN---ATE---WPEDINVTLRFPAVMRTPMLEHPLRISWR

PxylABCA1 LKEPNVMNHVLCGIQFEDSMAK---ATE---WPDDVVVTLRFPAVMRTPMVEHPLRASWR

BmorABCA2 YALEEITREVVLAVEFNDSLLG---ATE---LSNNLSFALRFPERPRLNSFYAQGGRSWR

HarmABCA2 YVDEETTRSIIAAVEFDDKLYG---AEQ---LSNNLSYSLRFPERPRLNSFFQTGGRTWR

HvirABCA2 YVDEETTRSIIAAVEFDDKLYG---AEE---LSNNLSYSLRFPERPRLNSFFQTGGRTWR

HmelABCA2 YGNEQDIRRVIAAIEFDDSLLG---TTQ---LPNNVSYAIRFPERPRLNSILGIGGRNWR

DpleABCA2 YSEEKAIREVIAAVQFDNDLLGLDDSRK---VPFNMTYFLRFPEKPRQYSLFGIGGNSWR

PxylABCA2 YRNEESTRTVIAAVEFPDELLG---ATT---IPLNMSYALRFPEKPRLNSFFGQGGRSWR

Dm_CG1718 ---DTVSKNAFAGVQFDDAWAN---LTENDPLPNDFHFALRFPAELRTATI--AIANTWL

. . . . : . :. :*** * . .*

BmorABCA1 TNLLFPLFPRPGPRDADDMYGGKTPGWISPEMFLAVQHAVSQEIIKQKTG-KAI-NTKVY

HarmABCA1 TNLLYPLFPRPGPRDPDDMYGGKTPGY-SPEMFLAVQHAISQEIIKQKTG-KPI-NTKVY

HvirABCA1 TNLLYPLFPRPGPRDPDDMYGGKTPGY-SPEMFLAVQHAISQEIIKQKTG-KPI-NTKVY

HmelABCA1 TNLLFPLFPMPGPREPNDLYGGKMPGY-SPEMFLAVQHAVSQEIIKQKTG-KSI-NTKVY

DpleABCA1 TNLLFPLFPQPGPRVPKDMYGGKTPGY-SPEMFLAVQHAVSQEIIKQKTG-KSI-NTKVY

PxylABCA1 TNLLLPLFARPGPRDPDDLYGGKTPGY-SGEMFLAVQHAVSIEIIKAKSG-QPL-ETKVL

BmorABCA2 TDDVFPVFENPGPRFPLSWEGGNDPGY-VNEMFIAFQSCISAELVSRLTGKPELKSFSVN

HarmABCA2 SDGVFPVFEVPGPRFPHSWEGGNDPGY-VNEMFVALQQVISTELVSRATG-LDLKSFRVN

HvirABCA2 SDVVFPVFEVPGPRFPYSWEGGNDPGY-VNEMFVALQHVISTELVSRATG-LDLKSFKVN

HmelABCA2 TGNTFPLFELPGPRFPYSWEGGNDPGY-VNELFIAIQQSISMELISRFAG-EKLNAFTVN

DpleABCA2 TDEIFPFFEVPGPRFPFSWEGGNDPGY-VNELFIGFQHAISMELVELMTG-QSLKDFTVH

PxylABCA2 TTLVFPTYPTPGPRFPYSYEGGNDPGY-VNEMFIALQQCISMELISRMTG-QSL-DLDVK

Dm_CG1718 TMRLFPTIDLTGPRNEGDDDGGIPPGY-LREGFLPLQHSLSMAYLRQKSG-EQD-LPNVV

: * .*** . ** **: * *: .* :* : :* *

BmorABCA1 LQRFPQVAYREDELLIALERFISMIIMLCFAYTFVNTVKMVTNEKELQLKETMIIMGLPS

HarmABCA1 LQRLPQLAYREDQLLVALERFISMIIMLCFAYSFVNTVRVVTFEKELQLKETMTIMGLPS

HvirABCA1 LQRLPQLAYREDQLLVALERFISMIIMLCFAYSFVNTVRVVTFEKELQLKETMTIMGLPS

HmelABCA1 LQRLPQLSYRQDDLLVAMERFISMIIMMCFAYTFVNTVRVVTAEKELQLKETMTIMGLPS

DpleABCA1 LQRLPQLSYRQDDLLVAMERFISMIIMMCFAYTFVNTVRVVTAEKEMQLKETMTIMGLPS

PxylABCA1 LQRMPQLPYTEDLLLLAMELFISMIIMLCFAYTFVNTVKVVTAEKELQLKETMTIMGLPS

BmorABCA2 IQRYPHPPYVDDLAVEALQMIFPMFILLSFSYSAVNLVRAVTLEKELQLKETMKIMGIST

HarmABCA2 IQRYPHPPYLHDQSVDLLQFMFPLFIMLSFSYTSVNIARAVTVEKELQLKETMKIMGLPT

HvirABCA2 IQRYPHPPYLQDQSVDLLQFMFPLFIMLSFSYTAVNIARAVTVEKELQLKETMKIMGLPT

HmelABCA2 LQRYPHPPYVEDLAVEALIFIFPMFFMLSFSYTAVNIVRSVTVEKELQLKETMKIMGLNT

DpleABCA2 INRYPHPPYVQDLAVEALMYIFPMFIMLSFSYTAVNIIRTITIEKELQLKETMKIMGLPT

PxylABCA2 IQRFPHPPYTLDGAIQALLLLFPMFFILSFSYTAVNLVRAVTVEKESQLKEAMKIMGLPT

Dm_CG1718 MKRYPFPAYIFDPLLEGMSSIMSLIILLSFIYPCTYITKYITAEKEKQLKEVMKIMGLSN

::* * .* * : : ::.:::::.* *. . : :* *** ****.* ***: .

BmorABCA1 WLHWLAWFIKQFSFLLISVLLIVILLKIPFNHTEDG-QGYSVLTFTPWSVLFFFMVLYVM

HarmABCA1 WLHWLAWFIKQFSFLLISVGLIVILFKIPFNRTAAG-DGYSVLTFTPWSVLFFFMILFVI

HvirABCA1 WLHWLAWFIKQFSFLLISVVLIVILFKIPFNRTAAG-DGYSVLTFTPWSVLFFFMILFVI

HmelABCA1 WLHWLAWFVKQFSFLLISVILMVILFKIPFNSTQDG-EGYAVLTFTPWTVLFFFLVLFVV

DpleABCA1 WLHWLAWFIKQFSFLLISVILMVILFKIPFNSTSDG-EGYAVLTFTPWSVLFFFLILFVI

PxylABCA1 WLHWLAWFIKQITYLSISVLLLVILLKIPIKSSDTG-ERFSVMTHTPWSILIFFLFLFIV

BmorABCA2 WLHWTAWFFKQFIYLMISAILIIVLLKVSWFTNADGFSGYAVFTNTPWTVLLFFLALYLI

HarmABCA2 WLHWTAWFVKQFIYLSITAVLLVVLLKVNWFTNDDGFSEYAVFTNTPWTVLLFFLILYLS

HvirABCA2 WLHWTAWFVKQFIYLTIAAILLVVLLKVNWFTNEDGFSEYAVFTNTPWTVLLFYLILYLS

HmelABCA2 WLHWTAWFFKQMIYMFIITILIVIILKINWFTTEQGYSGYAVFTNTPWTVLLFYITLYLS

DpleABCA2 WLHWMAWFWKQFIYLFVTGLLITVILKVNWFTNEQGFSGYSVFTKTPWTVILLFIMLYLS

PxylABCA2 WLHWTAWFCKQFLFLLVIAVLIVLLLKINWFTTPEGFSEFAVFTHTPWSVLLFFTLLYLV

Dm_CG1718 WLHWTAWFVKSFIMLTISAILIAILVKINWS------EDVAVLTHANFTALVFFLIIYIV

**** *** *.: : : *: ::.*: . :*:* : :: :.:: :::

BmorABCA1 ASLAFSFMISVFFNKANTASSFMGLAWFSTYAVFMLTQVLYEDISLTTKLLLSLISNTAM

HarmABCA1 ASLAFSFMISVFFSRANTAASFMGLAWFAAYSIYMLTQVLYEDISLSTKLLLSLISNTAM

HvirABCA1 ASLAFSFMISVFFSRANTAASFMGLAWFAAYSIYMLTQVLYEDISLSTKLLLSLISNTAM

HmelABCA1 ASLSFCFMVSVFFTRANTAASFMGLAWFSTYAAFMLTQMLYEDISLTTKLLLSLISNTAV

DpleABCA1 ASLSFCFMVSVFFTRANTAASFMGLAWFSTYSAYMLTQMLYEDISLTTKLLLSLISNTAI

PxylABCA1 TSLAFSFMVSVFFARANTAASFMGLAWFATYSVFMLTQVLYEDISLTTKVLLSLISNTAL

BmorABCA2 CSIFFCFMISGFFSKGSTSALFGGVIWVLVYIPALLLSM-DNNMSIVTQVITCLSVNCAM

HarmABCA2 CAIFFSFMVSSIFSKGSTAALFMAVAWFLTYIPAFLLAM-DINMSTAVQVITCFSINSAM

HvirABCA2 CAIFFSFMVSSIFSKGSTAALFMAVAWFLTYIPAFLLAM-DFNMSTAVQVITCFSINSAM

HmelABCA2 CTIFFCFMISGFFSKASTAALFGGVIWFMSFIPAVLLGI-DVEVPLAVQAISCISINSAM

DpleABCA2 CTIFFCFMISSFFSKGSVSALFGGVIWFITFIPAFLLGM-DVQVSLPVQAITCLSINSAM

PxylABCA2 CVIFLCFMVSSFFAKASTAALFMGVTWFILYIPAVLLSM-DESVSLAAQVLACLSVNTAM

Dm_CG1718 SSICFCFMMATFFSRASTAAAVTGLIWFIAYIPYSFTINSYDDLSLSSKLGWSLISNTAM

: :.**:: :* :...:: . .: *. : : .:. : .: * *:

BmorABCA1 GYAFQMIIMCEGTSRGLQWNEFF-SPISYHDKFQPGHVMLMLMLDTILYMLIAMYVEKIR

HarmABCA1 GYAFQMIIMCEGTSKGLQWNEFF-TPISYQDQLQPGHIVLMLILDSILYMLIAMYVEKIR

HvirABCA1 GYAFQMIIMCEGTSKGLQWNEFF-TPISYQDQLQPGHIVLMLILDSILYMLIAMYVEKIR

HmelABCA1 GYALQMLIICEGTSRGLQWNEFF-TPVSYHDQFQPGHVALMLILDSILYMLVAMYVEKIR

DpleABCA1 GYALQMLVVCEGTSRGLQWDEFF-MPVSYHDQFQPGHVALMLVLDSILYMLIAVYVEKIR

PxylABCA1 GFAFQMLIMCEGKS-GLQWSNMF-KPVSYHDSFQPGHVALMFVLDTILYMMIAMYVEKIR

BmorABCA2 SYGFQLLFGKESIG-GMQWGDFMASPSSDSSRFVFGHVILMLVFDSVLYMLIALYLEQVL

HarmABCA2 SYGFQLMLAKESTG-GLQWGDFMTSPETDTTRLVFGHVVIMLVVDCLIYMLITLYLEQVL

HvirABCA2 SYGFQLMLAKESTG-GLQWGDFMTSPGTDTSRFVFGHVVIMLVVDCIIYMLITLYLEQVL

HmelABCA2 SFGFQLILGKENSE-GMQWGEFFATHSVDSNRLLFGHICIFLIFDSVLYMLIALYLEQVL

DpleABCA2 SYGFQLILGAEGSQ-GMHWGEFFATHSIETDRLLFGHVCLILLLDCFLYMLLALYFEQVL

PxylABCA2 SYAFQLMITEESNG-GLQWGKFFFTPAADEPRLLFGHVVLMIVLDCALYLLVALYCEQVM

Dm_CG1718 GFGIKLILGFEGTGEGLQWSNFF-TPVSVDDTLTLGAVMIMMLVSCVIYMIICLYVEQVM

.:.::::. *. *::*..:: : * : ::::.. :*::: :* *::

BmorABCA1 PGKFGVPMPWYFPFTKKFWSTNKSR----------IAAAK-TEESSDTAYHDALLKVV--

HarmABCA1 PGLYGVPLPWYFPFTKSFWRPNKSK----------MAEIN--KDTRDMEYNDALLKVVHD

HvirABCA1 PGLYGVPLPWYFPFTKNFWRPNKSK----------IAEIN-KKDTRDMEYNDALLKVVHD

HmelABCA1 PGMYGVPLPWYFPFTKRFWSPDKTKVAVYVKFNINVAGVT-NEHVVDQEYKNALLKVV--

DpleABCA1 PGLYGVPLPWYFPFTKSFWCPDNTK----------VAALT-NKDGVDQEYKNALLKVI--

PxylABCA1 PGRYGVPLPWYFPVTKQFWSSKKSR----------IAALNRSTTAADAEYNDALLKVV--

BmorABCA2 PGPFGLPKPWYFPIQKSFWFPNNEE----------SKNGI-IIEHAISN-DD----VI--

HarmABCA2 PGPFGTPKPWYFPFQLQFWFPNYKS----------KDAGL-IFENDNSEFDD----II--

HvirABCA2 PGPFGTPKPWYFPFQLQFWFPNYKS----------KDAGL-IFENDNSEFDD----II--

HmelABCA2 PGPYGAPKPWYFPFQKSFWCSSRKV----------VYDDT-LF--GATDTYE----TI--

DpleABCA2 PGPCGTARPWYFPFQKSFWFPSKQI----------NHNYE-AY--NNPEYNI----AI--

PxylABCA2 PGPFGTARPWYFPFTREFWWSDGKA----------SDGVV-IVDETQ---HD----VT--

Dm_CG1718 PGSFGVPRPWNFPFTREFWCGEREY----------TGVED-IPNGHVEQRDP----KA--

** * . ** **. ** .

BmorABCA1 HDEEPKDSPVGVDIQNLTKVYKGRKAAVDNLTLRLYENEITVLLGHNGAGKTTTISMLIG

HarmABCA1 HDEEPKGVPMGVNIQNLTKIYKGRKKVVDNLNLRMYENEITVLLGHNGAGKTTTISMLTG

HvirABCA1 HDEEPKGVPMGVNIQNLTKIYKGRKKVVDGLNLRMYENEITVLLGHNGAGKTTTISMLTG

HmelABCA1 HDEEPKGVPMGINIENLTKIYKGRKKAVDNLNLRMYENEITVVLGHNGAGKTTTISMLTG

DpleABCA1 HDEEPKGIPMGINIENLTKVYKGRKKAVDNLNLRMYENEITVLLGHNGAGKTTTISMLTG

PxylABCA1 HDEEPQGVPMGIQATNLTKIYRGGRKAVDNLNLKVYENEITVLLGHNGAGKTTTISMLTG

BmorABCA2 LEKDPENLTVGVRMNNLTKIF-GANVAVNQLTLNIFDNQITVLLGHNGAGKSTTMSMLTG

HarmABCA2 KEKDPTDHEVGVRMQNLTKIF-GNNIAVNNLSLNIYDDQITVLLGHNGAGKSTTISMLTG

HvirABCA2 KEKDPTDHEVGVKMHNLTKIF-GNNIAVNNLSLNIYDDQITVLLGHNGAGKSTTISMLTG

HmelABCA2 KEKDPMDLDVGVKMINLTKVY-GNNVAVNNLNLNIFDDQITVLLGHNGAGKSTTISMLTG

DpleABCA2 KEKDPTNLKVGVKMANLTKMY-GRNLVVDNLCLNIYDDQITVLLGHNGAGKSTTISMLTG

PxylABCA2 KEEDPKDYTAGVKISNLTKVY-GAIVAVNNLSLNIFNDQITVLLGHNGAGKSTTISMLTG

Dm_CG1718 FETEPEGKHIGLQMRHLKKRF-GNKMVVKGLSMNMFEDEITVLLGHNGAGKTTTISMLTG

: :* . *: :*.* : * .*. * :.:::::***:********:**:*** *

BmorABCA1 MIPPTSGTATISGYNIVTETEMARSSIGICPQHNVLFPDLTVAEHIEFYARLKGVSNNEI

HarmABCA1 MVPPTSGKAFINGYNIVTETGLARKSLGICPQHNVLFPDLTVAEHIIFYSRLKGVPNSKL

HvirABCA1 MVPPTSGKAFINGYNIVTETGLARKSLGICPQHNVLFPDLTVAEHIIFYSRLKGVPNSKL

HmelABCA1 MVPPTSGSASINGYDIVTETKQARKSIGICPQHNVLFPDLTVAEHIIFYSRLKGVHKSKI

DpleABCA1 MVPPSSGSATINGYDITRETEQARRSIGICPQHNVLFPDLTVAEHIIFYSRLKGVPSSKL

PxylABCA1 TIPPTSGTATINGCDIVTDTGLARASLGICPQHNVLFPDLTAAEHVQFYAQLKGVRGGAV

BmorABCA2 NMKATRGSVTLAGYDIQTQVKAARAHLGFCPQHNVLFNDLTVKEHLEFFARLKGFSGKEL

HarmABCA2 NLKLTRGTVNVAGYDMTSQSSAARSHIGLCPQHNILFNELTVKEHLEFFARLKGFKGKEL

HvirABCA2 NLKLTRGTVNVAGYDMTSQSSAARSHIGLCPQHNILFNELTVKEHLEFFARLKGFKGKEL

HmelABCA2 NVEITQGNVWVAGYNMSTQTRLARSHLGLCPQHNVVFNELTVREHLEFFARLKGYSGDDL

DpleABCA2 NVEVTRGNVWVAGYDMTTQTQLGRAHIGLCPQHNVLFNELTVREHLEFFARLKGYSGQQL

PxylABCA2 NVSATSGSIWLAGYDIAHQLKAARAQVGLCPQHNVLFSELTVREHLEFFGRLKGLSGEGL

Dm_CG1718 MFPPTSGTAIINGSDIRTNIEGARMSLGICPQHNVLFDEMSVSNHIRFFSRMKGLRGKAV

. : *. : * :: : .* :*:*****::* :::. :*: *:.::** :

BmorABCA1 QKEVDHFVKLLELEEKRHAAASSLSGGQKRRLSAGCALCGRSRVVLLDEPTSGLDPPARR

HarmABCA1 KEEVNHFVKLLELEAKRDVTSNKLSGGQKRRLSVGAAMCGNSRVVLLDEPTSGLDPAARR

HvirABCA1 KEEVNHFVKLLELEAKRDVTSNKLSGGQKRRLSVGAAMCGNSRVVLLDEPTSGLDPAARR

HmelABCA1 AEEVDHFIKLLELEEKRDVISKHLSGGQKRRLSVGAAMCGSSRVVLLDEPTSGLDPAARR

DpleABCA1 QAEVDHFVKLLELEEKRNVISKHLSGGQKRRLSVGAAMCGSSRVVLLDEPTSGLDPAARR

PxylABCA1 RGEVDHFLKLLDLEQKADVLSKHLSGGQKRRLSVGVAMCGGSRVVLLDEPTSGLDPAARR

BmorABCA2 YQEIDSLIEKLELQEKQNYVANGLSGGQKRRLCVGIALSGAASVVLLDEPTSGMDPASRR

HarmABCA2 YDEIDSLIEKLELQEKRDYPSKGLSGGQKRRLCVGIALSGAARVVLLDEPTSGMDPSSRR

HvirABCA2 YDEIDSLIEKLELQEKRDYPSKGLSGGQKRRLCVGIALSGAARVVLLDEPTSGMDPSSRR

HmelABCA2 DNEIDNLIDRLEMQEKRHYLARGLSGGQKRRLCVGIALSGGPRVVLLDEPTSGMDPSSRR

DpleABCA2 DDDIDKLIDSLEMQEKKNYLAEGLSGGQKRRLCVGIALCGGARVVLLDEPTSGMDPSSRR

PxylABCA2 AEEIESLIEKLEMQEKRDYQSGGLSGGQKRRLCVGIALSGGARVVLLDEPTSGMDPASRR

Dm_CG1718 EQEVAKYLKMIELEDKANVASSKLSGGMKRKLSVCCALCGDTKVVLCDEPSSGMDPSARR

:: :. :::: * . : **** **:*.. *:.* . *** ***:**:**.:**

BmorABCA1 ALWDLLQREKRARTVLLTTHFMDEADVLADRVAVLAAGRLACLGSPYFLKRHYGLGYKLA

HarmABCA1 ALWDLLQKEKKGRTMILTTHFMDEADVLGDRIAIMSGGRLQCVGTPYFLKKHYGIGYKIT

HvirABCA1 ALWDLLQKEKKGRTMILTTHFMDEADVLGDRIAIMSGGRLQCVGTPYFLKKHYGIGYKIT

HmelABCA1 SLWDLLQKEKKGRTMILTTHFMDEADVLGDRIAIMSGGRLQCVGTPYFLKKHYGIGYKLT

DpleABCA1 SLWDLLQREKK-------------------------------------------------

PxylABCA1 ALWDLLQSEKKGRSMVLTTHFMDEADYLGDRIAIMSGGKLQCVGTPYFLKKHYGLGYKLT

BmorABCA2 ALWDLLQREKRGRSIILTTHFMDEADVLGDRVAIMANGRLQCVGTPYFLKRHYGVGYTLV

HarmABCA2 ALWELLQKEKKGRSMILTTHFMDEADILGDRVAIMANGRLQCVGSPYFLKRHYGVGYTLV

HvirABCA2 ALWELLQKEKKGRSMILTTHFMDEADILGDRVAIMANGRLQCVGSPYFLKRHYGVGYTLV

HmelABCA2 ALWDLLQKEKKGRSIILTTHFMDEADYLGDRVAIMSSGRLQCIGSPYFLKHHYGVGYTLV

DpleABCA2 ALWELLQREKKNRSMILTTHFMDEADFLGDRVAIMSSGRLQCVGSPYFLKQHYGVGYTLV

PxylABCA2 ALWHLLQQEKKDRAMILTTHFMDEADYLGDRIAIMSGGKLQCVGSPYFLKKHYGVGYTLV

Dm_CG1718 QLWDLLQQEKVGRTLLLTTHFMDEADVLGDRIAIMCDGELKCQGTSFFLKKQYGSGYRLI

**.*** **

BmorABCA1 LVKDAACQVDLVTEFFKTYVPNLKQNSNIGSELTYILPSESVSKFPEMLKKLEEKKESLC

HarmABCA1 IVKGDNCIPDDITKFFKVYVPDIKENTNIGSELTYILPSDNVSKFPEMLKEFEAKKEALG

HvirABCA1 IVKGENCIPDDITKFFKTYVPDIKENTNIGSELTYILPSDNVSKFPEMLKEFEARKESLG

HmelABCA1 IVKCDDCNVEDVTKFFKTYVPETKENTNIGSELTYILPNEHVNKFPEMLKKFEELKDDLK

DpleABCA1 ------------------------------------------------------------

PxylABCA1 IVKGDNCNVEDVNRFLRGHVADVKENTNIGSELTYILLKEDVSKFPNLLKAFEEQKAALN

BmorABCA2 VVKKEGFDYTACTDLINKYIPGIAIKEDRGPEINYSLTNIQSHVFEDMLNDFEKNVNNIK

HarmABCA2 IVKDTDFDFVKCSVLINSYIPGTIVKEDRGTEITYNLVNDYSHVFEEMLNDLERNIDNIK

HvirABCA2 VVKDTDFDFVKCSVLINSYIPGTIVKEDRGTEITYNLVNDYSHVFEEMLNDLERNIDNIK

HmelABCA2 IVKNRDFQADQCTALIRRYIPGTMVKEDHGKEVTYNLPNEYSHVFEDLLNELEGTINNIQ

DpleABCA2 IVKNKDFQLDLCTSLIGKYIPGTIVKQDRGKEVTYSLPNNYSHLFEEMLNDLEKNYENIN

PxylABCA2 IVKGEAFNKDACTELIKKYIPDVPVGGDKGKEVTYSLSNDHSHIFEDLLTDLENNMESIG

Dm_CG1718 CVKRDDCETNEVTALLNKYIPGLKPECDIGAELSYQLPDSASAKFEEMFGQLEEQSDELH

BmorABCA1 ISSYGLSVTSLEEVFMKAGIEDNNVEIKETKGDIEMIDMNGD----MLNKYFI-------

HarmABCA1 VSSYGLSVTSLEEVFMKAGAED--IESPSAKSNNH---GSSR----AYNECAIAPL--AN

HvirABCA1 VSSYGLSVTSLEEVFMKAGAED--VESPSAKSNNH---GSSR----AYNECAIAPL--AN

HmelABCA1 VSSYGLSVTSLEEVFMKAGAED--NGVTSSMKNNY---INSSVQYTTHNDCAIVPIDRLN

DpleABCA1 ------------------------------------------------------------

PxylABCA1 VTSYGLSVTTLEEVFMKVGAESSKTPAESSASGSR---NYSGVHFNNNGNGMVRPLTEVE

BmorABCA2 FKDYGLIATTLEDVFMSVGSDVATINSVSDDGA-ASLDLSGDDNLKNEF---------SS

HarmABCA2 FKNYGLVATTLEDVFMSVGADLSPINSESDDAITTTTDSTIDDILKQEID--------SS

HvirABCA2 FKNYGVGATIAADVFMSVGADLNPVNSESDDAATTTTDSTIDDTLKQEID--------SS

HmelABCA2 FKSYGLVATTLEDVFMSVGSDVE-LSSESEDTTATADVSQ--DASVDDL----------S

DpleABCA2 YKNYGLIATTLEDVFMSVGSDVE-VNSESDDTTITATASECTDNDQNDL----------A

PxylABCA2 FKDYGLSATTIEDVFMSVGSDVVHDGADSDAA--TATDALSEDTECPGCEGSDCPGCGGS

Dm_CG1718 LNGYGVGITSMEEVFMKVGAEKDNTGNIKDQHEIMNGGSGFRGEDDNESV--------QS

BmorABCA1 --SEDN----EPLHKTQGFHLLKNHIKAMFLKLMYNTLRNKALAAIQIIWPIINIILSMI

HarmABCA1 HNSADN----EPIQKVRGYRLLRNHIKAMFLKLAYNSMRNKLTALIQFVTPIINITISVV

HvirABCA1 HNSADN----EPIQKVRGYKLLRNHIKAMFLKLAYNSMRNKLTALIQFVTPIINITISVV

HmelABCA1 NDILDN----EPIEKVRGFKLLRNHIKAMFLKLAYNSKRNMLAAFIQIVTPIINITISVL

DpleABCA1 ------------------------------------------------------------

PxylABCA1 HGHNDM----ELLQKVRGFKLLRNHIKAMFLKMFYHTKRNKLTNIIQFLSPVINIILSVI

BmorABCA2 LEQLTK------EGKVSGASLVGKHVLAVWMKLFLVWFRSWWMVLLQLAVPIVMMNATLL

HarmABCA2 LEELDK-----DESNVTGLRLFGQQVLAVWMKQWLVLIRSPWVMALQFLAPVILINSTLG

HvirABCA2 LEELDK-----DESNVTGCRLFGQQVLAVWMKQWLVLIRSPWLMVLQFMAPVILINATLG

HmelABCA2 LETCKRYIMNQSDDNETGLRLLWLHVSGVWLKLFLVWSRSWGLLLLQILVPILQINLSFA

DpleABCA2 LDQLHR-----SDESETGARLLWLHVSGIWLKLFWVSTRSWGMLLLQILVPIININASLA

PxylABCA2 TEYLDG-----QQQTLSGAALVWQHARAMWLKLWLVWSRSWGLLLLQILIPLVIINATLG

Dm_CG1718 DGIFSE-----NRRLLQGLQLLSNQWKAMLLKKFLYTWRNKLLLLIQNIMPVFFVVVTIL

BmorABCA1 VSLSW-KFLNVLPPLELSLESGFKGTETLVSQGNDLRDGSTEANVMMAYKDYFKRSTYPG

HarmABCA1 IARSW-KFLSQLPPLTLSLESGFATTETLMSQ-ANVTDGSIEAKAMTAYKDYFKKSSYPG

HvirABCA1 IARSW-KFLSQLPPLTLSLESGFAATETLMSQ-ANVTDGSIEAKAMTAYKDYFKKSTYPG

HmelABCA1 IARSW-KFMSQLPPLELSLESGFRKTETLLSQVANLTENSLEQRAMTAYKDYFKMASNPT

DpleABCA1 ------------------------------------------------------------

PxylABCA1 IARSW-NFLSELPPLTLSLEN-FKKSETLLSE-NSLVAGSLEEEAYRAFKDFYKESHQPN

BmorABCA2 IVQFLISFVANIQIRHLSLEYGYSRTETLLSF-NGSSSSSIGALASNAYENIFKSSGIDT

HarmABCA2 VLRYVMSLSPTIRTRWLSLEEGYTESETLLSF-NGSVASSVGALAAQAYQSLFANSGVMD

HvirABCA2 VLRYVMSLTPTIRTRLLTLEEGYTETETLVSF-NGTVATSVGALAAQAYHSLFTSSGISD

HmelABCA2 ILQYLVAAQSTVTPRTLTLAEGYMNTETFLRF-NGTNSSSLGSLAREGYKTLYNTADIDT

DpleABCA2 ILEYLFANRATVIPRALSLSQGYLSTETLLGF-NGTESSSLGARAVRGYELLYNTSNVES

PxylABCA2 ILQYMVSLTDNVQERHLTLNQGFLETETYLNY-NNV--SNITSGAVEAYRDLFNDS---T

Dm_CG1718 IIKTQ-GTFQELKPITISLTQ-YPLAVTVLDR-SNV-QNGTGYEIANKYEDLAR-SYGSN

BmorABCA1 LKLLDVGTSNLKNVYLKLIAE--DQSRVRYEDLVGATFRNNSITAWFSNYGLHDSAISLS

HarmABCA1 MTFTDLGTSNLGKFYLKLSEA--DLSRVRNEIPIGATFGAHNITAWFSNYGYHDSAISLA

HvirABCA1 MALTDLGTSNLGKFYLKLSEA--DLARVRNEIPIGATFGAHNITAWFSNYGYHDSAISLA

HmelABCA1 MMLTDIGTMDLSKFYLKLSKA--DLPRVRYENLVGATFSSQRITAWFGNYGYHDSAISLA

DpleABCA1 ------------------------------------------------------------

PxylABCA1 MILNDLGAADLGQTYLKMMKS--DSDRMRMETFTGATFGPGTITAWFSNYGYHDSAVALA

BmorABCA2 MEITIVEDQSIDEYYLERASDPIEMGALRHSVLTGATFSDDVATAWFSNFAYHDVATSLA

HarmABCA2 MEINAIGSQPIEEYYLNRTSDPVVMGSLRHRLLIGSTFDDDSATAWFSNFGYHDVATSLA

HvirABCA2 MEINDIGSQPVDQYYLNRTDDPVMMGLLRHRLLIGSTFDDDSATAWFSNFGYHDVATSLA

HmelABCA2 MKLSLVDDQNIEDYYLERTSDSVVLAAYRNRLLIGATFDDESGIAWFSNFGYHDVATSLA

DpleABCA2 MRLTVLDKTGVDEYYLNQTEDPVVMAAIRNQFLLGATFADKAAVAWFSNFGYHDVATSLA

PxylABCA2 TRLTVVEDDTIPEYYINQSTDALTLSTMRTQVLIGASFDNATATAWFSNFGYHDVAISLA

Dm_CG1718 YGLELTGTQGFEDYILDLGKT--IQVRINSRYLVAATITESKITAWLNNQALHTAPLTVN

BmorABCA1 LVENAIIRSLSPNTT-LTFVNHPLPYSVEGMVQVMSTGTNTAFMFSFSLGFCIAVISSFL

HarmABCA1 HVNNAIMGALSPGST-LKVVNHPLPYSIEHLVKVMASGSSMGFQFAFNIGFCMAFVTAFL

HvirABCA1 HVNNAIMGALSPGST-LKVVNHPLPYSIEHLVKVMASGSSMGFQFAFNIGFCMAFVTAFL

HmelABCA1 LANNALLRAMSPGTT-LRVVNHPLPYSIENLVKVMASGSSMGFQFAFNIGFCMAFVTSFL

DpleABCA1 -------------------------------VRVMASGSSMGFQFAFNIGFCMAFVTSFL

PxylABCA1 AVDRALMRARLPNAT-LTVRNHPLPYSIENLVRVMATGSSMGFQFAFNIGFCMAFVTSFL

BmorABCA2 SVYSALLRAFNSTAE-INVYNHPIEATYKNQNDMQLLVTMISMQLSSGVASCIAIVSAVF

HarmABCA2 AIHSAILRSKNSDAV-LNVYNHPLEASYIDQSDVQTMIAMLSMQLSSGIGSSVSIVSAVF

HvirABCA2 AIHSAILRSKNSAAV-LNVYNHPLEASYMDETDKQTMIAMLSMQLSSGIGSSVAIVSAVF

HmelABCA2 TLHEAMLSAVNSSAI-MKVYNYPLEANYNDDGDLQLLVSLLSTQVAGTIGNCLAIVSAVF

DpleABCA2 NVHSAILKGINPSAI-LNVFNYPLQATYRDRSDLQMMMSLLSMQVASSVGNSLAILSAAF

PxylABCA2 TLHKALLKAVLPGAQ-LNVTNHPLETNYEDTNDLRLIVVLLSMQFSNGLGNSVSIVSAVF

Dm_CG1718 MVHNAIADKLFGSSVKIQVTNAPLPYTTSTLLSQLSTGNNLGTQLASNLCFCMCFVSSIY

. .: : .:..:::

BmorABCA1 VLFVIKERISGAKLLQRVSGVRPVVMWSTALIWDWIWLFLNHICIIVTIACFQEMGMSTP

HarmABCA1 VLFVIKERISGAKLLQRVSGVRPAVMWVTSLIWDWMWLFMVYLCIVFTLACFQESTLSTP

HvirABCA1 VLFVIKERISGAKLLQRVSGVRPAVMWVTALIWDWMWLFMVYLCIVFTLACFQESTLATP

HmelABCA1 VLFVVKERTSGAKLLQRVSGVRPAIMWTTALVWDWFWLLMVFFAIVLTLAFFQENTLATP

DpleABCA1 VLFAIKERVSGAKLLQRVSGVRPAIMWTTALIWDWFWLFIVFIAIIVTLGLFQENTLATP

PxylABCA1 VLFVIKERITGARLLQRISGVRAAVRWGAAFVWDWLWLLLVYLCVVATLAAFQETTLSTP

BmorABCA2 VMFYIKERACGAKLLQKAAGIQPAVLWGSAAVFDWLLFSIICITIVITCAAFEVQGLSTF

HarmABCA2 IMFYIKERMSGAKLLQNAAGVAPAVLWGGAAIFNWFWFLITCVSIVISCVAFDVIGLSTV

HvirABCA2 IMFYIKERMSGAKLLQNAAGVQPFVLWGSAAIFNWFWFLITCVSIVISCVAFDVIGLSTV

HmelABCA2 IMFYIKERVTRAKLLQRAAGMQPAVMWGAAAVFDWLWFCVISVTIIISCAAFQIIGLSTV

DpleABCA2 VMFYIKERVTRAKLQQSAAGVRPAVMWGAAAVFDWLWFVVLCLPIIISCAAFAVLGLSTA

PxylABCA2 IMFYIKERLSRAKLLQKASGLQPVVMWGSAAVFDWLYFIIISLTFIVSCVAFQVTGLSSA

Dm_CG1718 ILFLIKERESRAKLLQFVGGVKVWTFWLSQFICDFASYIVTALIVVITIVCFQETGLSTF

::* :*** *:* * .*: * : :: : . .: : * :::

BmorABCA1 AELGRILLVLMVFSLAIIPLHYLASFCFEEAATGFSKMVFVNIFCGSMLFLVTEVLRMPF

HarmABCA1 AELGRVLLVLMVFSIATIPLHYLASFYFEAAATGFSKMCFMNIFTGCMPFLITELLRLPE

HvirABCA1 SELGRVLLVLMVFSIATIPLHYLASFYFEAAATGFSKMCFMNIFTGCMPFLITELLRIPE

HmelABCA1 EELGRVLLVLIIFAFAMIPLHYLASFYFEAAATGFSKMCFINILSGCMPFLITEVLRLPQ

DpleABCA1 AELGRVMLVLIIFAFAMIPLHYLASFYFEASATGFSKMCFINIFSGCMPFLITEVLRLPE

PxylABCA1 EELGRILLVLMAFSVAIIPLHYLASFYFESAATGFAKMCFLNIFCGSMPFLITEVLRLPE

BmorABCA2 VELGRMYLCLIVYGAAMLPLNYLLSHLFEGPALGFVVIFFTNCLLGMMGPQIVEALSSPQ

HarmABCA2 HELGRMFLCVMVYGAAMLPLVYLLSLKFKGPAVGFVGFYFLNVLFGMMGAQVVEALSSPM

HvirABCA2 HELGRMYLCVMLYGAAMLPLVYLMSLAFKGPAVGFVGFYFLNVLFGMMGAQVVEALSSPM

HmelABCA2 AELGRLYLFIIVYGAAMLPLHYLFSLVFNGPALGFVIMFFVNVLFGLIGAQIVEALSLPT

DpleABCA2 KELGYLFLCLMVYGAAMLPLHYLFSLLFNGPAIGFVILFFVNVLFGLLGAQIVEALRSIA

PxylABCA2 VELSRMFLLLVLYGAAVTPMLYLLSYLFSGPAFGFVVLVFTNSLLGLMGAQIVEALYSLD

Dm_CG1718 GELGRYYLLLLLFGFAVLPFIYIMSLFFREPATGFARVSIVNIFCGMALFIVVVVMSSEL

**. * :: :. * *: *: * * .* ** . : * : * :. :

BmorABCA1 INAAAYAEILEYPFSLLPIYCVSKSVREMVTSSIKIKACDSLCN-----QL-NYKNCTRL

HarmABCA1 VGSPFYAHLFDWIFSPLPIYCISRSFRDMSVSSFSMLACDGLCD-----QL-NIDNCTRH

HvirABCA1 VGSPFYAHLFDWIFSPLPIYCISRSFRDMSVSSFSMLACDGLCD-----QL-NIDNCTRH

HmelABCA1 VGNPYYAHIFDWVFSPLPIYCISRSFRDMSVSSFRLLACDALCK-----QFENIDNCTRH

DpleABCA1 VGNPYYAHIFDWVFSPLPIYCISRSFRDMSVSAFSLLACDALCA-----QLPGV-NCTRF

PxylABCA1 VGSPFYANIFDWIFSPLPIYCLSRSFRDMSMSSFAILACDGLCA-----QFANIRNCTRD

BmorABCA2 LNTQNVADILDTIFQFFPLYSLVTCVRELNQIGLVEYSCMRGCE-YISVLLPDLKECTMA

HarmABCA2 LDTEQAAHILDYLLQFYPLYSLVTSIRFLNQVGLREYTCLQACE-YLQAVYPNL-ECNMA

HvirABCA2 LDTEQVAHILDNILQFYPLYSLVTSIRFLNQVGLREYSCLQGCE-YLQAVYPNL-ECSMQ

HmelABCA2 LNTGDAARIMDYILPFFPLYSLVTATRKMNQVGLRVHSCLEYCG-YVTSIFPNITECTID

DpleABCA2 TSTKLAATALDYVLQFFPLYSLVTATRLMNQLGLKTFTCLEACANLLETVIGNTTSCNME

PxylABCA2 VNEQ-IPNIMDGILQFFPLYSLVTSVKKVHEISLKEWSCIKGCDFFISAGILTPDQCTPE

Dm_CG1718 FDTKDTADILGWIFRIFPHFSLAMSLNKVYTNTATRNACAKAGA------LPPILLCELV

. . : : * :.: . . : :* *

BmorABCA1 TICNELDISMCCIEDNPFLGWKEPGIARYLFTMIVVATVSFAILLAKEYELWNKTMML--

HarmABCA1 TICRQLNLSVCCIDDNPYMQWDEPGIGRYLFMMSMVGIISFTLLLIKEYELLNKVFYS--

HvirABCA1 TICRQLNLSVCCIDDNPYMQWDEPGIGRYLFMMSMVGIISFTLLLIKEYELLNKVFYS--

HmelABCA1 TVCDTLNVTVCCLEDDPFLKWSEPGIGRYLFTMSLVGIIGFTTLLVKEYELLNKVFYT--

DpleABCA1 TVCTKLNVSVCCMEDDPFLRWSEPGIGRYLFTMTLVGLISFTILLIKEYEILNKVFYS--

PxylABCA1 TICRSLNITVCCIKDDPYLDWHEPGIGRYLFTMTMVGIAAFALLMLKEYQFIEKICYK--

BmorABCA2 VMCEHY-LESCCVRESPYFEWEKPGVLRYLVVMLATCGVLWLILMTIEYRLFQKVFMF--

HarmABCA2 SMCEFH--SDCCVRENPYFDWEEPGVLRYLLSMCFSCLMFWLLLMTIEYRVVQKVFTF--

HvirABCA2 SMCEFH--TNCCVRENPYFDWEEPGILRYLLSMCFSSVIFWLVLMTIEYRLVQKVFTF--

HmelABCA2 ALCENF-SKNCCIRENPFFDWEDPGSLRYIVCSLASCILFWMLVMIIEYNWLQRLFTR--

DpleABCA2 FLCTQF-PDTCCVSES-LFEWEDPGILRYIICMIVTCVLMWTLLMVLEHNLIQRLLTK--

PxylABCA2 FLCSFN-QTTCCFEQDAYFNYEEPGVLRYILSTLLSCIIFWAMLMFIEYRIYSKIFNR--

Dm_CG1718 PQCCNL---------KPYFAWEEPGVLPETVYMAVTGVVFFLIIIVLEFRLINELMFKIR

* . : : .** . : :: *.. ..

BmorABCA1 ---SGTKPKSNESKKVEVNAEVEDDDVVEEKQRVLAMTSSEVTAHSLVCRELSKRYRRLV

HarmABCA1 ---SSNQP--------TPPILEEDSDVSTERSAVQEMTRAQISQQSLVCRDLTKFYKQFL

HvirABCA1 ---SSNQP--------TPPILEEDSDVSTERSAVQDMTRAQISQQSLVCRDLTKFYKQFL

HmelABCA1 ---NARTP-----VTLAAEVQDEDEDVRSERAAVQALPRAALARHSLVCRDLTKYYNDFL

DpleABCA1 ---ESKH-------GLPALVADEDSDVANERQTVRAFTRNELTQHSLVCRDLTKYYKDFL

PxylABCA1 ---PKPR-------AALPLDPDEDSDVAAERQIIENLKKNDAEKYSLVCKDLTKYYKDFL

BmorABCA2 ---RKIPP--------PIPENTLDKDVADEAEHVRQISKPDLARHGLVAKNLSKYYGSNL

HarmABCA2 ---KKTPP--------PIDESTLDEDVMTEARRARVVPPTRRREHALLAHDLSKYYGKHL

HvirABCA2 ---KKTPP--------PIDESTLDEDVKIEGQRARQVPPTRRAEHSLLAHDLSKYYGKHL

HmelABCA2 ---GKKAP--------PLNESSMDPDVLDEAKHANHVNET-APEIALVSRGLTKYYKDHL

DpleABCA2 ----RRSP--------PPSSDPVDEDVLEEAQHARRADLA-N---GLVARGLTKYYGNHL

PxylABCA2 ---NKVPP--------PIDPNSQDDDVVEEAKHVDFIGEAGTKQHNLVAKNLSKYYGKNL

Dm_CG1718 QLISKPPP--------PPTEGQLDDDVANERERILQMSSNELATKNLVLDRVTKYYGQFM

* ** * *: ::* * :

BmorABCA1 AVDRLTFAVRGGECFGLLGVNDAGYTSSVRMLTGDARVSDGDALVHGHSVRAHVQDVHRL

HarmABCA1 AVNRLTFAVHKGECFGLLGINGAGKTSTFRMLTGDAHISGGDAFVHGMSLKTHLQDVYRH

HvirABCA1 AVNRLTFAVHKGECFGLLGINGAGKTSTFRMLTGDAHISAGDAFVHGLSIKTHLQDVYRH

HmelABCA1 AVNRLTFAVRKGECFGLLGINGAGKTSTFRMVTGDARVSAGDAYVHGHSVRTHVRDVHRH

DpleABCA1 AVNRLSFAVHKGECFGLLGINGAGKTSTFRMLTGDSRLSCGDAYVHGLSLKTRIQDVHRH

PxylABCA1 AVNRLTFGVRKAECFGLLGINGAGKTSTFRMLTGDTDISAGDALVPGISIHGHMHQVHRV

BmorABCA2 AVDQVSFTVSDSECFGLLGVNGAGKTTTFKMLMGDENISSGEAFVSGYSVQQRLTKVHKN

HarmABCA2 AVDQVSFSVNDGECFGLLGVNGAGKTTTFKMLMGDESISSGEAYVSGHSVQRNLDRVHEN

HvirABCA2 AVDQVSFSVSDGECFGLLGVNGAGKTTTFKMLMGDESISSGEAFVSGHSVQRNLDKVHEN

HmelABCA2 AVDQISFTVSEAECFGLLGVNGAGKTTTFKMLMGDETISSGDAFVRGYSVKSDLTKVHAN

DpleABCA2 AVDNISFTVNEAEIFGLLGVNGAGKTTTFKMLMGDESISSGDAFISGYSVRNNITEVHQN

PxylABCA2 AVNQVSFSVGEMECFGLLGVNGAGKTTTFKMLMGDETVSSGDAFVRGHSVTKNIKKVHEN

Dm_CG1718 AVNQVSLCVQEVECFGLLGVNGAGKTTTFKMMTGDERISSGAAYVQGLSLESNMNSIYKM

**:.::: * * *****:*.** *::.:*: ** :* * * : * *: : ::

BmorABCA1 IGYCPQFDALFDNLTAREILKIFCLLRGIPTSIGETHAIHLAKQLGFIKHYDKKV-RECS

HarmABCA1 IGYCPQFDALFENLTGRETLRIFCLLRGIPVDVGNARALHLATTLGFLRHYDKKV-YECS

HvirABCA1 IGYCPQFDALFENLTGRETLRIFCLLRGIPAAVGNARALHLATTLGFLRHYDKKV-YECS

HmelABCA1 IGYCPQFDALLENLTARETLKIFCLLRGIPVKVGSARAIQLAEMLGFMRHYDKKV-QECS

DpleABCA1 IGYCPQFDALLENLTARETLKIFCLLRGIPVKVGSARAIQLAEMLGFFRHYDKKV-HECS

PxylABCA1 IGYCPQFDALLDNLTARETLRIFCLLRGIPARAGERRARSLAHALGFTRHYDKRI-HACS

BmorABCA2 IGYCPQFDALFGELTGRETLRLFSLLRGLRNFSGA-HADILAHSLGFLKHLDKRV-DQYS

HarmABCA2 IGYCPQFDALFGELTGRQTLHMFALMRGLRLRTAAPSAETLAHALGFFKHLDKRV-HQYS

HvirABCA2 IGYCPQFDALFGELTGRQTLHMFALMRGLRLRTAAPTAETLAHALGFFKHLDKMV-HQYS

HmelABCA2 IGYCPQFDAVFGELTGRETLHLFARFRGLKYANSSVRAEILANALGFTKHLDKRVIQQYS

DpleABCA2 IGYCPQFDAVFDELTGRETIHLFSRFRGLKYANSPVRAEIIANALGFTKHLDKRV-KQYS

PxylABCA2 IGYCPQFDALFEELTGRETLRFYALLRGLKPNTIVALTEYLANSLGFTKHLDKRV-YQYS

Dm_CG1718 IGYCPQFDALLDDLTGREVLRIFCMLRGVQESRIRQLSEDLAKSFGFMKHIDKQT-HAYS

*********:: :**.*: ::::. :**: : :* :** :* ** *

BmorABCA1 GGTKRKISTAVALLGDYPVIFLDEPTTGMDPASKRLVWRGISSAVGGGRSVVLTSHSMEE

HarmABCA1 GGTKRKISTAVSLLGDSSLVFLDEPTTGMDPASKRLVWSCISEAVMAGRSIVLTSHSMEE

HvirABCA1 GGTKRKISTAVSLLGDSSLVFLDEPTTGMDPASKRLVWSCISEAVVAGRSVVLTSHSMEE

HmelABCA1 GGTKRKISTALALLGDSPLVFLDEPTTGMDPASKRRVWACVQGAVRGGRSAVLTSHSMEE

DpleABCA1 GGTKRKISTALALLGDSPLVFLDEPTTGMDPASKRLVWRCVSEAAAGGRSVVLTSHSMEE

PxylABCA1 GGTKRKISTAVALLGDAPLVLLDEPTTGMDPASKRLVWECISAMVRGGRGCVLTSHSMEE

BmorABCA2 GGTKRKLNTAIAFLGTTRLIFVDEPTTGVDPAAKRHVWRAMRGVQRTGRGVVLTSHSMEE

HarmABCA2 GGTKRKLNTAIAFMGRTRLVFVDEPTTGVDPAAKRHVWRATRGVQRAGRGVVLTSHSMEE

HvirABCA2 GGTKRKLNTAIAFMGRTRLVFVDEPTTGVDPAAKRHVWRATRGVQRAGRGVVLTSHSMEE

HmelABCA2 GGNRRKLSTAVAMLGKTRLIFVDEPTTGVDPAAKRQVWRAIRAARRAGRAFVLTSHSMEE

DpleABCA2 GGNKRKLSTGVALLGRTGLVFVDEPTTGVDPAAKRTVWRALRDAKKAGRAFVLTSHSMEE

PxylABCA2 GGNKRKLSTAVALMGYGRCIFLDEPTTGVDPAAKRQVWRAIRKATRSGRSVVLTSHSMEE

Dm_CG1718 GGNKRKLSTAIAVIGSPSVIYLDEPTTGMDPAARRQLWNMVCRIRDSGKSIVLTSHSMEE

**.:**:.*.::.:* : :******:***::* :* *:. *********

BmorABCA1 CEALCSKLTVMVNGRLCCLGSLQHLKSKFSQGYTIIVKC----K----------------

HarmABCA1 CEALCSRLTVMVNGRLYCLGPLQHLKNKFSQGYTLIVKS----K----------------

HvirABCA1 CEALCSRLTVMVNGRLYCLGPLQHLKNKFSQGYTLIVKS----K----------------

HmelABCA1 CEALCSRLTIMVNGRLYCLGPLQHLKNKFSQGYTLIVKC----K----------------

DpleABCA1 CEALCSRLTVMVNGQLYCLGPLQHLKNKFSQGYTLIVKC----S----------------

PxylABCA1 CEALCSTLTVMVRGRLHCLGPLQHLKNKFSQGYTLVVKS----K----------------

BmorABCA2 CEALCSRLTIMVNGRFQCLGSPQHLKNKFSQGFTLIIKL----K----------------

HarmABCA2 CEALCSRLTIMVNGRFQCLGTPQHLKNKFSQGFTLIIKM----K----------------

HvirABCA2 CEALCSRLTIMVNGRFQCLGTPQHLKNKFSQGFTLIIKM----K----------------

HmelABCA2 CEALCSRLTIMVNGRFQCIGSPQHLKSKYSEGFTLTIKM----N----------------

DpleABCA2 CEALCDRLTIMVNGRFRCLGSPQHLKNKFSEGFTLTIKVLGRTN----------------

PxylABCA2 CEALCSRLTVMVNGQFMCLGTPQHLKNKFSEGFTLTIKL----N----------------

Dm_CG1718 CEALCTRLAIMVNGEFKCIGSTQHLKNKFSKGLILKIKV----RRNLEALRQARLSGGYA

***** *::**.*.: *:*. ****.*:*:* : :*

BmorABCA1 SGPNRDA--A---------VLDVHNYMTTNFVGANLIETYLGMSTYHVSSAGLPWWRVFS

HarmABCA1 SGPDRDA--D---------VARIDSYITEKFRESKLIESYLGISTYYINDTGLPWWSVFD

HvirABCA1 SGPDRDA--D---------VAKIDSYITEKFRESKLIESYLGISTYYINDTGLPWWCIFD

HmelABCA1 AGPDRDS--I---------VDKINQYVLENFRDAKLIETYLGISTYYLPDQGLSWWNVFH

DpleABCA1 SGADRDA--T---------VAKINQYVTDNFRDAKLIETYLGISTYYLNDQDLPWWRVFH

PxylABCA1 SGPNRDE--T---------SAEVQNYIMTEFPGAKFIEKYLGISTYYVAST-TSWWKVFS

BmorABCA2 SNEETNP--SSSRNQE----EAIKSYVTANFVNPKMMEEHQGLITYYLPDQTVPWSKMFG

HarmABCA2 TDDSEGD--TQSVNSTTSVVDSVKLYVSENFESPKIMEEYHGLLTYYLPDRSMAWSRMFG

HvirABCA2 IDDRESD--TQSLNSTCSVVDSVKQYVCDNFEAPKIMEEYHGLLTYYLPDRSMAWSRMFG

HmelABCA2 ADYAART--SISAESK---VQAVKDFVTTHYNDAKLMEEYQGIITYYLPDRSMAWSRMFG

DpleABCA2 ED-SPRT--SIKSESS---TQAVKQYVSDNFNNAKLMEEYQGLLTYYLPDRSVPWSKMFG

PxylABCA2 VADDLTN--VQ--------TVAVKDYVNLNFTEPKLMEEYQGLLTYYLPDRSMRWSRMFG

Dm_CG1718 RNPDEQTVPAQMSQRD---IDAVKEFVETEYPNSILQEEYQGILTFYIPLTGVKWSRIFG

:. :: .: . : * : *: *::: * :*

BmorABCA1 ALELARDSLPLDDYSVAQTTLEQVFLAFTKLQRPIN----

HarmABCA1 VMEEARRQFPIEDYSVSQTTLEQVFLQFTRLQGNETE-A-

HvirABCA1 VMEEARNQFPIEDYSVSQTTLEQVFLQFTRLQGNETD-A-

HmelABCA1 LMEEARKLYPIEDYSVSQTTLEQVFLRFTRIQGQPE----

DpleABCA1 LMEEARSQFPIEDYSVSQTTLEQVFLRFTRNQGRGD----

PxylABCA1 LMEAATQRFAIEDYSVSQTTLEQVFLRFTRLYGHDDRTS-

BmorABCA2 IMERAKQSLDVEDYSISQTTLEQIFLQFTKYQRE---GL-

HarmABCA2 IMERAKQILQIEDYSISQTTLEQIFLQFTKYQREEGTTL-

HvirABCA2 IMELAKRNLEIEDYSISQTTLEQIFLQFTKYQREEGETL-

HmelABCA2 IMEKSKRELDVEDYSILQTTLEQIFLQFTKYQQEGR----

DpleABCA2 IMEQAKRELDVEDYSIMQTTLEQIFLQFTKYQNEARET--

PxylABCA2 TMERAKRTLAVEDYSISQTTLEQIFLQFTKYQTQTTEL--

Dm_CG1718 LMESNRDQLNVEDYSVSQTTLEEIFLEFAKYQREDTRANQ

:* ::***: *****::** *::
