# Supplementary material for: Insect Resistance to Bacillus thuringiensis Toxin Cry2Ab Is Conferred by Mutations in an ABC Transporter Subfamily A Protein
Source: PLoS Genet. 2015 Nov 19;11(11):e1005534. doi: 10.1371/journal.pgen.1005534 (PMC4652872; doi:10.1371/journal.pgen.1005534)
Supplement: S2 Fig — Nucleotide variation (highlighted in orange) and deletions (‘-’ highlighted in green) are shown. The 8bp partial intron sequence (GGTTAAG) detected in the Ha2Ab-R01 allele is shown within the 72bp deletion (nt2892-2962) on exon 16. The 5bp deletion (ACAAG) of Ha2Ab-R02 is at nt3127-3131 on exon 18. The 5p insertion (GAATA) of Ha2Ab-R03 allele is between nt410-4104 (exon 24). Amino acid substitutions involving molecules with similar (hydrophobic) side chain are in red boxes, while substitutions between different category amino acids are in blue boxes. Amino acid symbols are: L (leucine), M (methionine), V (valine), I (isoleucine), Y (tyrosine), F (phenylalanine), A (alanine), P (proline), G (glycine), E (glutamic acid), K (lysine), and T (threonine). (PDF) [file pgen.1005534.s004.pdf]

S2 Fig

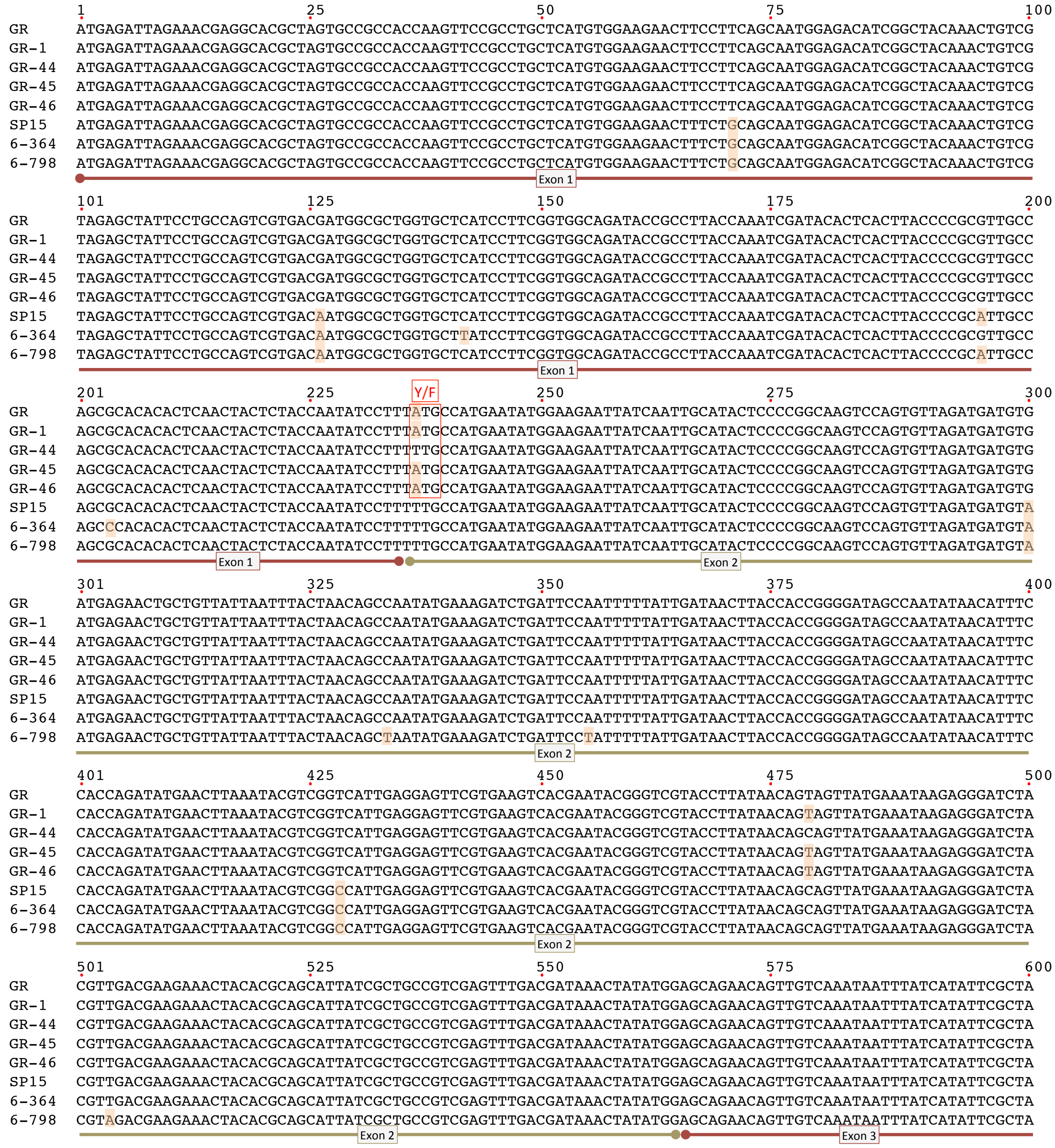
1
25
50
75
100
GR ATGAGATTAGAAACGAGGCACGCTAGTGCCGCCACCAAGTTCCGCCTGCTCATGTGGAAGAACTTCCTTCAGCAATGGAGACATCGGCTACAAACTGTCG
GR-1 ATGAGATTAGAAACGAGGCACGCTAGTGCCGCCACCAAGTTCCGCCTGCTCATGTGGAAGAACTTCCTTCAGCAATGGAGACATCGGCTACAAACTGTCG
GR-44 ATGAGATTAGAAACGAGGCACGCTAGTGCCGCCACCAAGTTCCGCCTGCTCATGTGGAAGAACTTCCTTCAGCAATGGAGACATCGGCTACAAACTGTCG
GR-45 ATGAGATTAGAAACGAGGCACGCTAGTGCCGCCACCAAGTTCCGCCTGCTCATGTGGAAGAACTTCCTTCAGCAATGGAGACATCGGCTACAAACTGTCG
GR-46 ATGAGATTAGAAACGAGGCACGCTAGTGCCGCCACCAAGTTCCGCCTGCTCATGTGGAAGAACTTCCTTCAGCAATGGAGACATCGGCTACAAACTGTCG
SP15 ATGAGATTAGAAACGAGGCACGCTAGTGCCGCCACCAAGTTCCGCCTGCTCATGTGGAAGAACTTTCTGCAGCAATGGAGACATCGGCTACAAACTGTCG
6-364 ATGAGATTAGAAACGAGGCACGCTAGTGCCGCCACCAAGTTCCGCCTGCTCATGTGGAAGAACTTTCTGCAGCAATGGAGACATCGGCTACAAACTGTCG
6-798 ATGAGATTAGAAACGAGGCACGCTAGTGCCGCCACCAAGTTCCGCCTGCTCATGTGGAAGAACTTTCTGCAGCAATGGAGACATCGGCTACAAACTGTCG
Exon 1
101
125
150
175
200
GR TAGAGCTATTCCTGCCAGTCGTGACGATGGCGCTGGTGCTCATCCTTCGGTGGCAGATACCGCCTTACCAAATCGATACACTCACTTACCCCGCGTTGCC
GR-1 TAGAGCTATTCCTGCCAGTCGTGACGATGGCGCTGGTGCTCATCCTTCGGTGGCAGATACCGCCTTACCAAATCGATACACTCACTTACCCCGCGTTGCC
GR-44 TAGAGCTATTCCTGCCAGTCGTGACGATGGCGCTGGTGCTCATCCTTCGGTGGCAGATACCGCCTTACCAAATCGATACACTCACTTACCCCGCGTTGCC
GR-45 TAGAGCTATTCCTGCCAGTCGTGACGATGGCGCTGGTGCTCATCCTTCGGTGGCAGATACCGCCTTACCAAATCGATACACTCACTTACCCCGCGTTGCC
GR-46 TAGAGCTATTCCTGCCAGTCGTGACGATGGCGCTGGTGCTCATCCTTCGGTGGCAGATACCGCCTTACCAAATCGATACACTCACTTACCCCGCGTTGCC
SP15 TAGAGCTATTCCTGCCAGTCGTGACAATGGCGCTGGTGCTCATCCTTCGGTGGCAGATACCGCCTTACCAAATCGATACACTCACTTACCCCGCATTGCC
6-364 TAGAGCTATTCCTGCCAGTCGTGACAATGGCGCTGGTGCTTATCCTTCGGTGGCAGATACCGCCTTACCAAATCGATACACTCACTTACCCCGCGTTGCC
6-798 TAGAGCTATTCCTGCCAGTCGTGACAATGGCGCTGGTGCTCATCCTTCGGTGGCAGATACCGCCTTACCAAATCGATACACTCACTTACCCCGCATTGCC
Exon 1
201
225
Y/F
250
275
300
GR AGCGCACACACTCAACTACTCTACCAATATCCTTTATGCCATGAATATGGAAGAATTATCAATTGCATACTCCCCGGCAAGTCCAGTGTTAGATGATGTG
GR-1 AGCGCACACACTCAACTACTCTACCAATATCCTTTATGCCATGAATATGGAAGAATTATCAATTGCATACTCCCCGGCAAGTCCAGTGTTAGATGATGTG
GR-44 AGCGCACACACTCAACTACTCTACCAATATCCTTTTTGCCATGAATATGGAAGAATTATCAATTGCATACTCCCCGGCAAGTCCAGTGTTAGATGATGTG
GR-45 AGCGCACACACTCAACTACTCTACCAATATCCTTTATGCCATGAATATGGAAGAATTATCAATTGCATACTCCCCGGCAAGTCCAGTGTTAGATGATGTG
GR-46 AGCGCACACACTCAACTACTCTACCAATATCCTTTATGCCATGAATATGGAAGAATTATCAATTGCATACTCCCCGGCAAGTCCAGTGTTAGATGATGTG
SP15 AGCGCACACACTCAACTACTCTACCAATATCCTTTTTGCCATGAATATGGAAGAATTATCAATTGCATACTCCCCGGCAAGTCCAGTGTTAGATGATGTA
6-364 AGCCCACACACTCAACTACTCTACCAATATCCTTTTTGCCATGAATATGGAAGAATTATCAATTGCATACTCCCCGGCAAGTCCAGTGTTAGATGATGTA
6-798 AGCGCACACACTCAACTACTCTACCAATATCCTTTTTGCCATGAATATGGAAGAATTATCAATTGCATACTCCCCGGCAAGTCCAGTGTTAGATGATGTA
Exon 1
Exon 2
301
325
350
375
400
GR ATGAGAACTGCTGTTATTAATTTACTAACAGCCAATATGAAAGATCTGATTCCAATTTTTATTGATAACTTACCACCGGGGATAGCCAATATAACATTTC
GR-1 ATGAGAACTGCTGTTATTAATTTACTAACAGCCAATATGAAAGATCTGATTCCAATTTTTATTGATAACTTACCACCGGGGATAGCCAATATAACATTTC
GR-44 ATGAGAACTGCTGTTATTAATTTACTAACAGCCAATATGAAAGATCTGATTCCAATTTTTATTGATAACTTACCACCGGGGATAGCCAATATAACATTTC
GR-45 ATGAGAACTGCTGTTATTAATTTACTAACAGCCAATATGAAAGATCTGATTCCAATTTTTATTGATAACTTACCACCGGGGATAGCCAATATAACATTTC
GR-46 ATGAGAACTGCTGTTATTAATTTACTAACAGCCAATATGAAAGATCTGATTCCAATTTTTATTGATAACTTACCACCGGGGATAGCCAATATAACATTTC
SP15 ATGAGAACTGCTGTTATTAATTTACTAACAGCCAATATGAAAGATCTGATTCCAATTTTTATTGATAACTTACCACCGGGGATAGCCAATATAACATTTC
6-364 ATGAGAACTGCTGTTATTAATTTACTAACAGCCAATATGAAAGATCTGATTCCAATTTTTATTGATAACTTACCACCGGGGATAGCCAATATAACATTTC
6-798 ATGAGAACTGCTGTTATTAATTTACTAACAGCTAATATGAAAGATCTGATTCCTATTTTTATTGATAACTTACCACCGGGGATAGCCAATATAACATTTC
Exon 2
401
425
450
475
500
GR CACCAGATATGAACTTAAATACGTCGGTCATTGAGGAGTTCGTGAAGTCACGAATACGGGTCGTACCTTATAACAGTAGTTATGAAATAAGAGGGATCTA
GR-1 CACCAGATATGAACTTAAATACGTCGGTCATTGAGGAGTTCGTGAAGTCACGAATACGGGTCGTACCTTATAACAGTAGTTATGAAATAAGAGGGATCTA
GR-44 CACCAGATATGAACTTAAATACGTCGGTCATTGAGGAGTTCGTGAAGTCACGAATACGGGTCGTACCTTATAACAGCAGTTATGAAATAAGAGGGATCTA
GR-45 CACCAGATATGAACTTAAATACGTCGGTCATTGAGGAGTTCGTGAAGTCACGAATACGGGTCGTACCTTATAACAGTAGTTATGAAATAAGAGGGATCTA
GR-46 CACCAGATATGAACTTAAATACGTCGGTCATTGAGGAGTTCGTGAAGTCACGAATACGGGTCGTACCTTATAACAGTAGTTATGAAATAAGAGGGATCTA
SP15 CACCAGATATGAACTTAAATACGTCGGCCATTGAGGAGTTCGTGAAGTCACGAATACGGGTCGTACCTTATAACAGCAGTTATGAAATAAGAGGGATCTA
6-364 CACCAGATATGAACTTAAATACGTCGGCCATTGAGGAGTTCGTGAAGTCACGAATACGGGTCGTACCTTATAACAGCAGTTATGAAATAAGAGGGATCTA
6-798 CACCAGATATGAACTTAAATACGTCGGCCATTGAGGAGTTCGTGAAGTCACGAATACGGGTCGTACCTTATAACAGCAGTTATGAAATAAGAGGGATCTA
Exon 2
501
525
550
575
600
GR CGTTGACGAAGAAACTACACGCAGCATTATCGCTGCCGTCGAGTTTGACGATAAACTATATGGAGCAGAACAGTTGTCAAATAATTTATCATATTCGCTA
GR-1 CGTTGACGAAGAAACTACACGCAGCATTATCGCTGCCGTCGAGTTTGACGATAAACTATATGGAGCAGAACAGTTGTCAAATAATTTATCATATTCGCTA
GR-44 CGTTGACGAAGAAACTACACGCAGCATTATCGCTGCCGTCGAGTTTGACGATAAACTATATGGAGCAGAACAGTTGTCAAATAATTTATCATATTCGCTA
GR-45 CGTTGACGAAGAAACTACACGCAGCATTATCGCTGCCGTCGAGTTTGACGATAAACTATATGGAGCAGAACAGTTGTCAAATAATTTATCATATTCGCTA
GR-46 CGTTGACGAAGAAACTACACGCAGCATTATCGCTGCCGTCGAGTTTGACGATAAACTATATGGAGCAGAACAGTTGTCAAATAATTTATCATATTCGCTA
SP15 CGTTGACGAAGAAACTACACGCAGCATTATCGCTGCCGTCGAGTTTGACGATAAACTATATGGAGCAGAACAGTTGTCAAATAATTTATCATATTCGCTA
6-364 CGTTGACGAAGAAACTACACGCAGCATTATCGCTGCCGTCGAGTTTGACGATAAACTATATGGAGCAGAACAGTTGTCAAATAATTTATCATATTCGCTA
6-798 CGTAGACGAAGAAACTACACGCAGCATTATCGCTGCCGTCGAGTTTGACGATAAACTATATGGAGCAGAACAGTTGTCAAATAATTTATCATATTCGCTA
Exon 2
Exon 3

```
        601                     625                      650                      675                      700
GR      CGTTTTCCTGAGAGACCCCGTCTCAATTCGTTTTTCCAAACTGGAGGGCGCACCTGGAGATCTGACGGAGTATTCCCGGTTTTCGAAGTGCCTGGGCCTA
GR-1    CGTTTTCCTGAGAGACCCCGTCTCAATTCGTTTTTCCAAACTGGAGGGCGCACCTGGAGATCTGACGGAGTATTCCCGGTTTTCGAAGTGCCTGGGCCTA
GR-44   CGTTTTCCTGAGAGACCCCGTCTCAATTCGTTTTTCCAAACTGGAGGGCGCACCTGGAGATCTGACGGAGTATTCCCGGTTTTCGAAGTGCCTGGGCCTA
GR-45   CGTTTTCCTGAGAGACCCCGTCTCAATTCGTTTTTCCAAACTGGAGGGCGCACCTGGAGATCTGACGGAGTATTCCCGGTTTTCGAAGTGCCTGGGCCTA
GR-46   CGTTTTCCTGAGAGACCCCGTCTCAATTCGTTTTTCCAAACTGGAGGGCGCACCTGGAGATCTGACGGAGTATTCCCGGTTTTCGAAGTGCCTGGGCCTA
SP15    CGTTTTCCTGAGAGACCCCGTCTCAATTCGTTTTTCCAAACTGGAGGGCGCACCTGGAGATCTGACGGAGTATTCCCGGTTTTCGAAGAGCCTGGGCCTA
6-364   CGTTTTCCTGAGAGACCCCGTCTCAATTCGTTTTTCCAAACTGGAGGGCGCACCTGGAGATCTGACGGAGTATTCCCGGTTTTCGAAGAGCCTGGGCCTA
6-798   CGTTTTCCTGAGAGACCCCGTCTCAATTCGTTTTTCCAAACTGGAGGGCGCACCTGGAGATCTGACGGAGTATTCCCGGTTTTCGAAGAGCCTGGGCCTA
                                                     Exon 3

        701                     725                      750                      775                      800
GR      GATTTCCTCACTCGTGGGAAGGTGGTAATGACCCAGGTTACGTTAACGAAATGTTCGTGGCACTTCAACAAGTTATATCAACGGAGCTGGTATCAAGGGC
GR-1    GATTTCCTCACTCGTGGGAAGGTGGTAATGACCCAGGTTACGTTAACGAAATGTTCGTGGCACTTCAACAAGTTATATCAACGGAGCTGGTGTCAAGGGC
GR-44   GATTTCCTCACTCGTGGGAAGGTGGTAATGACCCAGGTTACGTTAACGAAATGTTCGTGGCACTTCAACAAGTTATATCAACGGAGCTGGTGTCAAGGGC
GR-45   GATTTCCTCACTCGTGGGAAGGTGGTAATGACCCAGGTTACGTTAACGAAATGTTCGTGGCACTTCAACAAGTTATATCAACGGAGCTGGTGTCAAGGGC
GR-46   GATTTCCTCACTCGTGGGAAGGTGGTAATGACCCAGGTTACGTTAACGAAATGTTCGTGGCACTTCAACAAGTTATATCAACGGAGCTGGTGTCAAGGGC
SP15    GATTTCCTCACTCGTGGGAAGGTGGTAATGACCCAGGTTACGTTAACGAAATGTTCGTGGCACTTCAACAAGTTATATCAACGGAGCTGGTATCAAGGGC
6-364   GATTTCCTCACTCGTGGGAAGGTGGTAATGACCCAGGTTACGTTAACGAAATGTTCGTGGCACTTCAACAAGTTATATCAACGGAGCTGGTATCAAGGGC
6-798   GATTTCCTCACTCGTGGGAAGGTGGTAATGACCCAGGTTACGTTAACGAAATGTTCGTGGCACTTCAACAAGTTATATCAACGGAGCTGGTATCAAGGGC
                       Exon 3                                             Exon 4

        801                     825                      850                      875                      900
GR      AACCGGGTTGGACTTGAAGTCATTTAGGGTGAACATTCAGAGGTACCCGCACCCGCCGTACCTTCACGACCAGTCAGTTGATCTGCTGCAGTTCATGTTC
GR-1    AACCGGGTTGGACTTGAAGTCATTTAGGGTGAACATTCAGAGGTACCCGCACCCGCCGTACCTTCACGACCAGTCAGTTGATCTGCTGCAGTTCATGTTC
GR-44   AACCGGGTTGGACTTGAAGTCATTTAGGGTGAACATTCAGAGGTACCCGCACCCGCCGTACCTTCACGACCAGTCAGTTGATCTGCTGCAGTTCATGTTC
GR-45   AACCGGGTTGGACTTGAAGTCATTTAGGGTGAACATTCAGAGGTACCCGCACCCGCCGTACCTTCACGACCAGTCAGTTGATCTGCTGCAGTTCATGTTC
GR-46   AACCGGGTTGGACTTGAAGTCATTTAGGGTGAACATTCAGAGGTACCCGCACCCGCCGTACCTTCACGACCAGTCAGTTGATCTGCTGCAGTTCATGTTC
SP15    AACCGGGTTGGACTTGAAGTCCTTTAGGGTGAACATACAGAGGTACCCGCACCCGCCGTACCTTCACGACCAGTCAGTCGATCTGCTGCAGTTCATGTTT
6-364   AACCGGGTTGGACTTGAAGTCCTTCAGGGTGAACATACAGAGGTACCCGCACCCGCCGTACCTTCACGACCAGTCAGTTGATCTGCTGCAGTTCATGTTC
6-798   AACCGGGTTGGACTTGAAGTCCTTTAGGGTGAACATACAGAGGTACCCGCACCCGCCGTACCTTCACGACCAGTCAGTTGATCTGCTGCAGTTCATGTTT
                                                     Exon 4

        901                     925                      950                      975                      1000
GR      CCCCTGTTCATCATGTTGAGCTTCAGCTACACTTCCGTCAACATTGCACGGGCGGTCACAGTTGAGAAGGAATTGCAATTAAAGGAAACTATGAAAATTA
GR-1    CCCCTGTTCATCATGTTGAGCTTCAGCTACACTTCCGTCAACATTGCACGGGCGGTCACAGTTGAGAAGGAATTGCAATTAAAGGAAACTATGAAAATTA
GR-44   CCCCTGTTCATCATGTTGAGCTTCAGCTACACTTCCGTCAACATTGCACGGGCGGTCACAGTTGAGAAGGAATTGCAATTAAAGGAAACTATGAAAATTA
GR-45   CCCCTGTTCATCATGTTGAGCTTCAGCTACACTTCCGTCAACATTGCACGGGCGGTCACAGTTGAGAAGGAATTGCAATTAAAGGAAACTATGAAAATTA
GR-46   CCCCTGTTCATCATGTTGAGCTTCAGCTACACTTCCGTCAACATTGCACGGGCGGTCACAGTTGAGAAGGAATTGCAATTAAAGGAAACTATGAAAATTA
SP15    CCTCTGTTCATCATGTTGAGCTTCAGCTACACTTCCGTCAACATTGCACGGGCGGTCACAGTTGAGAAGGAATTGCAATTAAAGGAAACTATGAAAATTA
6-364   CCCCTGTTCATCATGTTGAGCTTCAGCTACACTTCCGTCAACATTGCACGGGCGGTCACAGTTGAGAAGGAATTGCAATTAAAGGAAACTATGAAAATTA
6-798   CCTCTGTTCATCATGTTGAGCTTCAGCTACACTTCCGTCAACATTGCACGGGCGGTCACAGTTGAGAAGGAATTGCAATTAAAGGAAACTATGAAAATTA
                                           Exon 4                                                  Exon 5

        1001                    1025                     1050                     1075                     1100
GR      TGGGTCTCCCCACATGGTTGCACTGGACAGCATGGTTTGTTAAACAGTTTATCTACCTATCAATCACAGCTGTTCTGCTAGTTGTGTTGCTAAAGGTAAA
GR-1    TGGGTCTCCCCACATGGTTGCACTGGACAGCATGGTTTGTTAAACAGTTTATCTACCTATCAATCACAGCTGTTCTGCTAGTTGTGTTGCTAAAGGTAAA
GR-44   TGGGTCTCCCCACATGGTTGCACTGGACAGCATGGTTTGTTAAACAGTTTATCTACCTATCAATCACAGCTGTTCTGCTAGTTGTGTTGCTAAAGGTAAA
GR-45   TGGGTCTCCCCACATGGTTGCACTGGACAGCATGGTTTGTTAAACAGTTTATCTACCTATCAATCACAGCTGTTCTGCTAGTTGTGTTGCTAAAGGTAAA
GR-46   TGGGTCTCCCCACATGGTTGCACTGGACAGCATGGTTTGTTAAACAGTTTATCTACCTATCAATCACAGCTGTTCTGCTAGTTGTGTTGCTAAAGGTAAA
SP15    TGGGTCTCCCCACATGGTTGCACTGGACAGCATGGTTTGTTAAACAGTTTATCTACCTATCAATCACAGCTGTTCTGCTAGTTGTGTTGCTAAAGGTAAA
6-364   TGGGTCTCCCCACATGGTTGCACTGGACAGCATGGTTTGTTAAACAGTTTATCTACCTATCAATCACAGCTGTTCTGCTAGTTGTGTTGCTAAAGGTAAA
6-798   TGGGCCTCCCCACATGGTTGCACTGGACAGCATGGTTTGTTAAACAGTTTATCTACCTATCAATCACAGCTGTTCTGCTAGTTGTGTTGCTAAAGGTAAA
                                                Exon 5                                                Exon 6

        1101                    1125                     1150                     1175                     1200
GR      TTGGTTTACTAACGACGATGGCTTCAGCGAATATGCTGTATTTACTAATACACCTTGGACGGTTTTGCTATTCTTCTTGATACTGTATTTATCTTGTGCG
GR-1    TTGGTTTACTAACGACGATGGCTTCAGCGAATATGCTGTATTTACTAATACACCTTGGACGGTTTTGCTATTGTTCTTGATACTGTATTTATCTTGCGCG
GR-44   TTGGTTTACTAACGACGATGGCTTCAGCGAATATGCTGTATTTACTAATACACCTTGGACGGTTTTGCTATTGTTCTTGATACTGTATTTATCTTGCGCG
GR-45   TTGGTTTACTAACGACGATGGCTTCAGCGAATATGCTGTATTTACTAATACACCTTGGACGGTTTTGCTATTGTTCTTGATACTGTATTTATCTTGCGCG
GR-46   TTGGTTTACTAACGACGATGGCTTCAGCGAATATGCTGTATTTACTAATACACCTTGGACGGTTTTGCTATTGTTCTTGATACTGTATTTATCTTGCGCG
SP15    TTGGTTTACTAACGACGATGGCTTCAGCGAATATGCTGTATTTACTAATACACCTTGGACGGTTTTGCTATTCTTCTTGATACTGTATTTATCTTGTGCG
6-364   TTGGTTTACTAACGACGATGGCTTCAGCGAATATGCTGTATTTACTAATACACCTTGGACGGTTTTGCTATTCTTCTTGATACTGTATTTATCTTGTGCG
6-798   TTGGTTTACTAACGACGATGGCTTCAGCGAATATGCTGTATTTACTAATACACCTTGGACGGTTTTGCTATTGTTCTTGATACTGTATTTATCTTGCGCG
                                                     Exon 6                L/F
```

```
       1201                      1225                     1250                     1275                        1300
GR     ATATTTTTTTCTTTCATGGTAAGCAGTATATTTTCAAAAGGTAGTACGGCCGCGTTGTTTATGGCGGTGGCATGGTTCCTCACTTACATCCCTGCTTTCC
GR-1   ATATTTTTTTCTTTCATGGTAAGCAGTATATTTTCAAAAGGTAGTACGGCCGCGTTGTTTATGGCGGTGGCATGGTTCCTCACTTACATCCCTGCTTTCC
GR-44  ATATTTTTTTCTTTCATGGTAAGCAGTATATTTTCAAAAGGTAGTACGGCCGCGTTGTTTATGGCGGTGGCATGGTTCCTCACTTACATCCCTGCTTTCC
GR-45  ATATTTTTTTCTTTCATGGTAAGCAGTATATTTTCAAAAGGTAGTACGGCCGCGTTGTTTATGGCGGTGGCATGGTTCCTCACTTACATCCCTGCTTTCC
GR-46  ATATTTTTTTCTTTCATGGTAAGCAGTATATTTTCAAAAGGTAGTACGGCCGCGTTGTTTATGGCGGTGGCATGGTTCCTCACTTACATCCCTGCTTTCC
SP15   ATATTTTTTTCTTTCATGGTAAGCAGTATATTTTCAAAAGGTAGTACGGCCGCGTTGTTTATGGCGGTGGCATGGTTCCTCACTTACATCCCTGCTTTCC
6-364  ATATTTTTTTCTTTCATGGTAAGCAGTATATTTTCAAAAGGTAGTACGGCCGCGTTGTTTATGGCGGTGGCATGGTTCCTCACTTACATCCCTGCTTTCC
6-798  ATATTTTTTTCTTTCATGGTAAGCAGTATATTTTCAAAAGGTAGTACGGCCGCGTTGTTTATGGCGGTGGCATGGTTCCTCACTTACATCCCTGCTTTCC
                         Exon 6                                                  Exon 7

       1301                      1325                     1350                     1375                        1400
GR     TCCTGGCCATGGATATCAATATGTCGACTGCGGTGCAGGTCATCACGTGCTTCAGTATTAACTCTGCGATGTCCTATGGTTTCCAACTAATGCTCGCTAA
GR-1   TCCTGGCCATGGATATCAATATGTCGACTGCGGTGCAGGTCATCACGTGCTTCAGTATTAACTCTGCGATGTCCTATGGTTTCCAACTAATGCTCGCTAA
GR-44  TCCTGGCCATGGATATCAATATGTCGACTGCGGTGCAGGTCATCACGTGCTTCAGTATTAACTCTGCGATGTCCTATGGTTTCCAACTAATGCTCGCTAA
GR-45  TCCTGGCCATGGATATCAATATGTCGACTGCGGTGCAGGTCATCACGTGCTTCAGTATTAACTCTGCGATGTCCTATGGTTTCCAACTAATGCTCGCTAA
GR-46  TCCTGGCCATGGATATCAATATGTCGACTGCGGTGCAGGTCATCACGTGCTTCAGTATTAACTCTGCGATGTCCTATGGTTTCCAACTAATGCTCGCTAA
SP15   TCCTGGCCATGGATATCAATATGTCGACTGCGGTGCAGGTCATCACGTGCTTCAGTATTAACTCTGCGATGTCCTATGGTTTCCAACTAATGCTCGCTAA
6-364  TCCTGGCCATGGATATCAATATGTCGACTGCGGTGCAGGTCATCACGTGCTTCAGTATAAACTCTGCGATGTCCTATGGTTTCCAACTAATGCTCGCTAA
6-798  TCCTGGCCATGGATATCAATATGTCGACTGCGGTGCAGGTCATCACGTGCTTCAGTATTAACTCTGCGATGTCCTATGGTTTCCAACTAATGCTCGCTAA
                                                        Exon 7

       1401                      1425                     1450                     1475                        1500
GR     GGAAAGCACTGGAGGGCTGCAGTGGGGCGACTTCATGACGTCACCAGAGACGGACACCACGCGCTTGGTGTTCGGCCACGTGGTCATCATGCTGGTAGTG
GR-1   GGAAAGCACTGGAGGGCTGCAGTGGGGCGACTTCATGACGTCACCAGGGACGGACACCACGCGCTTGGTGTTCGGCCACGTGGTCATCATGCTGGTAGTG
GR-44  GGAAAGCACTGGAGGGCTGCAGTGGGGCGACTTCATGACGTCACCAGGGACGGACACCACGCGCTTGGTGTTCGGCCACGTGGTCATCATGCTGGTAGTG
GR-45  GGAAAGCACTGGAGGGCTGCAGTGGGGCGACTTCATGACGTCACCAGGGACGGACACCACGCGCTTGGTGTTCGGCCACGTGGTCATCATGCTGGTAGTG
GR-46  GGAAAGCACTGGAGGGCTGCAGTGGGGCGACTTCATGACGTCACCAGGGACGGACACCACGCGCTTGGTGTTCGGCCACGTGGTCATCATGCTGGTAGTG
SP15   GGAAAGCACTGGAGGGCTGCAGTGGGGCGACTTCATGACGTCACCAGAGACGGACACCACGCGCTTGGTGTTCGGCCACGTGGTCATCATGCTGGTAGTG
6-364  GGAAAGCACTGGAGGGCTGCAGTGGGGCGACTTCATGACGTCACCAGAGACGGACACCACGCGCTTGGTGTTCGGCCACGTGGTCATCATGCTGGTAGTG
6-798  GGAAAGCACTGGAGGGCTGCAGTGGGGCGACTTCATGACGTCACCAGAGACGGACACCACGCGCTTGGTGTTCGGCCACGTGGTCATCATGCTGGTAGTG
            Exon 7                                      G/E          Exon 8

       1501                      1525                     1550                     1575                        1600
GR     GACTGCCTCATCTACATGCTGATCACCCTCTACCTTGAACAAGTGCTACCGGGACCCTTCGGGACTCCCAAACCTTGGTACTTCCCCTTCCAGTTGCAGT
GR-1   GACTGCCTCATCTACATGCTGATCACCCTCTACCTTGAACAAGTGCTACCAGGCCCCTTCGGGACTCCCAAACCTTGGTACTTCCCCTTCCAGTTGCAGT
GR-44  GACTGCCTCATCTACATGCTGATCACCCTCTACCTTGAACAAGTGCTACCAGGCCCCTTCGGGACTCCCAAACCTTGGTACTTCCCCTTCCAGTTGCAGT
GR-45  GACTGCCTCATCTACATGCTGATCACCCTCTACCTTGAACAAGTGCTACCAGGCCCCTTCGGGACTCCCAAACCTTGGTACTTCCCCTTCCAGTTGCAGT
GR-46  GACTGCCTCATCTACATGCTGATCACCCTCTACCTTGAACAAGTGCTACCAGGCCCCTTCGGGACTCCCAAACCTTGGTACTTCCCCTTCCAGTTGCAGT
SP15   GACTGCCTCATCTACATGCTGATCACCCTCTACCTTGAACAAGTGCTACCGGGACCCTTCGGGACTCCCAAACCTTGGTACTTCCCCTTCCAGTTGCAGT
6-364  GACTGCCTCATCTACATGCTGATCACCCTCTACCTTGAACAAGTGCTACCGGGACCCTTCGGGACTCCCAAACCTTGGTACTTCCCCTTCCAGTTGCAGT
6-798  GACTGCCTCATCTACATGCTGATCACCCTCTACCTTGAACAAGTGCTACCGGGACCCTTCGGGACTCCCAAACCTTGGTACTTCCCCTTCCAGTTGCAGT
                                                        Exon 8

       1601                      1625                     1650                     1675                        1700
GR     TCTGGTTCCCAAATTATAAATCGAAAGATGCTGGATTAATTTTCGAAAATGATAACAGTGAATTTGATGATATTATAAAAGAAAAGGATCCCACAGACCA
GR-1   TCTGGTTCCCAAATTATAAATCGAAAGATGCTGGATTATTTTTCGAAAATGATAACAGTGAATTTGATGATATTATAAAAGAAAAGGATCCCACAGACCA
GR-44  TCTGGTTCCCAAATTATAAATCGAAAGATGCTGGATTATTTTTCGAAAATGATAACAGTGAATTTGATGATATTATAAAAGAAAAGGATCCCACAGACCA
GR-45  TCTGGTTCCCAAATTATAAATCGAAAGATGCTGGATTATTTTTCGAAAATGATAACAGTGAATTTGATGATATTATAAAAGAAAAGGATCCCACAGACCA
GR-46  TCTGGTTCCCAAATTATAAATCGAAAGATGCTGGATTATTTTTCGAAAATGATAACAGTGAATTTGATGATATTATAAAAGAAAAGGATCCCACAGACCA
SP15   TCTGGTTCCCAAATTATAAATCGAAAGATGCTGGATTAATTTTCGAAAATGATAACAGTGAATTTGATGATATTATAAAAGAAAAGGATCCCACAGACCA
6-364  TCTGGTTCCCAAATTATAAATCGAAAGATGCTGGATTAATTTTCGAAAATGATAACAGTGAATTTGATGATTTTATAAAAGAAAAGGATCCCACAGACCA
6-798  TCTGGTTCCCAAATTATAAATCGAAAGATGCTGGATTAATTTTCGAAAATGATAACAGTGAATTTGATGATTTTATAAAAGAAAAGGATCCCACAGACCA
                  Exon 8                   I/F                      Exon 9

       1701                      1725                     1750                     1775                        1800
GR     CGAAGTTGGTGTTAGAATGCAAAATTTAACAAAAATCTTTGGGAATAACATAGCTGTTAACAATTTATCTTTGAATATCTATGACGATCAAATCACAGTT
GR-1   TGAAGTTGGTGTTAGAATGCAAAATTTAACAAAAATCTTTGGGAATAACATAGCTGTTAACAATTTATCTTTGAATATCTATGACGATCAAATCACAGTT
GR-44  TGAAGTTGGTGTTAGAATGCAAAATTTAACAAAAATCTTTGGGAATAACATAGCTGTTAACAATTTATCTTTGAATATCTATGACGATCAAATCACAGTT
GR-45  TGAAGTTGGTGTTAGAATGCAAAATTTAACAAAAATCTTTGGGAATAACATAGCTGTTAACAATTTATCTTTGAATATCTATGACGATCAAATCACAGTT
GR-46  TGAAGTTGGTGTTAGAATGCAAAATTTAACAAAAATCTTTGGGAATAACATAGCTGTTAACAATTTATCTTTGAATATCTATGACGATCAAATCACAGTT
SP15   CGAAGTTGGTGTTAGAATGCAAAATTTAACAAAAATCTTTGGGAATAACATAGCTGTTAACAATTTATCTTTGAATATCTATGACGATCAAATCACAGTT
6-364  CGAAGTTGGTGTTAGAATGCAAAATTTAACAAAAATCTTTGGGAATAACATAGCTGTTAACAATTTATCTTTGAATATCTATGACGATCAAATCACAGTT
6-798  CGAAGTTGGTGTTAGAATGCAAAATTTAACAAAAATCTTTGGGAATAACATAGCTGTTAACAATTTATCTTTGAATATCTATGACGATCAAATCACAGTT
                 Exon 9                                            Exon 10
```

```
       1801                    1825                     1850                     1875                     1900
GR     TTACTTGGTCATAATGGTGCTGGCAAATCTACTACAATATCTATGTTAACAGGAAATTTAAAGTTAACTCGCGGGACAGTGAACGTTGCCGGATATGATA
GR-1   TTACTTGGTCATAATGGTGCTGGCAAATCTACTACAATATCTATGTTAACAGGAAATTTAAAGTTAACTCGCGGGACAGTGAACGTTGCCGGATACGACA
GR-44  TTACTTGGTCATAATGGTGCTGGCAAATCTACTACAATATCTATGTTAACAGGAAATTTAAAGTTAACTCGCGGGACAGTGAACGTTGCCGGATACGACA
GR-45  TTACTTGGTCATAATGGTGCTGGCAAATCTACTACAATATCTATGTTAACAGGAAATTTAAAGTTAACTCGCGGGACAGTGAACGTTGCCGGATACGACA
GR-46  TTACTTGGTCATAATGGTGCTGGCAAATCTACTACAATATCTATGTTAACAGGAAATTTAAAGTTAACTCGCGGGACAGTGAACGTTGCCGGATACGACA
SP15   TTACTTGGTCATAATGGTGCTGGCAAATCTACTACAATATCTATGTTAACAGGAAATTTAAAGTTAACTCGCGGGACAGTGAACGTTGCCGGATATGATA
6-364  TTACTTGGTCATAATGGTGCTGGCAAATCTACTACAATATCTATGTTAACAGGAAATTTAAAGTTAACTCGCGGGACAGTGAACGTTGCCGGATATGATA
6-798  TTACTTGGTCATAATGGTGCTGGCAAATCTACTACAATATCTATGTTAACAGGAAATTTAAAGTTAACTCGCGGGACAGTGAACGTTGCCGGATATGATA
                                  Exon 10                                               Exon 11

       1901                    1925                     1950                     1975                     2000
GR     TGACCTCCCAAAGCTCAGCAGCCCGTTCGCACATCGGATTGTGTCCTCAGCACAATATACTGTTTAACGAACTCACGGTCAAAGAACATTTGGAATTCTT
GR-1   TGACTTCTCAAAGCTCCGCAGCCCGTTCCCACATTGGATTGTGTCCGCAGCACAATATACTGTTTAACGAACTCACGGTCAAAGAACATTTGGAATTCTT
GR-44  TGACTTCTCAAAGCTCCGCAGCCCGTTCCCACATTGGATTGTGTCCGCAGCACAATATACTGTTTAACGAACTCACGGTCAAAGAACATTTGGAATTCTT
GR-45  TGACTTCTCAAAGCTCCGCAGCCCGTTCCCACATTGGATTGTGTCCGCAGCACAATATACTGTTTAACGAACTCACGGTCAAAGAACATTTGGAATTCTT
GR-46  TGACTTCTCAAAGCTCCGCAGCCCGTTCCCACATTGGATTGTGTCCGCAGCACAATATACTGTTTAACGAACTCACGGTCAAAGAACATTTGGAATTCTT
SP15   TGACCTCCCAAAGCTCAGCAGCCCGTTCGCACATCGGATTGTGTCCTCAGCACAATATACTGTTTAACGAACTCACGGTCAAAGAACATTTGGAATTCTT
6-364  TGACCTCCCAAAGCTCAGCAGCCCGTTCGCACATCGGATTGTGTCCTCAGCACAATATACTGTTTAACGAACTCACGGTCAAAGAACATTTGGAATTCTT
6-798  TGACCTCCCAAAGCTCAGCAGCCCGTTCGCACATCGGATTGTGTCCTCAGCACAATATACTGTTTAACGAACTCACGGTCAAAGAACATTTGGAATTCTT
                                                     Exon 11

       2001                    2025                     2050                     2075                     2100
GR     TGCTAGACTAAAAGGATTCAAAGGCAAAGAACTGTATGACGAGATAGACTCACTTATTGAAAAATTGGAACTACAGGAGAAGCGTGACTATCCTTCAAAA
GR-1   TGCTAGACTAAAAGGATTCAAAGGCAAAGAACTGTATGACGAGATAGACTCACTTATTGAAAAATTGGAACTACAGGAGAAGCGTGACTATCCTTCAAAA
GR-44  TGCTAGACTAAAAGGATTCAAAGGCAAAGAACTGTATGACGAGATAGACTCACTTATTGAAAAATTGGAACTACAGGAGAAGCGTGACTATCCTTCAAAA
GR-45  TGCTAGACTAAAAGGATTCAAAGGCAAAGAACTGTATGACGAGATAGACTCACTTATTGAAAAATTGGAACTACAGGAGAAGCGTGACTATCCTTCAAAA
GR-46  TGCTAGACTAAAAGGATTCAAAGGCAAAGAACTGTATGACGAGATAGACTCACTTATTGAAAAATTGGAACTACAGGAGAAGCGTGACTATCCTTCAAAA
SP15   TGCTAGACTAAAAGGATTCAAAGGCAAAGAACTGTATGACGAGATAGACTCACTTATTGAAAAATTGGAACTACAGGAGAAGCGTGACTATCCTTCAAAA
6-364  TGCTAGACTAAAAGGATTCAAAGGCAAAGAACTGTATGACGAGATAGACTCACTTATTGAAAAATTGGAACTACAGGAGAAGCGTGACTATCCTTCAAAA
6-798  TGCTAGACTAAAAGGATTCAAAGGCAAAGAACTGTATGACGAGATAGACTCACTTATTGAAAAATTGGAACTACAGGAGAAGCGTGACTATCCTTCAAAA
                                         Exon 11                                                 Exon 12

       2101                    2125                     2150                     2175                     2200
GR     GGTTTGTCAGGAGGTCAGAAGCGTCGTCTTTGTGTTGGTATTGCTCTGAGTGGGGCGGCACGAGTGGTCTTACTCGACGAACCTACGTCTGGCATGGATC
GR-1   GGTTTGTCAGGAGGTCAGAAGCGTCGTCTTTGTGTTGGTATTGCTCTGAGTGGGGCGGCACGAGTGGTCTTACTCGACGAGCCAACGTCTGGCATGGATC
GR-44  GGTTTGTCAGGAGGTCAGAAGCGTCGTCTTTGTGTTGGTATTGCTCTGAGTGGGGCGGCACGAGTGGTCTTACTCGACGAGCCAACGTCTGGCATGGATC
GR-45  GGTTTGTCAGGAGGTCAGAAGCGTCGTCTTTGTGTTGGTATTGCTCTGAGTGGGGCGGCACGAGTGGTCTTACTCGACGAGCCAACGTCTGGCATGGATC
GR-46  GGTTTGTCAGGAGGTCAGAAGCGTCGTCTTTGTGTTGGTATTGCTCTGAGTGGGGCGGCACGAGTGGTCTTACTCGACGAGCCAACGTCTGGCATGGATC
SP15   GGTTTGTCAGGAGGTCAGAAGCGTCGTCTTTGTGTTGGTATTGCTCTGAGTGGGGCGGCACGAGTGGTCTTACTCGACGAACCTACGTCTGGCATGGATC
6-364  GGTTTGTCAGGAGGTCAGAAGCGTCGTCTTTGTGTTGGTATTGCTCTGAGTGGGGCGGCACGAGTGGTCTTACTCGACGAACCTACGTCTGGCATGGATC
6-798  GGTTTGTCAGGAGGTCAGAAGCGTCGTCTTTGTGTTGGTATTGCTCTGAGTGGGGCGGCACGAGTGGTCTTACTCGACGAACCTACGTCTGGCATGGATC
                                                     Exon 12

       2201                    2225                     2250                     2275                     2300
GR     CTTCATCTCGCCGAGCACTGTGGGAACTCTTACAGAAGGAAAAGAAAGGTCGCTCGATGATCCTGACGACTCATTTCATGGACGAAGCAGATATTCTCGG
GR-1   CTTCATCTCGTCGAGCACTGTGGGAACTCTTACAGAAGGAAAAGAAAGGTCGCTCGATGATCCTGACGACTCATTTCATGGACGAAGCAGATATTCTCGG
GR-44  CTTCATCTCGTCGAGCACTGTGGGAACTCTTACAGAAGGAAAAGAAAGGTCGCTCGATGATCCTGACGACTCATTTCATGGACGAAGCAGATATTCTCGG
GR-45  CTTCATCTCGTCGAGCACTGTGGGAACTCTTACAGAAGGAAAAGAAAGGTCGCTCGATGATCCTGACGACTCATTTCATGGACGAAGCAGATATTCTCGG
GR-46  CTTCATCTCGTCGAGCACTGTGGGAACTCTTACAGAAGGAAAAGAAAGGTCGCTCGATGATCCTGACGACTCATTTCATGGACGAAGCAGATATTCTCGG
SP15   CTTCATCTCGCCGAGCACTGTGGGAACTCTTACAGAAGGAAAAGAAAGGTCGCTCGATGATCCTGACGACTCATTTCATGGACGAAGCAGATATTCTCGG
6-364  CTTCATCTCGCCGAGCACTGTGGGAACTCTTACAGAAGGAAAAGAAAGGTCGCTCGATGATCCTGACGACTCATTTCATGGACGAAGCAGATATACTCGG
6-798  CTTCATCTCGCCGAGCACTGTGGGAACTCTTACAGAAGGAAAAGAAAGGTCGCTCGATGATCCTGACGACTCATTTCATGGACGAAGCAGATATACTCGG
                                 Exon 12                                                Exon 13

       2301                    2325                     2350                     2375                     2400
GR     CGACAGAGTGGCTATAATGGCAAACGGTAGACTGCAATGCGTGGGCTCTCCGTATTTCCTCAAACGTCATTATGGCGTCGGGTACACCCTTGTCATCGTT
GR-1   CGACAGAGTGGCTATAATGGCAAACGGTAGACTGCAATGCGTGGGCTCTCCGTATTTCCTCAAACGTCATTATGGCGTCGGGTACACCCTTGTCATCGTT
GR-44  CGACAGAGTGGCTATAATGGCAAACGGTAGACTGCAATGCGTGGGCTCTCCGTATTTCCTCAAACGTCATTATGGCGTCGGGTACACCCTTGTCATCGTT
GR-45  CGACAGAGTGGCTATAATGGCAAACGGTAGACTGCAATGCGTGGGCTCTCCGTATTTCCTCAAACGTCATTATGGCGTCGGGTACACCCTTGTCATCGTT
GR-46  CGACAGAGTGGCTATAATGGCAAACGGTAGACTGCAATGCGTGGGCTCTCCGTATTTCCTCAAACGTCATTATGGCGTCGGGTACACCCTTGTCATCGTT
SP15   CGACAGAGTGGCTATAATGGCAAACGGTAGACTGCAATGCGTGGGCTCTCCGTATTTCCTCAAACGTCATTATGGCGTCGGGTACACCCTTGTCATCGTT
6-364  CGACAGAGTGGCTATAATGGCAAACGGTAGACTGCAATGCGTGGGCTCTCCGTATTTCCTCAAACGTCATTATGGCGTCGGGTACACCCTCGTGATCGTT
6-798  CGACAGAGTGGCTATAATGGCAAACGGTAGACTGCAATGCGTGGGCTCTCCGTATTTCCTCAAACGTCATTATGGCGTCGGGTACACCCTCGTGATCGTT
                                                     Exon 13
```

```
      2401                    2425                     2450                     2475                     2500
GR    AAGGACACAGACTTCGACTTTGTGAAATGCTCCGTACTTATCAATAGCTACATTCCTGGTACTATTGTTAAAGAAGATCGAGGAACGGAAATCACTTATA
GR-1  AAGGACACAGACTTCGACTTTGTGAAATGCTCCGTACTTATCAATAGCTACATTCCTGGTACTATTGTTAAAGAAGATCGAGGAACGGAAATCACTTATA
GR-44 AAGGACACAGACTTCGACTTTGTGAAATGCTCCGTACTTATCAATAGCTACATTCCTGGTACTATTGTTAAAGAAGATCGAGGAACGGAAATCACTTATA
GR-45 AAGGACACAGACTTCGACTTTGTGAAATGCTCCGTACTTATCAATAGCTACATTCCTGGTACTATTGTTAAAGAAGATCGAGGAACGGAAATCACTTATA
GR-46 AAGGACACAGACTTCGACTTTGTGAAATGCTCCGTACTTATCAATAGCTACATTCCTGGTACTATTGTTAAAGAAGATCGAGGAACGGAAATCACTTATA
SP15  AAGGACACAGACTTCGACTTTGTGAAATGCTCCGTACTTATCAATAGCTACATTCCTGGTACTATTGTTAAAGAAGATCGAGGAACGGAAATCACTTATA
6-364 AAGGACACAGACTTCGACTTTGTGAAATGCTCCGTACTTATCAATAGCTATATTCCTGGTACTATTGTTAAAGAAGATCGAGGAACGGAAATCACTTATA
6-798 AAGGACACAGACTTCGACTTTGTGAAATGCTCCGTACTTATCAATAGCTATATTCCTGGTACTATTGTTAAAGAAGATCGAGGAACGGAAATCACTTATA
                                          Exon 13                                              Exon 14

      2501                    2525                     2550                     2575                     2600
GR    ATTTGGTAAATGATTACTCACACGTTTTTGAAGAAATGTTGAATGATTTGGAAAGAAATATTGATAACATCAAATTTAAGAACTATGGTTTAGTTGCTAC
GR-1  ATTTGGTAAATGATTACTCACACGTTTTTGAAGAAATGTTGAATGATTTGGAAAGAAATATTGATAATATCAAATTTAAAAACTATGGTTTAGTTGCTAC
GR-44 ATTTGGTAAATGATTACTCACACGTTTTTGAAGAAATGTTGAATGATTTGGAAAGAAATATTGATAATATCAAATTTAAAAACTATGGTTTAGTTGCTAC
GR-45 ATTTGGTAAATGATTACTCACACGTTTTTGAAGAAATGTTGAATGATTTGGAAAGAAATATTGATAATATCAAATTTAAAAACTATGGTTTAGTTGCTAC
GR-46 ATTTGGTAAATGATTACTCACACGTTTTTGAAGAAATGTTGAATGATTTGGAAAGAAATATTGATAATATCAAATTTAAAAACTATGGTTTAGTTGCTAC
SP15  ATTTGGTAAATGATTACTCACACGTTTTTGAAGAAATGTTGAATGATTTGGAAAGAAATATTGATAACATCAAATTTAAGAACTATGGTTTAGTTGCTAC
6-364 ATTTGGTAAATGATTACTCACACGTTTTTGAAGAAATGTTGAATGATTTGGAAAGAAATATTGATAACATCAAATTTAAAAACTATGGTTTAGTTGCTAC
6-798 ATTTGGTAAATGATTACTCACACGTTTTTGAAGAAATGTTGAATGATTTGGAAAGAAATATTGATAACATCAAATTTAAAAACTATGGTTTAGTTGCTAC
                                                   Exon 14

      2601                    2625                     2650                     2675                     2700
GR    TACATTAGAAGATGTCTTTATGTCCGTTGGTGCCGACTTAAGTCCAATTAATTCCGAATCTGACGATGCTATTACTACTACTACTGATTCAACTATCGAT
GR-1  TACATTAGAAGATGTCTTTATGTCCGTTGGTGCCGACTTAAGTCCAATTAATTCCGAATCTGACGATGCTATTACTACTACTACTGATTCAACTATCGAT
GR-44 TACATTAGAAGATGTCTTTATGTCCGTTGGTGCCGACTTAAGTCCAATTAATTCCGAATCTGACGATGCTATTACTACTACTACTGATTCAACTATCGAT
GR-45 TACATTAGAAGATGTCTTTATGTCCGTTGGTGCCGACTTAAGTCCAATTAATTCCGAATCTGACGATGCTATTACTACTACTACTGATTCAACTATCGAT
GR-46 TACATTAGAAGATGTCTTTATGTCCGTTGGTGCCGACTTAAGTCCAATTAATTCCGAATCTGACGATGCTATTACTACTACTACTGATTCAACTATCGAT
SP15  TACATTAGAAGATGTCTTTATGTCCGTTGGTGCAGACTTAAGTCCAATTAATTCCGAATCTGACGATGCTATTACTACTACTACTGATTCAACTATCGAT
6-364 TACATTAGAAGATGTCTTTATGTCCGTTGGTGCCGACTTAAGTCCAATTAATTCCGAATCTGACGATGCTATTACTACTACTACTGATTCAACTATCGAT
6-798 TACATTAGAAGATGTCTTTATGTCCGTTGGTGCCGACTTAAGTCCAATTAATTCCGAATCTGACGATGCTATTACTACTACTACTGATTCAACTATCGAT
                Exon 14                                                Exon 15

      2701                    2725                     2750                     2775                     2800
GR    GATATATTAAAACAAGAAATCGATTCATCTTTGGAAGAACTGGATAAGGACGAGAGTAACGTGACGGGCCTCCGCTTGTTCGGTCAGCAAGTGCTGGCTG
GR-1  GATATATTAAAACAAGAAATCGATTCATCTTTGGAAGAACTGGATAAGGACGAGAGTAACGTGACGGGCCTCCGCTTGTTCGGTCAGCAAGTGCTGGCTG
GR-44 GATATATTAAAACAAGAAATCGATTCATCTTTGGAAGAACTGGATAAGGACGAGAGTAACGTGACGGGCCTCCGCTTGTTCGGTCAGCAAGTGCTGGCTG
GR-45 GATATATTAAAACAAGAAATCGATTCATCTTTGGAAGAACTGGATAAGGACGAGAGTAACGTGACGGGCCTCCGCTTGTTCGGTCAGCAAGTGCTGGCTG
GR-46 GATATATTAAAACAAGAAATCGATTCATCTTTGGAAGAACTGGATAAGGACGAGAGTAACGTGACGGGCCTCCGCTTGTTCGGTCAGCAAGTGCTGGCTG
SP15  GATATATTAAAACAAGAAATCGATTCATCTTTGGAAGAACTGGATAAGGACGAGAGTAACGTGACGGGCCTCCGCTTGTTCGGTCAGCAAGTGCTGGCTG
6-364 GATATATTAAAACAAGAAATCGATTCATCTTTGGAAGAACTGGATAAGGACGAGAGTAACGTGACGGGCCTCCGCTTGTTCGGTCAGCAAGTGCTGGCTG
6-798 GATATATTAAAACAAGAAATCGATTCATCTTTGGAAGAACTGGATAAGGACGAGAGTAACGTGACGGGCCTCCGCTTGTTCGGTCAGCAAGTGCTGGCTG
                      Exon 15                                                      Exon 16

      2801                    2825                     2850                     2875                     2900
GR    TATGGATGAAGCAGTGGCTGGTGCTGATCCGCTCGCCATGGGTCATGGCACTGCAGTTCTTGGCGCCAGTGATACTCATCAACTCCACGCTAGGAGTTCT
GR-1  TATGGATGAAGCAGTGGCTGGTGCTGATCCGCTCGCCGTGGGTCATGGCACTGCAGTTCTTGGCGCCAGTGGTACTCATCAACTCTACGCTAGGAGTTCT
GR-44 TATGGATGAAGCAGTGGCTGGTGCTGATCCGCTCGCCATGGGTCATGGCACTGCAGTTCTTGGCGCCAGTGATACTCATCAACTCTACGCTAGGAGTTCT
GR-45 TATGGATGAAGCAGTGGCTGGTGCTGATCCGCTCGCCGTGGGTCATGGCACTGCAGTTCTTGGCGCCAGTGGTACTCATCAACTCTACGCTAGGAGTTCT
GR-46 TATGGATGAAGCAGTGGCTGGTGCTGATCCGCTCGCCGTGGGTCATGGCACTGCAGTTCTTGGCGCCAGTGGTACTCATCAACTCTACGCTAGGAGTTCT
SP15  TATGGATGAAGCAGTGGCTGGTGCTGATCCGCTCGCCATGGGTCATGGCACTGCAGTTCTTGGCGCCAGTGATACTCATCAACTCCACC-----------
6-364 TATGGATGAAGCAGTGGCTGGTGCTCATCCGCTCGCCATGGGTCATGGTACTGCAGTTCTTGGCGCCAGTGATACTCATCAACTCCACACTAGGAGTTCT
6-798 TATGGATGAAGCAGTGGCTGGTGCTCATCCGCTCGCCATGGGTCATGGTACTGCAGTTCTTGGCGCCAGTGATACTCATCAACTCCACACTAGGAGTTCT
                                                   Exon 16                V/I

      2901                    2925                     2950                     2975                     3000
GR    GCGTTACGTCATGTCTTTATCACCAACCATTAGAACTAGGTGGTTGTCGTTGGAAGAAGGGTATACGGAAAGCGAAACTCTGCTCAGCTTCAACGGCAGT
GR-1  GCGTTACGTCATGTCTTTATCACCAACCATTAGAACTAGGTGGTTGTCGTTGGAAGAAGGGTATACGGAAAGCGAAACTCTGCTCAGCTTCAACGGCAGT
GR-44 GCGTTACGTCATGTCTTTATCACCAACCATTAGAACTAGGTGGTTGTCGTTGGAAGAAGGGTATACGGAAAGCGAAACTCTGCTCAGCTTCAACGGCAGT
GR-45 GCGTTACGTCATGTCTTTATCACCAACCATTAGAACTAGGTGGTTGTCGTTGGAAGAAGGGTATACGGAAAGCGAAACTCTGCTCAGCTTCAACGGCAGT
GR-46 GCGTTACGTCATGTCTTTATCACCAACCATTAGAACTAGGTGGTTGTCGTTGGAAGAAGGGTATACGGAAAGCGAAACTCTGCTCAGCTTCAACGGCAGT
SP15  ---GGTTAAG---------------------------------------------------TATACGGAAAGCGAAACTCTGCTGAGCTTCAACGGCAGT
6-364 GCGTTACGTCATGTCTTTATCACCAACCATTAGAACTAGGTGGTTGTCGTTGGAAGAAGGGTATACGGAAAGCGAAACTCTGCTCAGCTTCAACGGCAGT
6-798 GCGTTACGTCATGTCTTTATCACCAACCATTAGAACTAGGTGGTTGTCGTTGGAAGAAGGGTATACGGAAAGCGAAACTCTGCTCAGCTTCAACGGCAGT
                                Exon 16                                           Exon 17
```

```
       3001                    3025                     3050                     3075                     3100
GR     GTAGCGTCATCAGTGGGCGCCCTAGCGGCGCAAGCATACCAAAGCCTGTTCGCCAATTCTGGTGTTATGGACATGGAAATCAACGCTATTGGAAGCCAGC
GR-1   GTAGCGTCATCAGTGGGCGCCCTAGCGGCGCAAGCATACCAAAGCTTGTTCGCCAATTCTGGTGTTATGGACATGGAAATCAACGCTATCGGAAGCCAAG
GR-44  GTAGCGTCATCAGTGGGCGCCCTAGCGGCGCAAGCATACCAAAGCTTGTTCGCCAATTCTGGTGTTATGGACATGGAAATCAACGCTATCGGAAGCCAAG
GR-45  GTAGCGTCATCAGTGGGCGCCCTAGCGGCGCAAGCATACCAAAGCTTGTTCGCCAATTCTGGTGTTATGGACATGGAAATCAACGCTATCGGAAGCCAAG
GR-46  GTAGCGTCATCAGTGGGCGCCCTAGCGGCGCAAGCATACCAAAGCTTGTTCGCCAATTCTGGTGTTATGGACATGGAAATCAACGCTATCGGAAGCCAAG
SP15   GTATCGTCATCAGTGGGTGCCCTAGCCGCGCAAGCATACCAAAGCCTGTTCGCCAATTCTGGAGTTATGGACATGGAAATCAACGCTATTGGAAGCCAGC
6-364  GTAGCGTCATCAGTGGGCGCCCTAGCGGCGCAAGCGTACCAAAGCCTGTTCGCCAATTCTGGTGTTATGGACATGGAAATCAACGCTATCGGAAGCCAGC
6-798  GTAGCGTCATCAGTGGGCGCCCTAGCGGCGCAAGCGTACCAAAGCCTGTTCGCCAATTCTGGTGTTATGGACATGGAAATCAACGCTATCGGAAGCCAGC
                                                   Exon 17

       3101                    3125                     3150                     3175                     3200
GR     CAATAGAAGAATATTATCTAAATAGAACAAGTGATCCCGTTGTGATGGGTTCGCTGCGGCACCGCTTGCTGATAGGCTCCACATTTGACGACGACTCTGC
GR-1   CAATAGAAGAATATTATCTAAATAGAACAAGTGATCCCGTCGTGATGGGTTCGCTGCGGCACCGCTTGCTGATAGGCTCCACATTTGACGACGACTCAGC
GR-44  CAATAGAAGAATATTATCTAAATAGAACAAGTGATCCCGTCGTGATGGGTTCGCTGCGGCACCGCTTGCTGATAGGCTCCACATTTGACGACGACTCAGC
GR-45  CAATAGAAGAATATTATCTAAATAGAACAAGTGATCCCGTCGTGATGGGTTCGCTGCGGCACCGCTTGCTGATAGGCTCCACATTTGACGACGACTCAGC
GR-46  CAATAGAAGAATATTATCTAAATAGAACAAGTGATCCCGTCGTGATGGGTTCGCTGCGGCACCGCTTGCTGATAGGCTCCACATTTGACGACGACTCAGC
SP15   CAATAGAAGATTATTATCTAAATAGAACAAGTGATCCCGTTGTGATGGGTTCGCTGCGGCACCGCTTGCTGATAGGCTCCACATTTGACGACGACTCTGC
6-364  CAATAGAAAAATATTATCTAAATAGA-----TGATCCCGTTGTGATGGGTTCGCTGCGGCACCGCTTGCTGATAGGCTCCACATTTGACGACGACTCTGC
6-798  CAATAGAAAAATATTATCTAAATAGAACAAGTGATCCCGTTGTGATGGGTTCGCTGCGGCACCGCTTGCTGATAGGCTCCACATTTGACGACGACTCTGC
      P/A        Exon 17                                              Exon 18

       3201                    3225                     3250                     3275                     3300
GR     TACCGCCTGGTTCAGTAATTTCGGCTACCACGATGTTGCTACATCGCTTGCGGCAATCCACTCAGCTATTCTCAGATCTAAAAACTCGGATGCAGTACTC
GR-1   TACCGCCTGGTTCAGTAATTTCGGCTACCACGATGTTGCTACATCGCTTGCGGCAATCCACTCAGCTATTCTCAGATCTAAAAACTCGGATGCAGTACTC
GR-44  TACCGCCTGGTTCAGTAATTTCGGCTACCACGATGTTGCTACATCGCTTGCGGCAATCCACTCAGCTATTCTCAGATCTAAAAACTCGGATGCAGTACTC
GR-45  TACCGCCTGGTTCAGTAATTTCGGCTACCACGATGTTGCTACATCGCTTGCGGCAATCCACTCAGCTATTCTCAGATCTAAAAACTCGGATGCAGTACTC
GR-46  TACCGCCTGGTTCAGTAATTTCGGCTACCACGATGTTGCTACATCGCTTGCGGCAATCCACTCAGCTATTCTCAGATCTAAAAACTCGGATGCAGTACTC
SP15   TACCGCCTGGTTCAGTAATTTCGGCTACCACGATGTTGCGACATCGCTTGCGGCAATCCACTCGGCTATTCTCAGATCTAAAAACTCTGATGCAGTACTC
6-364  TACCGCCTGGTTCAGTAATTTCGGCTACCACGATGTTGCTACATCGCTTGCGGCAATTCACTCGGCTATTCTCAGATCTAAAAACTCGGATGCAGTACTC
6-798  TACCGCCTGGTTCAGTAATTTCGGCTACCACGATGTTGCTACATCGCTTGCGGCAATTCACTCGGCTATTCTCAGATCTAAAAACTCGGATGCAGTACTC
                                                   Exon 18

       3301                    3325                     3350                     3375                     3400
GR     AATGTATATAATCATCCCTTGGAAGCTTCTTACATAGATCAGAGTGACGTGCAGACTATGATAGCTATGTTGTCCATGCAGTTGTCCTCTGGCATCGGCA
GR-1   AATGTATATAATCATCCGTTGGAAGCTTCTTACATAGATCAGAGTGACGTGCAGACTATGATAGCTATGTTGTCCATGCAGTTGTCCTCTGGCATCGGCA
GR-44  AATGTATATAATCATCCGTTGGAAGCTTCTTACATAGATCAGAGTGACGTGCAGACTATGATAGCTATGTTGTCCATGCAGTTGTCCTCTGGCATCGGCA
GR-45  AATGTATATAATCATCCGTTGGAAGCTTCTTACATAGATCAGAGTGACGTGCAGACTATGATAGCTATGTTGTCCATGCAGTTGTCCTCTGGCATCGGCA
GR-46  AATGTATATAATCATCCGTTGGAAGCTTCTTACATAGATCAGAGTGACGTGCAGACTATGATAGCTATGTTGTCCATGCAGTTGTCCTCTGGCATCGGCA
SP15   AATGTATACAATCATCCCTTGGAAGCTTCGTATATAGATCAGAGTGACGTGCAGACTATGATAGCTATGTTGTCCATGCAGTTGTCCTCTGGCATCGGCA
6-364  AATGTATATAATCATCCCTTGGAAGCTTCTTACATAGATCAGAGTGACGTGCAGACTATGATAGCTATGTTGTCCATGCAGTTGTCCTCTGGCATCGGCA
6-798  AATGTATATAATCATCCCTTGGAAGCTTCTTACATAGATCAAAGTGACGTGCAAACTATGATAGCTATGTTGTCCATGCAGTTGTCCTCTGGCATCGGCA
                         Exon 18                                            Exon 19

       3401                    3425                     3450                     3475                     3500
GR     GTAGTGTGAGCATTGTTAGTGCGGTTTTCATCATGTTTTATATCAAGGAACGTATGTCGGGGGCAAAACTTCTACAAAATGCAGCAGGCGTAGCGCCTGC
GR-1   GTAGTGTGAGCATTGTTAGTGCGGTTTTCATCATGTTTTATATCAAGGAACGTATGTCGGGGGCTAAACTTCTACAAAATGCAGCAGGCGTGGCGCCTGC
GR-44  GTAGTGTGAGCATTGTTAGTGCGGTTTTCATCATGTTTTATATCAAGGAACGTATGTCGGGGGCTAAACTTCTACAAAATGCAGCAGGCGTGGCGCCTGC
GR-45  GTAGTGTGAGCATTGTTAGTGCGGTTTTCATCATGTTTTATATCAAGGAACGTATGTCGGGGGCTAAACTTCTACAAAATGCAGCAGGCGTGGCGCCTGC
GR-46  GTAGTGTGAGCATTGTTAGTGCGGTTTTCATCATGTTTTATATCAAGGAACGTATGTCGGGGGCTAAACTTCTACAAAATGCAGCAGGCGTGGCGCCTGC
SP15   GTAGTGTGAGCATTGTTAGTGCTGTGTTCATCATGTTTTATATCAAGGAACGTATGTCTGGGGCTAAACTTCTACAAAATGCAGCAGGCGTAGCGCCTGC
6-364  GTAGTGTGAGCATTGTTAGTGCGGTTTTCATCATGTTTTATATCAAGGAACGTATGTCGGGGGCAAAACTTCTACAAAATGCAGCAGGCGTAGCGCCTGC
6-798  GTAGTGTGAGCATTGTTAGTGCGGTTTTCATCATGTTTTATATCAAGGAACGTATGTCGGGGGCAAAACTTCTACAAAATGCAGCAGGCGTAGCGCCTGC
                           Exon 19                                                 Exon 20

       3501                    3525                     3550                     3575                     3600
GR     TGTGCTGTGGGGCGGCGCAGCGATCTTCAATTGGTTTTGGTTCCTCATCACTTGTGTTTCCATCGTCATCTCGTGCGTCGCTTTTGATGTCATCGGGTTG
GR-1   TGTGCTGTGGGGCGGCGCAGCGATCTTCAATTGGTTTTGGTTCCTCATCACTTGTGTTTCCATCGTCATCTCGTGCGTCGCTTTTGATGTCATCGGGTTG
GR-44  TGTGCTGTGGGGCGGCGCAGCGATCTTCAATTGGTTTTGGTTCCTCATCACTTGTGTTTCCATCGTCATCTCGTGCGTCGCTTTTGATGTCATCGGGTTG
GR-45  TGTGCTGTGGGGCGGCGCAGCGATCTTCAATTGGTTTTGGTTCCTCATCACTTGTGTTTCCATCGTCATCTCGTGCGTCGCTTTTGATGTCATCGGGTTG
GR-46  TGTGCTGTGGGGCGGCGCAGCGATCTTCAATTGGTTTTGGTTCCTCATCACTTGTGTTTCCATCGTCATCTCGTGCGTCGCTTTTGATGTCATCGGGTTG
SP15   TGTGCTGTGGGGCGGCGCAGCGATCTTCAATTGGTTTTGGTTCCTCATCACTTGTGTTTCCATCGTCATCTCGTGCGTCGCTTTTGATGTCATCGGGTTG
6-364  TGTGCTGTGGGGCGGCGCAGCGATCTTCAATTGGTTTTGGTTCCTCATCACTTGTGTTTCCATCGTCATCTCGTGCGTCGCTTTTGATGTCATCGGGTTG
6-798  TGTGCTGTGGGGCGGCGCAGCGATCTTCAATTGGTTTTGGTTCCTCATCACTTGTGTTTCCATCGTCATCTCGTGCGTCGCTTTTGATGTCATCGGGTTG
                                                   Exon 20
```

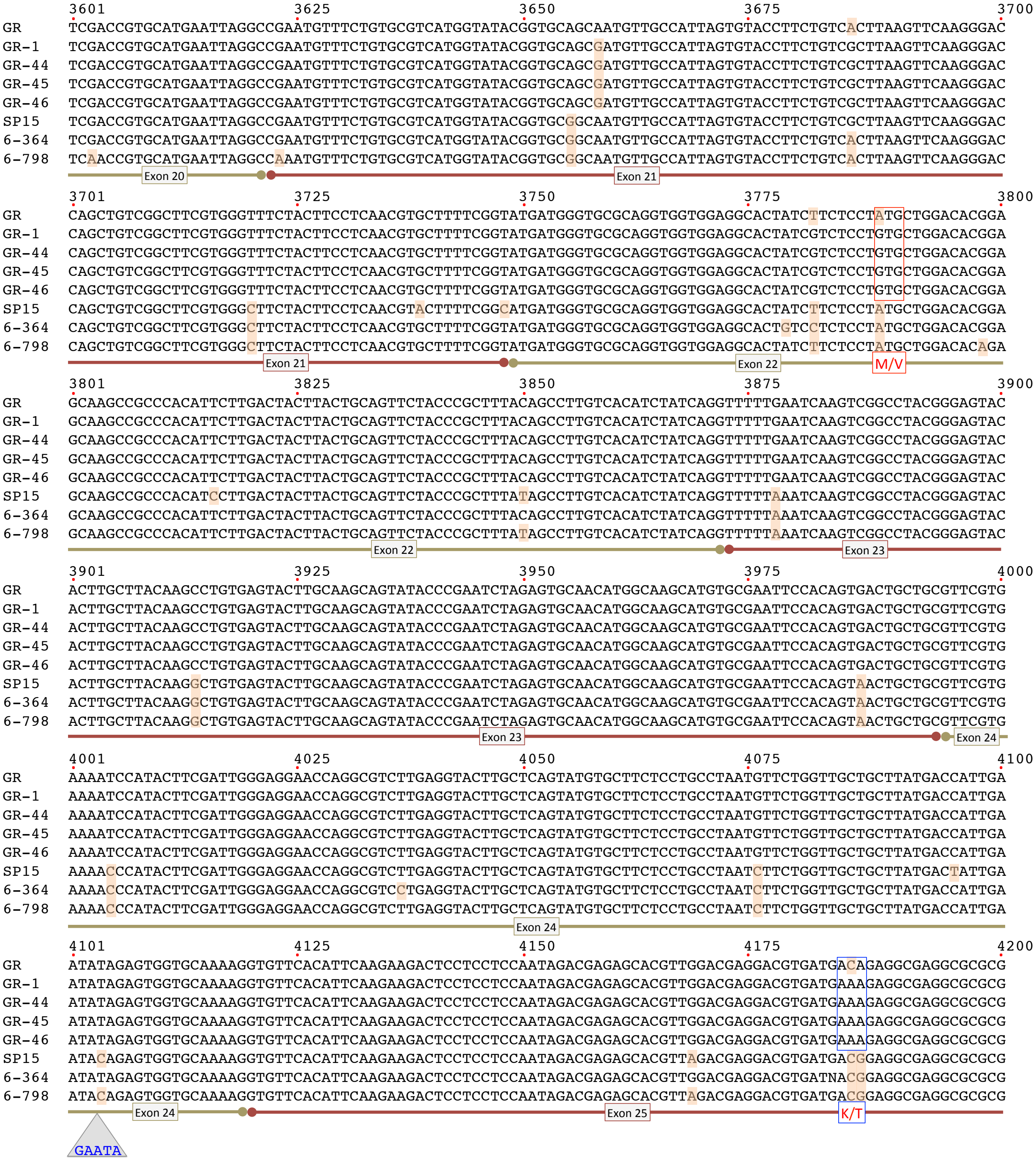
3601
3625
3650
3675
3700
GR
GR-1
GR-44
GR-45
GR-46
SP15
6-364
6-798
Exon 20
Exon 21
Exon 22
M/V
Exon 23
Exon 24
Exon 25
K/T
GAATA
3701
3725
3750
3775
3800
3801
3825
3850
3875
3900
3901
3925
3950
3975
4000
4001
4025
4050
4075
4100
4101
4125
4150
4175
4200

```
       4201                      4225                     4250                     4275                          4300
GR     CGCGTGGTGCCACCGACACGACGCCGCGAACACGCCCTTCTAGCTCACGACCTCTCCAAGTACTACGGGAAACATCTCGCCGTAGACCAAGTCTCGTTTA
GR-1   CGCGTGGTGCCACCGACACGACGCCGCGAACACGCCCTTCTAGCTCACGACCTCTCCAAGTACTACGGGAAACATCTCGCCGTAGACCAAGTTTCGTTTA
GR-44  CGCGTGGTGCCACCGACACGACGCCGCGAACACGCCCTTCTAGCTCACGACCTCTCCAAGTACTACGGGAAACATCTCGCCGTAGACCAAGTTTCGTTTA
GR-45  CGCGTGGTGCCACCGACACGACGCCGCGAACACGCCCTTCTAGCTCACGACCTCTCCAAGTACTACGGGAAACATCTCGCCGTAGACCAAGTTTCGTTTA
GR-46  CGCGTGGTGCCACCGACACGACGCCGCGAACACGCCCTTCTAGCTCACGACCTCTCCAAGTACTACGGGAAACATCTCGCCGTAGACCAAGTTTCGTTTA
SP15   CGCGTGGTGCCACCGACACGCCGCAGCGAACACGCACTTCTCGCTCACGACCTCTCCAAGTACTACGGGAAACATCTCGCCGTAGACCAAGTCTCGTTTA
6-364  CGCGTGGTGCCACCGACACGCCGCAGCGACCACGCGCTTCTCGCTCACGACCTCTCCAAGTACTACGGGAAACATCTCGCCGTAGACCAAGTCTCGTTTA
6-798  CGCGTGGTGCCACCGACACGCCGCAGCGACCACGCGCTTCTCGCTCACGACCTCTCCAAGTACTACGGGAAACATCTCGCCGTAGACCAAGTCTCGTTTA
                                                   Exon 25

       4301                      4325                     4350                     4375                          4400
GR     GTGTGAACGACGGCGAATGCTTCGGTCTATTGGGTGTGAATGGTGCCGGAAAAACAACCACCTTCAAGATGCTGATGGGTGATGAGTCCATTTCAAGCGG
GR-1   GTGTGAACGACGGCGAATGCTTCGGTCTATTGGGTGTGAATGGTGCCGGAAAAACAACCACCTTCAAGATGCTGATGGGTGATGAGTCCATTTCAAGCGG
GR-44  GTGTGAACGACGGCGAATGCTTCGGTCTATTGGGTGTGAATGGTGCCGGAAAAACAACCACCTTCAAGATGCTGATGGGTGATGAGTCCATTTCAAGCGG
GR-45  GTGTGAACGACGGCGAATGCTTCGGTCTATTGGGTGTGAATGGTGCCGGAAAAACAACCACCTTCAAGATGCTGATGGGTGATGAGTCCATTTCAAGCGG
GR-46  GTGTGAACGACGGCGAATGCTTCGGTCTATTGGGTGTGAATGGTGCCGGAAAAACAACCACCTTCAAGATGCTGATGGGTGATGAGTCCATTTCAAGCGG
SP15   GTGTGAACGACGGCGAATGCTTCGGTCTATTGGGTGTGAATGGTGCCGGAAAAACGACCACCTTCAAGATGCTGATGGGTGATGAGTCCATTTCAAGCGG
6-364  GTGTGAACGACGGCGAATGCTTCGGTCTATTGGGTGTGAATGGTGCAGGAAAAACGACCACCTTCAAGATGCTGATGGGTGATGAGTCCATTTCAAGCGG
6-798  GTGTGAACGACGGCGAATGCTTCGGTCTATTGGGTGTGAATGGTGCAGGAAAAACGACCACCTTCAAGATGCTGATGGGCGATGAGGCCATTTCAAGCGG
                                                    Exon 26

       4401                      4425                     4450                     4475                          4500
GR     CGAGGCGTATGTCTCCGGGCACTCGGTGCAGAGGAATCTTGATAGAGTGCATGAGAATATTGGATATTGTCCGCAATTTGACGCATTATTTGGTGAGCTG
GR-1   CGAGGCGTATGTCTCCGGGCACTCGGTGCAGAGGAATCTTGATAGAGTACACGAGAATATTGGATATTGTCCGCAATTTGATGCATTATTTGGTGAGCTG
GR-44  CGAGGCGTATGTCTCCGGGCACTCGGTGCAGAGGAATCTTGATAGAGTACACGAGAATATTGGATATTGTCCGCAATTTGATGCATTATTTGGTGAGCTG
GR-45  CGAGGCGTATGTCTCCGGGCACTCGGTGCAGAGGAATCTTGATAGAGTACACGAGAATATTGGATATTGTCCGCAATTTGATGCATTATTTGGTGAGCTG
GR-46  CGAGGCGTATGTCTCCGGGCACTCGGTGCAGAGGAATCTTGATAGAGTACACGAGAATATTGGATATTGTCCGCAATTTGATGCATTATTTGGTGAGCTG
SP15   CGAGGCGTATGTCTCCGGGCACTCGGTGCAGAGGAATCTTGATAGAGTACACGAGAATATTGGATATTGTCCGCAATTTGACGCATTATTTGGTGAGCTG
6-364  CGAGGCGTATGTCTCCGGGCACTCGGTGCAGAGGAATCTCGATAGAGTACACGAGAATATTGGATATTGTCCGCAATTTGATGCATTATTTGGTGAGCTG
6-798  CGAGGCGTATGTCTCCGGGCACTCGGTGCAGAGGAATCTCGATAGAGTGCATGAGAATATTGGATATTGTCCGCAATTTGACGCATTATTTGGTGAGCTG
                                 Exon 26                                               Exon 27

       4501                      4525                     4550                     4575                          4600
GR     ACGGGTCGCCAGACACTCCACATGTTTGCGTTGATGCGCGGCTTGCGTTTGCGCACTGCGGCACCCTCAGCTGAAACACTCGCACATGCGCTTGGCTTCT
GR-1   ACGGGTCGCCAGACACTCCACATGTTTGCGTTGATGCGCGGCTTGCGTTTGCGCACTGCGGCGCCCTCAGCTGAAACACTCGCACATGCGCTTGGCTTCT
GR-44  ACGGGTCGCCAGACACTCCACATGTTTGCGTTGATGCGCGGCTTGCGTTTGCGCACTGCGGCGCCCTCAGCTGAAACACTCGCACATGCGCTTGGCTTCT
GR-45  ACGGGTCGCCAGACACTCCACATGTTTGCGTTGATGCGCGGCTTGCGTTTGCGCACTGCGGCGCCCTCAGCTGAAACACTCGCACATGCGCTTGGCTTCT
GR-46  ACGGGTCGCCAGACACTCCACATGTTTGCGTTGATGCGCGGCTTGCGTTTGCGCACTGCGGCGCCCTCAGCTGAAACACTCGCACATGCGCTTGGCTTCT
SP15   ACGGGTCGCCAGACACTACACATGTTTGCGTTGATGCGCGGCTTGCGTTTGCGCACTGCGGCACCCTCAGCTGAAACACTCGCACATGCGCTTGGCTTCT
6-364  ACGGGTCGCCAGACACTACACATGTTTGCGTTGATGCGCGGCTTGCGTTTGCGCACTGCGGCGCCCTCAGCTGAAACACTCGCACATGCGCTTGGCTTCT
6-798  ACGGGTCGCCAGACACTACACATGTTTGCGTTGATGCGCGGCTTGCGTTTGCGCACTGCGGCACCCTCAGCTGAAACACTCGCACATGCGCTTGGCTTCT
                                                   Exon 27

       4601                      4625                     4650                     4675                          4700
GR     TCAAACATCTCGATAAAAGGGTGCATCAGTATTCAGGCGGCACGAAACGCAAGCTTAACACGGCGATAGCATTCATGGGACGAACACGGCTTGTGTTTGT
GR-1   TCAAACATCTCGATAAAAGGGTGCATCAGTATTCAGGCGGCACGAAACGCAAGCTTAACACGGCGATAGCATTCATGGGACGAACACGGCTTGTGTTTGT
GR-44  TCAAACATCTCGATAAAAGGGTGCATCAGTATTCAGGCGGCACGAAACGCAAGCTTAACACGGCGATAGCATTCATGGGACGAACACGGCTTGTGTTTGT
GR-45  TCAAACATCTCGATAAAAGGGTGCATCAGTATTCAGGCGGCACGAAACGCAAGCTTAACACGGCGATAGCATTCATGGGACGAACACGGCTTGTGTTTGT
GR-46  TCAAACATCTCGATAAAAGGGTGCATCAGTATTCAGGCGGCACGAAACGCAAGCTTAACACGGCGATAGCATTCATGGGACGAACACGGCTTGTGTTTGT
SP15   TCAAACATCTCGATAAAAGGGTGCATCAGTATTCAGGTGGAACGAAACGCAAGCTTAACACGGCGATAGCATTCATGGGACGAACACGGCTTGTGTTTGT
6-364  TCAAACATCTCGATAAAAGGGTGCATCAGTATTCAGGTGGAACGAAACGCAAGCTTAACACGGCGATAGCATTCATGGGACGAACACGGCTTGTGTTTGT
6-798  TCAAACATCTAGATAAAAGGGTGCATCAGTATTCAGGCGGCACGAAACGCAAGCTTAACACGGCGATAGCATTCATGGGACGAACACGGCTTGTGTTTGT
             Exon 27                                                  Exon 28

       4701                      4725                     4750                     4775                          4800
GR     TGATGAGCCTACCACTGGAGTCGATCCCGCCGCTAAACGCCACGTATGGCGCGCTACCCGCGGCGTGCAGCGAGCAGGTCGCGGTGTGGTACTGACGTCA
GR-1   TGATGAGCCTACAACTGGAGTCGATCCCGCCGCTAAACGCCACGTATGGCGCGCTACCCGCGGCGTGCAGCGAGCAGGTCGCGGCGTGGTACTGACGTCA
GR-44  TGATGAGCCTACAACTGGAGTTGATCCCGCCGCTAAACGCCACGTATGGCGCGCTACCCGCGGCGTGCAGCGAGCAGGTCGCGGCGTGGTACTGACGTCA
GR-45  TGATGAGCCTACAACTGGAGTCGATCCCGCCGCTAAACGCCACGTATGGCGCGCTACCCGCGGCGTGCAGCGAGCAGGTCGCGGCGTGGTACTGACGTCA
GR-46  TGATGAGCCTACAACTGGAGTCGATCCCGCCGCTAAACGCCACGTATGGCGCGCTACCCGCGGCGTGCAGCGAGCAGGTCGCGGCGTGGTACTGACGTCA
SP15   TGATGAGCCTACAACTGGAGTTGATCCCGCCGCTAAACGCCACGTATGGCGCGCTACCCGCGGCGTGCAGCGAGCAGGTCGCGGCGTGGTGCTGACGTCA
6-364  TGATGAGCCTACAACTGGAGTTGATCCCGCCGCTAAACGCCACGTATGGCGCGCTACCCGCGGCGTGCAGCGAGCAGGTCGCGGTGTGGTACTGACGTCA
6-798  TGATGAGCCTACAACTGGAGTTGATCCCGCCGCTAAACGCCACGTATGGCGCGCTACCCGCGGCGTGCAGCGAGCAGGTCGCGGTGTGGTACTGACGTCA
                               Exon 28                                       Exon 29
```

```
       4801                     4825                     4850                     4875                     4900
GR     CACAGTATGGAGGAGTGCGAGGCGTTGTGTTCGCGACTGACCATCATGGTCAATGGTCGCTTCCAGTGTCTGGGCACGCCACAACATCTCAAGAACAAAT
GR-1   CACAGTATGGAGGAGTGCGAGGCGTTGTGTTCGCGACTGACCATCATGGTCAATGGTCGCTTTCAGTGTCTGGGCACGCCACAACATCTCAAGAACAAAT
GR-44  CACAGTATGGAGGAGTGCGAGGCGTTGTGTTCGCGACTGACCATCATGGTCAATGGTCGCTTTCAGTGTCTGGGCACGCCACAACATCTCAAGAACAAAT
GR-45  CACAGTATGGAGGAGTGCGAGGCGTTGTGTTCGCGACTGACCATCATGGTCAATGGTCGCTTTCAGTGTCTGGGCACGCCACAACATCTCAAGAACAAAT
GR-46  CACAGTATGGAGGAGTGCGAGGCGTTGTGTTCGCGACTGACCATCATGGTCAATGGTCGCTTTCAGTGTCTGGGCACGCCACAACATCTCAAGAACAAAT
SP15   CATAGCATGGAGGAGTGCGAGGCGTTGTGTTCGCGACTGACCATCATGGTCAATGGTCGCTTCCAGTGTCTGGGAACGCCACAACATCTCAAGAACAAAT
6-364  CATAGCATGGAGGAGTGCGAGGCGTTGTGTTCGCGACTGACCATCATGGTCAATGGTCGCTTCCAGTGTTTGGGCACGCCACAACATCTCAAGAACAAAT
6-798  CACAGTATGGAGGAGTGCGAGGCGTTGTGTTCGCGGCTGACCATCATGGTCAATGGTCGCTTCCAGTGTCTGGGAACGCCACAACATCTCAAGAACAAAT
                                                   Exon 29

       4901                     4925                     4950                     4975                     5000
GR     TCTCTCAAGGCTTTACTCTAATCATAAAAATGAAAACTGACGACAGTGAAGGTGACACGCAGTCAGTAAACAGCACTACCAGCGTAGTAGATAGTGTCAA
GR-1   TCTCTCAAGGCTTTACTTTAATCATAAAAATGAAAACTGACGACAGTGAAAGTGACACGCAGTCAGTAAACAGCACAACCAGCATAGTAGATAGTGTCAA
GR-44  TTTCTCAAGGCTTTACTTTAATCATAAAAATGAAAACTGACGACAGTGAAAGTGACACGCAGTCAGTAAACAGCACAACCAGCATAGTAGATAGTGTCAA
GR-45  TCTCTCAAGGCTTTACTTTAATCATAAAAATGAAAACTGACGACAGTGAAAGTGACACGCAGTCAGTAAACAGCACAACCAGCATAGTAGATAGTGTCAA
GR-46  TCTCTCAAGGCTTTACTTTAATCATAAAAATGAAAACTGACGACAGTGAAAGTGACACGCAGTCAGTAAACAGCACAACCAGCATAGTAGATAGTGTCAA
SP15   TTTCTCAAGGCTTTACTTTAATCATAAAAATGAAAACTGACGACACAGAAAGTGACACGCAGTCAGTAAACAGCACAACCAGCATAGTGGATAGTGTCAA
6-364  TTTCTCAAGGCTTTACGTTAATCATAAAAATGAAAACTGACGACAGTGAAAGTGACACGCAGTCAGTAAACAGCACTACCAGCATAGCAGATAGTGTCAA
6-798  TTTCTCAAGGCTTTACGTTAATCATAAAAATGAAAACTGACGACAGTGAAAGTGACACACAGTCAGTAAACAGCACAACCAGCATAGTAGATAGTGTCAA
        Exon 29                                         Exon 30                         I/V

       5001                     5025                     5050                     5075                     5100
GR     ACTATACGTCTCTGAGAACTTTGAAAGTCCAAAGATAATGGAAGAATATCACGGTCTTCTGACTTACTACTTGCCTGACCGAAGCATGGCATGGTCACGA
GR-1   ACTATACGTCTCTGAGAACTTTGAAAGTCCAAAGATAATGGAAGAATATCACGGTCTTCTGACTTACTACTTGCCTGACCGTAGCATGGCATGGTCACGA
GR-44  ACTATACGTCTCTGAGAACTTTGAAAGTCCAAAGATAATGGAAGAATATCACGGTCTTCTGACTTACTACTTGCCTGACCGTAGCATGGCATGGTCACGA
GR-45  ACTATACGTCTCTGAGAACTTTGAAAGTCCAAAGATAATGGAAGAATATCACGGTCTTCTGACTTACTACTTGCCTGACCGTAGCATGGCATGGTCACGA
GR-46  ACTATACGTCTCTGAGAACTTTGAAAGTCCAAAGATAATGGAAGAATATCACGGTCTTCTGACTTACTACTTGCCTGACCGTAGCATGGCATGGTCACGA
SP15   ACTATACGTCTCCGAGAACTTCGAAAGTCCAAAGATAATGGAAGAGTATCACGGTCTTCTAACCTACTACTTGCCTGACCGTAGCATGGCATGGTCACGA
6-364  ACTATACGTCTCTGAGAACTTTGAAAGTCCAAAGATAATGGAAGAGTATCACGGTCTTCTAACTTACTACTTGCCTGACCGTAGCATGGCATGGTCACGA
6-798  ACTATACGTCTCTGAGAACTTTGAAAGTCCAAAGATAATGGAAGAATATCACGGTCTTCTAACCTACTACTTGCCTGACCGTAGCATGGCATGGTCACGA
                      Exon 30                                         Exon 31

       5101                     5125                     5150                     5175                     5200
GR     ATGTTTGGTATAATGGAGCGCGCCAAACAGATCTTACAAATTGAGGACTACAGCATATCGCAGACTACCCTCGAACAAATATTCTTGCAGTTCACCAAAT
GR-1   ATGTTTGGTATAATGGAGCGCGCCAAACAGATCTTACAAATTGAGGACTACAGCATATCGCAGACTACCCTCGAACAAATATTCTTGCAGTTCACCAAAT
GR-44  ATGTTTGGTATAATGGAGCGCGCCAAACAGATCTTACAAATTGAGGACTACAGCATATCGCAGACTACCCTCGAACAAATATTCTTGCAGTTCACCAAAT
GR-45  ATGTTTGGTATAATGGAGCGCGCCAAACAGATCTTACAAATTGAGGACTACAGCATATCGCAGACTACCCTCGAACAAATATTCTTGCAGTTCACCAAAT
GR-46  ATGTTTGGTATAATGGAGCGCGCCAAACAGATCTTACAAATTGAGGACTACAGCATATCGCAGACTACCCTCGAACAAATATTCTTGCAGTTCACCAAAT
SP15   ATGTTTGGTATCATGGAGCGCGCCAAACAGATCTTACAAATTGAGGACTACAGCATATCGCAGACTACCCTCGAACAAATATTCTTGCAGTTCACCAAAT
6-364  ATGTTTGGTATAATGGAGCGCGCCAAACAGATCTTACAAATTGAGGACTACAGCATATCGCAGACTACCCTCGAACAAATATTCTTGCAGTTCACCAAAT
6-798  ATGTTTGGTATCATGGAGCGCGCCAAACAGATCTTACAAATTGAGGACTACAGCATATCGCAGACTACCCTCGAACAAATATTCTTGCAGTTCACCAAAT
                                                   Exon 31

       5201                     5225
GR     ACCAAAGAGAAGAAGGAACGACGTTA
GR-1   ACCAAAGAGAAGAAGGAACGACGTTA
GR-44  ACCAAAGAGAAGAAGGAACGACGTTA
GR-45  ACCAAAGAGAAGAAGGAACGACGTTA
GR-46  ACCAAAGAGAAGAAGGAACGACGTTA
SP15   ACCAAAGAGAAGAAGGAACGACGTTA
6-364  ACCAAAGAGAAGAAGGAACGACGTTA
6-798  ACCAAAGAGAAGAAGGAACGACGTTA
               Exon 31
```
